# Porous and Meltable Metal–Organic Polyhedra for the Generation and Shaping of Porous Mixed-Matrix Composites

**DOI:** 10.1021/jacs.4c00407

**Published:** 2024-03-11

**Authors:** Cornelia von Baeckmann, Jordi Martínez-Esaín, José A. Suárez del Pino, Lingxin Meng, Joan Garcia-Masferrer, Jordi Faraudo, Jordi Sort, Arnau Carné-Sánchez, Daniel Maspoch

**Affiliations:** † Catalan Institute of Nanoscience and Nanotechnology (ICN2), CSIC, and The Barcelona Institute of Science and Technology, Campus UAB, 08193 Bellaterra, Spain; ‡ Departament de Química, Facultat de Ciències, Universitat Autònoma de Barcelona, 08193 Bellaterra, Spain; § Institut de Ciència de Materials de Barcelona (ICMAB-CSIC), 08193 Bellaterra, Spain; ∥ Departament de Física, Universitat Autònoma de Barcelona, 08193 Bellaterra, Spain; ⊥ ICREA, Pg. Lluís Companys 23, 08010 Barcelona, Spain

## Abstract

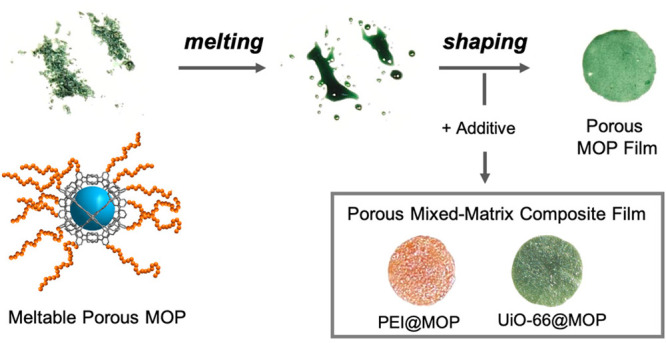

Here, we report the synthesis of BCN-93, a meltable, functionalized, and permanently porous metal–organic polyhedron (MOP) and its subsequent transformation into amorphous or crystalline, shaped, self-standing, transparent porous films via melting and subsequent cooling. The synthesis entails the outer functionalization of a MOP with meltable polymer chains: in our model case, we functionalized a Rh(II)-based cuboctahedral MOP with poly(ethylene glycol). Finally, we demonstrate that once melted, BCN-93 can serve as a porous matrix into which other materials or molecules can be dispersed to form mixed-matrix composites. To illustrate this, we combined BCN-93 with one of various additives (either two MOF crystals, a porous cage, or a linear polymer) to generate a series of mixed-matrix films, each of which exhibited greater CO_2_ uptake relative to the parent film.

Melting has long been a widely employed method for transforming raw materials into shaped objects across various industries.^1−4^ Recently, the application of melting as a processing technique for porous materials such as metal–organic frameworks (MOFs) has presented a unique opportunity to mold these materials into novel forms, including neat porous liquids and glasses.^5−10^ Unfortunately, the current range of meltable MOFs is limited, as most MOFs decompose before they could even melt.^11−15^ Additionally, the melting of MOFs involves the rupture of coordination bonds that define the MOF structure, leading to uncertainty regarding the final structure, porosity and—by extension—function of the melted product.^16−18^

Herein we present a novel approach to transform metal–organic polyhedra (MOPs) into metal–organic films with a persistent and designed porosity. Our approach begins with densely grafting polymer chains onto the surface of a robust MOP. In the resultant functionalized MOP, the parent MOP behaves as the persistent pore unit, whereas the polymeric shell imparts meltability. Thus, since the intrusion of surface polymer chains into the MOP cavity is inhibited, these functionalized MOPs can be transformed into porous films through melt-quenching (Figure 1). Importantly, the resultant films are free from grain boundaries, amorphous or semicrystalline, shaped, and self-standing. Additionally, once melted, the same functionalized MOP can be used as a solvent or matrix into which other species can be dissolved or dispersed to generate porous, mixed-matrix composites with unique structures and functions.

**Figure 1:**
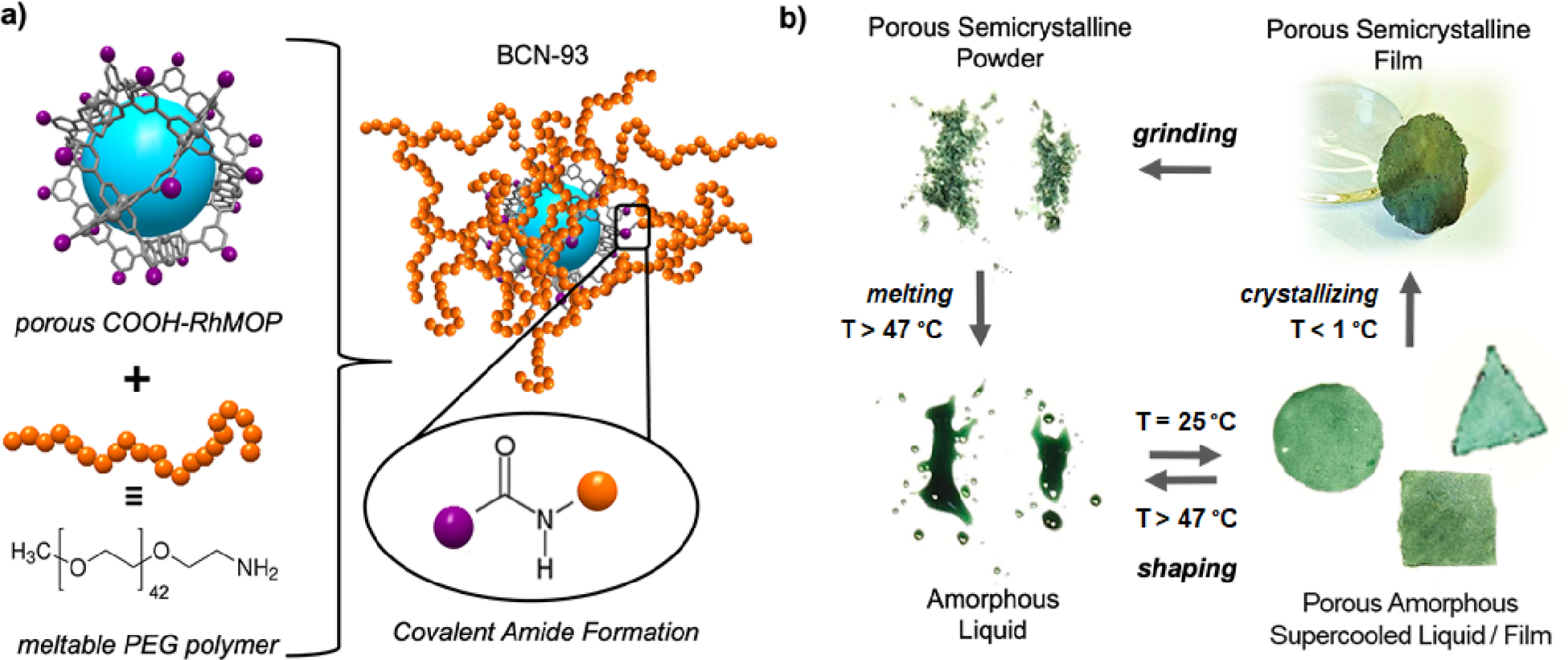
(a) Schematic of the synthesis of meltable, porous MOP BCN-93 via formation of an amide bond between the surface carboxylic acid groups of the parent Rh(II)-MOP and the terminal amino groups of NH_2_–PEG2000 chains. (b) Schematic of the melting-cooling of BCN-93 to produce amorphous or crystalline-shaped porous films.

We began by choosing Rh(II)-based cuboctahedral MOPs (Rh-MOP) as our pore unit, due to its high structural and chemical stability as well as its rich surface chemistry,^19,20^ and an amine-terminated poly(ethylene glycol) (PEG) chain (*ca*. 2000 g mol^–1^) as the meltable polymer.^21−29^ The PEG chain was covalently grafted onto the surface of a carboxylic acid-functionalized Rh-MOP (COOH-RhMOP)^30^ via formation of an amide bond (Figure 1a). The preservation of the Rh(II) paddlewheel throughout the reaction was confirmed by UV/vis (Figure S1). The resulting PEG-functionalized MOP (hereafter named BCN-93, Figure 1a) was first characterized through ^1^H NMR spectroscopy in methanol-d_4_, which revealed the expected peaks: the aromatic signals of the MOP core and the aliphatic signals of the PEG chains (Figures S2 and S3). The two sets of peaks exhibited the same diffusion coefficient (7.6 × 10^–11^ m^2^ s^–1^) in the Diffusion Ordered Spectroscopy (DOSY) ^1^H NMR spectrum (Figure S2b), which confirmed the linkage between the MOP and the PEG chains, and the absence of free PEG in the sample. The hydrodynamic radius was calculated to be 5.25 nm. Conversion of the negatively charged surface carboxylic acid groups (at pH 7) into neutral amide groups was further confirmed by Z-potential measurements: the Z-potential value evolved from −45 mV in the parent MOP to −9 mV after PEGylation (Figure S4). The degree of conversion of the surface carboxylic acid groups was found to be 100%, as determined by ^1^H NMR analysis of the acid-digested sample (Figure S5). This allowed us to propose the following molecular formula for BCN-93: [Rh_24_(PEG_2000_-BDC)_24_] (where PEG_2000_-BDC is the PEGylated benzenedicarboxylic acid ligand). This formula was further confirmed through matrix-assisted laser desorption/ionization-time-of-flight (MALDI-TOF) spectrometry, which revealed a peak at *m*/*z* 53272 (Figure S6) for BCN-93 (expected: 55513 ± 4800). The obtained BCN-93 exhibited a broad solubility profile in organic and aqueous solvents, as confirmed by UV/vis spectroscopy (Figures S7). Thermogravimetric analysis (TGA) of BCN-93 indicated that it is stable up to 400 °C (Figure S8). Finally, X-ray powder diffraction (XRPD) analysis of the as-made BCN-93 revealed that it is a crystalline compound with two sharp diffraction peaks, at 2θ = 19.3° and 23.5°, which we ascribed to the semicrystalline nature of the surface PEG chains (Figure S9).^31^

We then sought to further explore the thermal behavior of BCN-93. Remarkably, upon heating at 47 °C, it undergoes concomitant melting and amorphization (Figure 1b: compare top-left to bottom-left), as demonstrated by Differential Scanning Calorimetry (DSC), variable temperature (VT)-PXRD, and (VT)-Field Emission Scanning Electron Microscopy (FE-SEM) (Figure 2). The change in the physical state of BCN-93 upon heating occurs in its neat state and does not entail weight loss, as confirmed by TGA (Figure S8). Upon cooling below 1 °C, BCN-93 recovers its crystalline character. The radial organization of PEG chains on the surface of BCN-93 hinders their crystallization: consequently, both the crystallinity and the crystallization temperature of BCN-93 are lower than those of free amino-PEG2000 chains (Figure S10). Thus, BCN-93 presents a large thermal hysteresis between its amorphous and crystalline states, which enables its processing into two different physical states at room temperature: a semicrystalline material, when it is synthesized or when, after melting, it is cooled below 1 °C and then heated to room temperature; and an amorphous material, when, after melting, it is cooled to room temperature (Figures S11 and S12). Indeed, thermal quenching of the melted BCN-93 produces an amorphous kinetically trapped metastable phase that can be considered as a supercooled liquid.^32^ The integrity of BCN-93 upon melting and cooling was confirmed by ^1^H NMR, DOSY NMR, FT-IR, and CO_2_ adsorption (Figures S13–S17).

**Figure 2:**
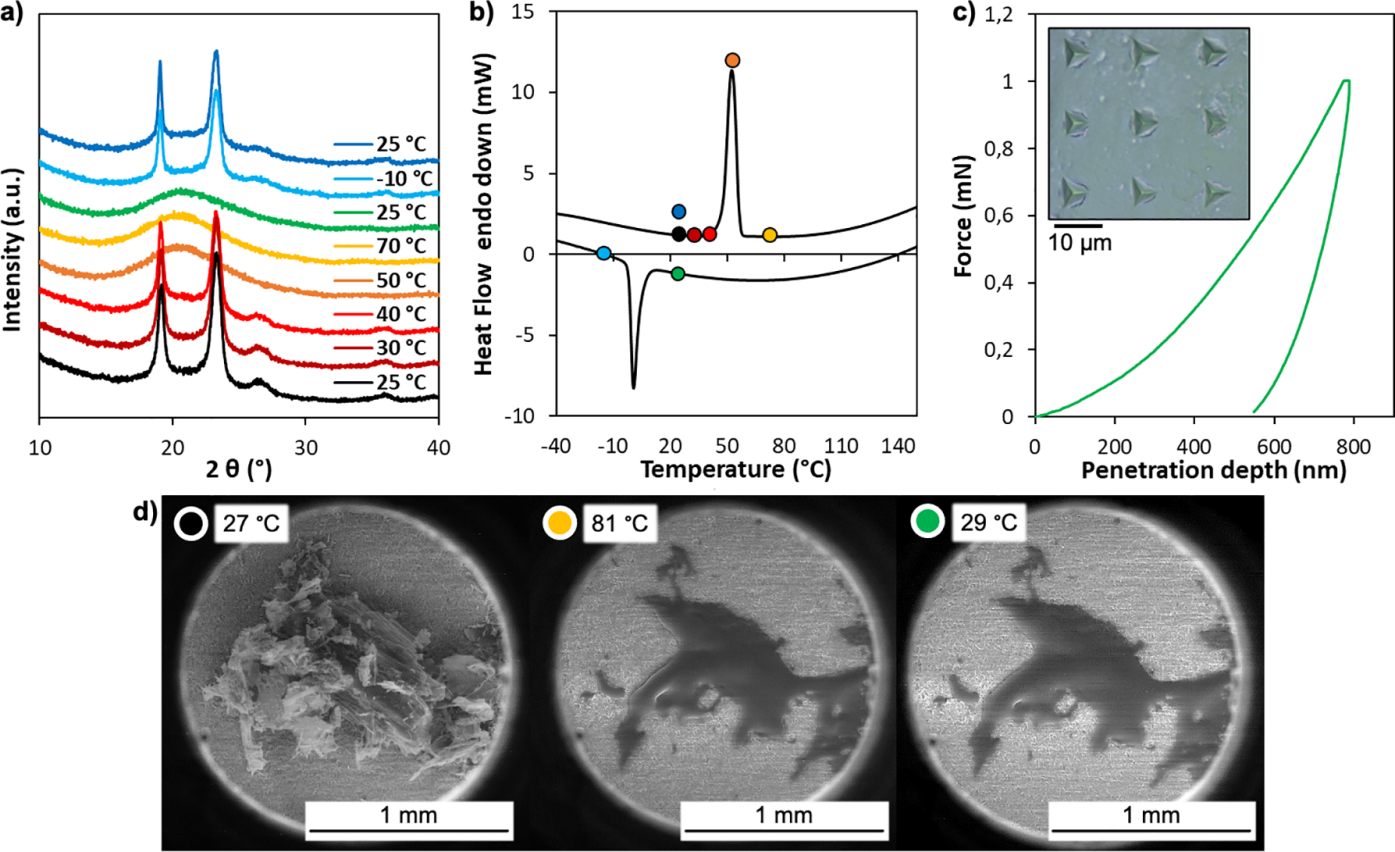
Temperature-dependent responses of BCN-93, as illustrated in a DSC curve (a) and in the corresponding VT-PXRD curve (b). (c) Representative load-displacement nanoindentation curve of the amorphous film (supercooled liquid) at room temperature. (inset) Micrograph revealing the indents. (d) VT-SEM images of as-synthesized BCN-93 (left), melted BCN-93 (middle), and the amorphous phase that results when melted BCN-93 is cooled down to 29 °C (right).

Once we had melted BCN-93 at 85 °C, we exploited its resulting liquid state to shape it into various forms and subsequently transformed the resultant samples into transparent amorphous films (Figure S18) by cooling them down to 25 °C (Figure 1b, bottom-right). The mechanical properties of thin amorphous films of at least 200 μm thickness (Figure S19) were assessed by nanoindentation measurements. These experiments revealed a reduced Young’s modulus (E*) of 2.3 GPa and a hardness (*H*) of 78 MPa (Figure 2c), which are above current MOP-based star polymers.^23^ Finally, the crystallinity of BCN-93 could be recovered by cooling it below 1 °C, without any loss in film shape or porosity (Figure 1, top-right; Figure S11). Moreover, upon grinding, the resultant crystalline films could be reconverted back into the initial powder phase (Figure 1b, top-left). The integrity of this ground powder was confirmed by ^1^H NMR, DOSY NMR, FT-IR, and CO_2_ adsorption (Figures S13, S14, and S16).

The impact of the melting-based shaping of BCN-93 on its porous properties was assessed through CO_2_ adsorption/desorption experiments run at 298 K. The corresponding isotherms revealed that the CO_2_ uptake of the as-made crystalline powder of BCN-93, of 0.2 mmol g^–1^ (11.3 mol of CO_2_/mol of MOP), was fully maintained after the melting-cooling process (Figure 3).

**Figure 3:**
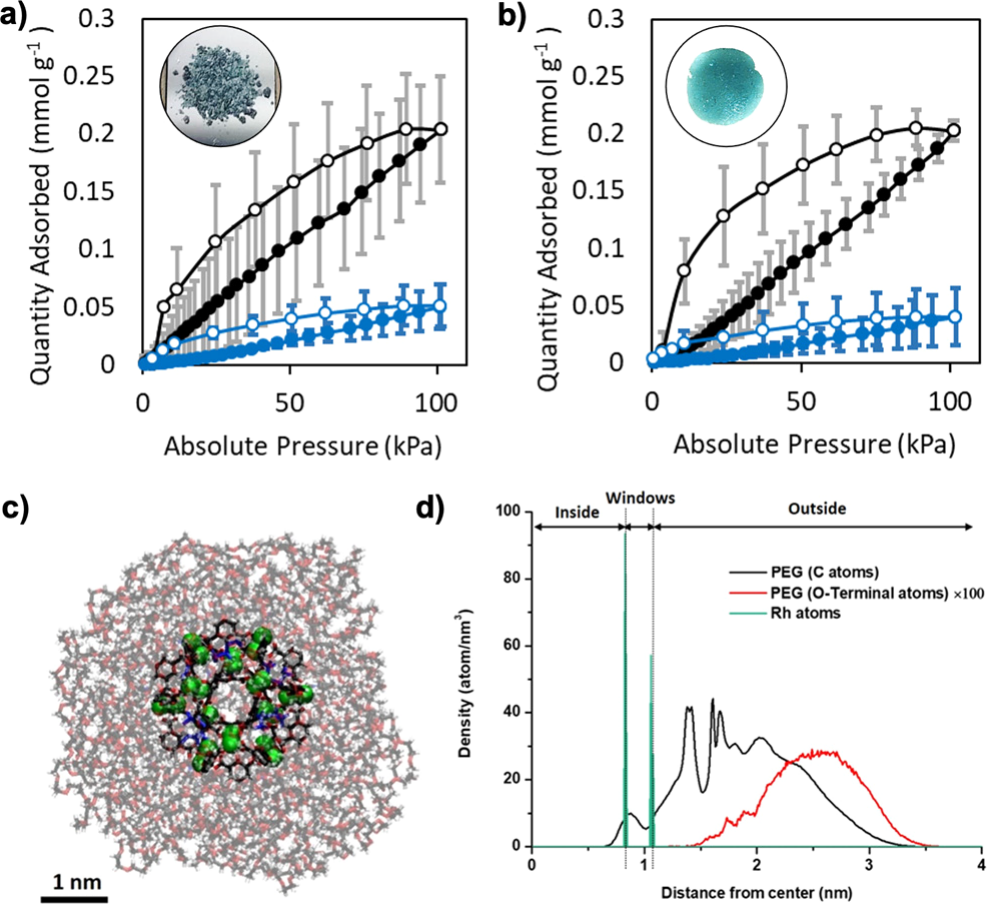
(a) CO_2_-sorption isotherms at 298 K of as-made BCN-93 (black) and pure PEG (blue). (b) CO_2_-sorption isotherms at 298 K of BCN-93 in its amorphous supercooled state (black) and pure PEG (blue). Error bars indicate standard deviation. (c) Snapshot of the equilibrium configuration of BCN-93 obtained by MD simulations at 298K. Color code: rhodium (green); carbon (black); hydrogen (white); oxygen (red). PEG atoms inside or at the windows of the MOP are highlighted in blue. (d) Radial density profiles of rhodium and carbon and terminal oxygen atoms of PEG chains obtained from MD simulations at 298K.

Reflecting on the high uptake of CO_2_ by BCN-93, we reasoned that the cavity inside BCN-93 is empty and remains accessible in both of the MOP’s physical forms, despite the presence of surface-bound PEG chains. However, previous reports have shown that free PEG chains have a high tendency to penetrate the cavities of porous materials (including cuboctahedral MOPs) and, consequently, block access to the MOP pores.^33−35^ In fact, our own data reveal this phenomenon: the CO_2_ uptake of BCN-93 (0.2 mmol of CO_2_ g^–1^) was indeed much higher than that of the physical mixture of free PEG and COOH-RhMOP (0.018 mmol of CO_2_ g^–1^, Figure S20). Thus, we reasoned that the surface-bound PEG chains in BCN-93 are much less likely to block pores in the MOP than are the free PEG chains, probably due to the mutual steric hindrance imparted by the high surface density of PEG chains in the former. Our hypothesis was supported by Molecular Dynamics simulations performed with NAMD^36^ and analyzed with VMD^37^ (see Supporting Information for details), which revealed that the PEG chains do not significantly penetrate the windows of the MOP at 25 °C, thereby leaving the cavity free from PEG chains both in solution and in dry conditions (Figure 3c,d and Figure S21). Furthermore, the empty cavity is accessible when exposed to guests such as CO_2_ (Figure S22). Conversely, when the unfunctionalized MOP core was simulated in the presence of free PEG chains, we observed a significant intrusion of PEG chains into the cavity of the MOP (Figure S23), consistent with our data and with the current literature. Thus, surface PEG functionalization of cuboctahedral MOPs enables coupling of the thermal behavior of the polymer to the persistent porosity of the MOP, without compromising the accessibility to the MOP cavity.

Finally, we aimed to use melted BCN-93 as a solvent in which to solubilize/disperse molecules and materials to yield mixed-matrix composite^38^ films prepared through the melting/cooling technique (Figure 4). Thus, we combined BCN-93 with one of four different additives at concentrations of 10% (w/w) or 20% (w/w): the MOF ZIF-8^39^ (Figure S24); the MOF UiO-66^40^ (Figure S24); the molecular cage OH-RhMOP;^41^ and the linear polymer poly(ethylene imine) (PEI). In each case, we followed the same synthetic strategy, which entailed combining the BCN-93 and the desired additive in methanol to yield a homogeneous solution/dispersion, which was subsequently lyophilized to afford a semicrystalline powder in which the two components were homogeneously distributed (Figures S25 and S26). Each mixture was melted at 85 °C (Figures S27 and S28), shaped, and finally cooled down to room temperature to produce self-standing, transparent, mixed-matrix composite films (Figures 4 and S29). Cross-sectional FE-SEM images and EDX mapping of the resulting films revealed a homogeneous distribution of particles of either MOF within the corresponding BCN-93 matrix (Figure 4 and Figures S30–S39), whereas XRPD showed that the crystalline structure of ZIF-8 and UiO-66 was maintained upon the melting/cooling cycle (Figure S26). As for the additives OH-RhMOP and PEI, FE-SEM images of the respective products revealed the formation of a homogeneous composite polymer film without any signs of segregation between BCN-93 and either additive (Figure 4, Figures S34–S37).

**Figure 4:**
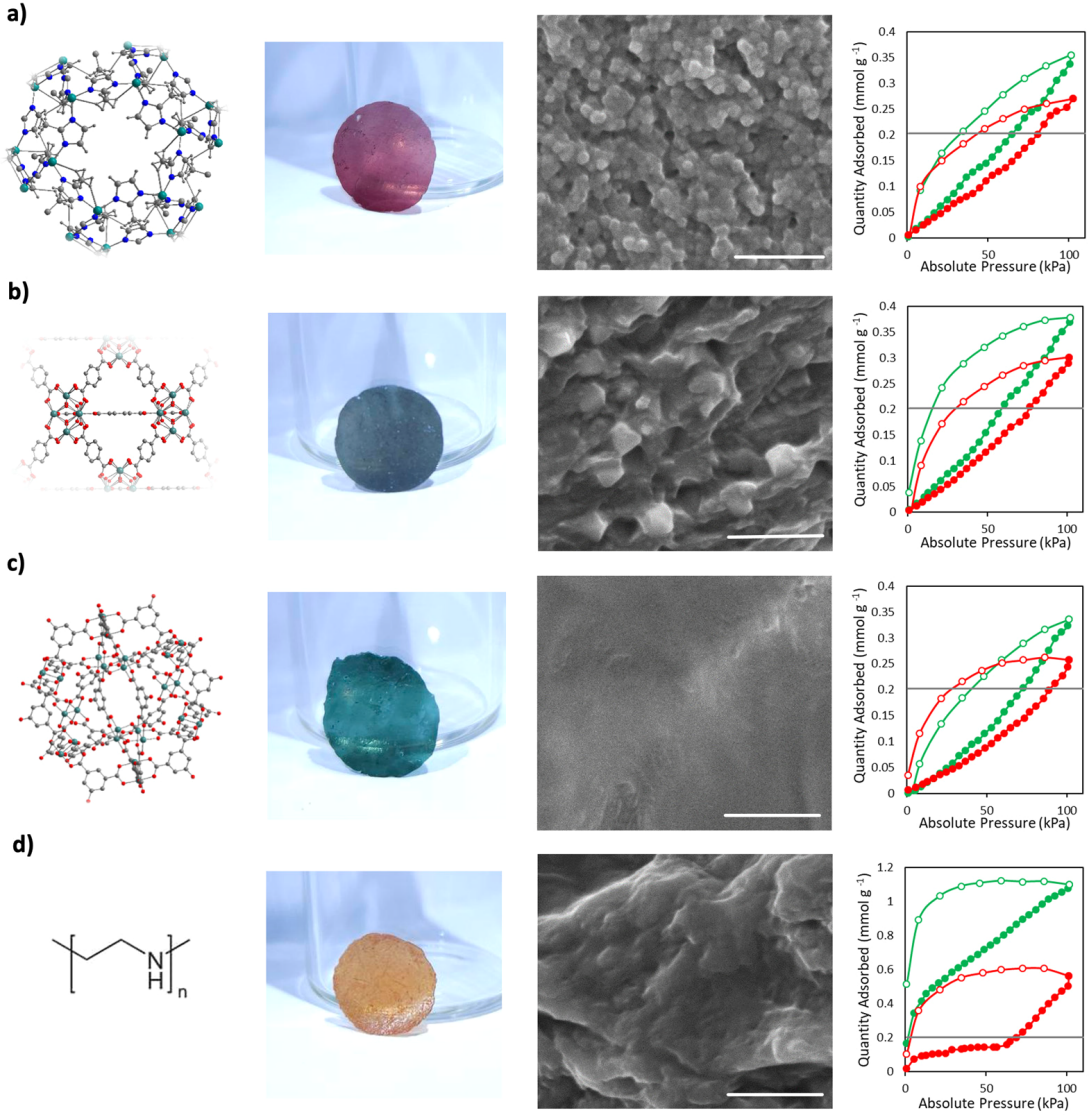
Mixed-matrix composite films containing (a) ZIF-8, (b) UiO-66, (c) OH-RhMOP, or (d) PEI. Left to right: representation of the crystal or molecular structure of the additive; Photograph of the self-standing translucent 20% (w/w) composite films; Cross-sectional FE-SEM images of the 20% (w/w) composite films (Scale bars: 1 μm); and CO_2_-sorption isotherms for the 10% (w/w; red) and 20% (w/w; green) composite films activated at 85 °C. Gray lines indicate the maximum uptake of BCN-93 film.

Interestingly, in all cases, the mixed-matrix composites exhibited greater CO_2_ uptake than did the parent (BCN-93) film (Figure 4). For the composites made from BCN-93 and either ZIF-8, UiO-66, or OH-RhMOP as an additive, the increase in CO_2_ uptake agreed with expected values, considering the individual contributions of each component (Figure S40). These results imply that the PEG chains of BCN-93 do not block the pores of MOFs or MOPs dispersed within the BCN-93 matrix, due to the bulky, ball-shaped conformation of the PEG chains on the surface of BCN-93. Conversely, when the same experiment was conducted using pure PEG as the solvent, the CO_2_ sorption was drastically lower, suggesting blockage of the inner cavity as well as penetration of the PEG chains into the pores of the MOFs and the MOP (Figure S41). Interestingly, the film made from BCN-93 and PEI exhibited markedly higher CO_2_ uptake (1.1 mmol g^–1^ at 20% PEI [w/w]) than did the parent film (0.2 mmol g^–1^), which is well-beyond the expected value, considering the individual contribution of each component. We theorized that this finding could be explained by a synergistic effect generated by the empty cavities within the polymer mixture. Specifically, we envisioned that the empty spaces would offer both higher solubility and greater diffusion pathways for CO_2_, thereby conferring superior accessibility of CO_2_ to the PEI-amino groups relative to those in a neat PEI film.

In summary, we described a new type of porous materials and related mixed-matrix composites that retained designed porosity upon melting-cooling cycles (Tables S1 and S2). We are confident that our results will inform the future synthesis of permanently porous materials that can be shaped into films, liquids, or glasses.

## Supporting Information Available

Detailed synthesis and methods, DLS analysis, UV/vis spectra, FT-IR spectra, TGA, NMR spectra, DSC curves, MALDI-TOF spectra, FE-SEM and EDX data and images, CO_2_ adsorption–desorption isotherms, optical transmission and reflectance spectra, and PXRD diffractograms (PDF)

## References

[ref1] Horike S.; Nagarkar S. S.; Ogawa T.; Kitagawa S. A. New Dimension for Coordination Polymers and Metal-Organic Frameworks: Towards Functional Glasses and Liquids. Angew. Chem., Int. Ed. 2020, 59, 6652–6664. 10.1002/anie.201911384. 31631497

[ref2] Wang M.; Zhao H.; Du B.; Lu X.; Ding S.; Hu X. Functions and Applications of Emerging Metal-Organic-Framework Liquids and Glasses. Chem. Commun. 2023, 59, 7126–7140. 10.1039/D3CC00834G. 37191098

[ref3] Zhang M.; Lai Y.; Li M.; Hong T.; Wang W.; Yu H.; Li L.; Zhou Q.; Ke Y.; Zhan X.; Zhu T.; Huang C.; Yin P. The Microscopic Structure-Property Relationship of Metal-Organic Polyhedron Nanocomposites. Angew. Chem., Int. Ed. 2019, 58, 17412–17417. 10.1002/anie.201909241. 31545541

[ref4] Liu D.; Li J.-C.; Ding S.; Lyu Z.; Feng S.; Tian H.; Huyan C.; Xu M.; Li T.; Du D.; Liu P.; Shao M.; Lin Y. 2D Single-Atom Catalyst with Optimized Iron Sites Produced by Thermal Melting of Metal-Organic Frameworks for Oxygen Reduction Reaction. Small Methods 2020, 4, 1900827 10.1002/smtd.201900827.

[ref5] Lin R.; Chai M.; Zhou Y.; Chen V.; Bennett T. D.; Hou J. Metal-Organic Framework Glass Composites. Chem. Soc. Rev. 2023, 52, 4149–4172. 10.1039/D2CS00315E. 37335141

[ref6] Egleston B. D.; Mroz A.; Jelfs K. E.; Greenaway R. L. Porous Liquids - the Future is Looking Emptier. Chem. Sci. 2022, 13, 5042–5054. 10.1039/D2SC00087C. 35655552 PMC9093153

[ref7] Xu W.; Hanikel N.; Lomachenko K. A.; Atzori C.; Lund A.; Lyu H.; Zhou Z.; Angell C. A.; Yaghi O. M. High-Porosity Metal-Organic Framework Glasses. Angew. Chem., Int. Ed. 2023, 62, e202300003 10.1002/anie.202300003. PMC10503658 36791229

[ref8] Bumstead A. M.; Castillo-Blas C.; Pakamorė I.; Thorne M. F.; Sapnik A. F.; Chester A. M.; Robertson G.; Irving D. J. M.; Chater P. A.; Keen D. A.; Forgan R. S.; Bennett T. D. Formation of a Meltable Purinate Metal-Organic Framework and its Glass Analogue. Chem. Commun. 2023, 59, 732–735. 10.1039/D2CC05314D. 36541403

[ref9] Brand M. C.; Greenwell F.; Clowes R.; Egleston B. D.; Kai A.; Cooper A. I.; Bennett T. D.; Greenaway R. L. Melt-Quenched Porous Organic Cage Glasses. J. Mater. Chem. A 2021, 9, 19807–19816. 10.1039/D1TA01906F.

[ref10] Bavykina A.; Cadiau A.; Gascon J. Porous Liquids based on Porous Cages, Metal Organic Frameworks and Metal Organic Polyhedra. Coord. Chem. Rev. 2019, 386, 85–95. 10.1016/j.ccr.2019.01.015.

[ref11] Nozari V.; Calahoo C.; Tuffnell J. M.; Keen D. A.; Bennett T. D.; Wondraczek L. Ionic Liquid Facilitated Melting of the Metal-Organic Framework ZIF-8. Nat. Commun. 2021, 12, 5703 10.1038/s41467-021-25970-0. 34588462 PMC8481281

[ref12] Healy C.; Patil K. M.; Wilson B. H.; Hermanspahn L.; Harvey-Reid N. C.; Howard B. I.; Kleinjan C.; Kolien J.; Payet F.; Telfer S. G.; Kruger P. E.; Bennett T. D. The Thermal Stability of Metal-Organic Frameworks. Coord. Chem. Rev. 2020, 419, 213388 10.1016/j.ccr.2020.213388.

[ref13] Widmer R. N.; Lampronti G. I.; Anzellini S.; Gaillac R.; Farsang S.; Zhou C.; Belenguer A. M.; Wilson C. W.; Palmer H.; Kleppe A. K.; Wharmby M. T.; Yu X.; Cohen S. M.; Telfer S. G.; Redfern S. A. T.; Coudert F.-X.; MacLeod S. G.; Bennett T. D. Pressure Promoted Low-Temperature Melting of Metal-Organic Frameworks. Nat. Mater. 2019, 18, 370–376. 10.1038/s41563-019-0317-4. 30886398

[ref14] Longley L.; Collins S. M.; Li S.; Smales G. J.; Erucar I.; Qiao A.; Hou J.; Doherty C. M.; Thornton A. W.; Hill A. J.; Yu X.; Terrill N. J.; Smith A. J.; Cohen S. M.; Midgley P. A.; Keen D. A.; Telfer S. G.; Bennett T. D. Flux Melting of Metal-Organic Frameworks. Chem. Sci. 2019, 10, 3592–3601. 10.1039/C8SC04044C. 30996951 PMC6430010

[ref15] Bumstead A. M.; Ríos Gómez M. L.; Thorne M. F.; Sapnik A. F.; Longley L.; Tuffnell J. M.; Keeble D. S.; Keen D. A.; Bennett T. D. Investigating the Melting Behaviour of Polymorphic Zeolitic Imidazolate Frameworks. CrystEngComm 2020, 22, 3627–3637. 10.1039/D0CE00408A.

[ref16] Bennett T. D.; Yue Y.; Li P.; Qiao A.; Tao H.; Greaves N. G.; Richards T.; Lampronti G. I.; Redfern S. A. T.; Blanc F.; Farha O. K.; Hupp J. T.; Cheetham A. K.; Keen D. A. Melt-Quenched Glasses of Metal-Organic Frameworks. J. Am. Chem. Soc. 2016, 138, 3484–3492. 10.1021/jacs.5b13220. 26885940

[ref17] To T.; Sørensen S. S.; Stepniewska M.; Qiao A.; Jensen L. R.; Bauchy M.; Yue Y.; Smedskjaer M. M. Fracture Toughness of a Metal-Organic Framework Glass. Nat. Commun. 2020, 11, 2593 10.1038/s41467-020-16382-7. 32444664 PMC7244719

[ref18] Liu M.; McGillicuddy R. D.; Vuong H.; Tao S.; Slavney A. H.; Gonzalez M. I.; Billinge S. J. L.; Mason J. A. Network-Forming Liquids from Metal-Bis(acetamide) Frameworks with Low Melting Temperatures. J. Am. Chem. Soc. 2021, 143, 2801–2811. 10.1021/jacs.0c11718. 33570911

[ref19] Furukawa S.; Horike N.; Kondo M.; Hijikata Y.; Carné-Sánchez A.; Larpent P.; Louvain N.; Diring S.; Sato H.; Matsuda R.; Kawano R.; Kitagawa S. Rhodium-Organic Cuboctahedra as Porous Solids with Strong Binding Sites. Inorg. Chem. 2016, 55, 10843–10846. 10.1021/acs.inorgchem.6b02091. 27748586

[ref20] Albalad J.; Hernández-López L.; Carné-Sánchez A.; Maspoch D. Surface Chemistry of Metal-Organic Polyhedra. Chem. Commun. 2022, 58, 2443–2454. 10.1039/D1CC07034G. 35103260

[ref21] Paberit R.; Rilby E.; Göhl J.; Swenson J.; Refaa Z.; Johansson P.; Jansson H. Cycling Stability of Poly(ethylene glycol) of Six Molecular Weights: Influence of Thermal Conditions for Energy Applications. ACS Appl. Energy Mater. 2020, 3, 10578–10589. 10.1021/acsaem.0c01621.

[ref22] Ma L.; Haynes C. J. E.; Grommet A. B.; Walczak A.; Parkins C. C.; Doherty C. M.; Longley L.; Tron A.; Stefankiewicz A. R.; Bennett T. D.; Nitschke J. R. Coordination Cages as Permanently Porous Ionic Liquids. Nat. Chem. 2020, 12, 270–275. 10.1038/s41557-020-0419-2. 32042136

[ref23] Nagarkar S. S.; Tsujimoto M.; Kitagawa S.; Hosono N.; Horike S. Modular Self-Assembly and Dynamics in Coordination Star Polymer Glasses: New Media for Ion Transport. Chem. Mater. 2018, 30, 8555–8561. 10.1021/acs.chemmater.8b03481.

[ref24] He C.; Zou Y.-H.; Si D.-H.; Chen Z.-A.; Liu T.-F.; Cao R.; Huang Y.-B. A Porous Metal-Organic Cage Liquid for Sustainable CO2 Conversion Reactions. Nat. Commun. 2023, 14, 3317 10.1038/s41467-023-39089-x. 37286561 PMC10247695

[ref25] Cheng S. Z. D.; Barley J. S.; Giusti P. A. Spherulite Formation in Poly(ethylene oxide) Mixtures. Polymer 1990, 31, 845–849. 10.1016/0032-3861(90)90045-Z.

[ref26] Huang L.; Nishinari K. Interaction Between Ppoly(ethylene glycol) and Water as Studied by Differential Scanning Calorimetry. J. Polym. Sci. Part B. Polym. Phys. 2001, 39, 496–506. 10.1002/1099-0488(20010301)39:5<496::AID-POLB1023>3.0.CO;2-H.

[ref27] Samuel A. Z.; Umapathy S. Energy Funneling and Macromolecular Conformational Dynamics: a 2D Raman Correlation Study of PEG Melting. Polym. J. 2014, 46, 330–336. 10.1038/pj.2014.10.

[ref28] Matsuura H.; Fukuhara K. Vibrational Spectroscopic Studies of Conformation of Poly(oxyethylene). II. Conformation-Spectrum Correlations. J. Polym. Sci. Part B. Polym. Phys. 1986, 24, 1383–1400. 10.1002/polb.1986.090240702.

[ref29] Kwon O. H.; Ortalan V.; Zewail A. H. Macromolecular Structural Dynamics Visualized by Pulsed Dose Control in 4D Electron Microscopy. Proc. Natl. Acad. Sci. USA 2011, 108, 6026–6031. 10.1073/pnas.1103109108. 21444766 PMC3076862

[ref30] Albalad J.; Carné-Sánchez A.; Grancha T.; Hernández-López L.; Maspoch D. Protection Strategies for Directionally-Controlled Synthesis of Previously Inaccessible Metal-Organic Polyhedra (MOPs): the Cases of Carboxylate- and Amino-Functionalised Rh(II)-MOPs. Chem. Commun. 2019, 55, 12785–12788. 10.1039/C9CC07083D. 31591620

[ref31] Li R.; Wu Y.; Bai Z.; Guo J.; Chen X. Effect of Molecular Weight of Polyethylene Glycol on Crystallization Behaviors, Thermal Properties and Tensile Performance of Polylactic Acid Stereocomplexes. RSC Adv. 2020, 10, 42120–42127. 10.1039/D0RA08699A. 35516761 PMC9057859

[ref32] Napolitano S.; Glynos E.; Tito N. B. Glass Transition of Polymers in Bulk, Confined Geometries, and Near Interfaces. Rep. Prog. Phys. 2017, 80, 036602 10.1088/1361-6633/aa5284. 28134134

[ref33] Tsang M. Y.; Convery J. P.; Lai B.; Cahir J.; Erbay Y.; Rooney D.; Murrer B.; James S. L. Porous Liquids as Solvents for the Economical Separation of Carbon Dioxide from Methane. MaterialsToday 2022, 60, 9–16. 10.1016/j.mattod.2022.09.004.

[ref34] Hung H.-L.; Iizuka T.; Deng X.; Lyu Q.; Hsu C.-H.; Oe N.; Lin L.-C.; Hosono N.; Kang D.-Y. Engineering Gas Separation Property of Metal-Organic Framework Membranes via Polymer Insertion. Sep. Purif. Technol. 2023, 310, 123115 10.1016/j.seppur.2023.123115.

[ref35] Nishijima A.; Kametani Y.; Uemura T. Reciprocal Regulation between MOFs and Polymers. Coord. Chem. Rev. 2022, 466, 214601 10.1016/j.ccr.2022.214601.

[ref36] Phillips J. C.; Braun R.; Wang W.; Gumbart J.; Tajkhorshid E.; Villa E.; Chipot C.; Skeel R. D.; Kale L.; Schulten K. Scalable Molecular Dynamics with NAMD. J. Comput. Chem. 2005, 26, 1781–1802. 10.1002/jcc.20289. 16222654 PMC2486339

[ref37] Humphrey W.; Dalke A.; Schulten K. VMD - Visual Molecular Dynamics. J. Mol. Graphics. 1996, 14, 33–38. 10.1016/0263-7855(96)00018-5. 8744570

[ref38] Denny M. S.; Moreton J. C.; Benz L.; Cohen S. M. Metal-Organic Frameworks for Membrane-Based Separations. Nat. Rev. Mater. 2016, 1, 1–17. 10.1038/natrevmats.2016.78.

[ref39] Park K. S.; Ni Z.; Côte A. P.; Choi J. Y.; Huang R.; Uribe-Romo F. J.; Chae H. K.; O’Keeffe M.; Yaghi O. M. Exceptional Chemical and Thermal Stability of Zeolitic Imidazolate frameworks. Proc. Natl. Acad. Sci. U.S.A. 2006, 103, 10186–10191. 10.1073/pnas.0602439103. 16798880 PMC1502432

[ref40] Cavka J. H.; Jakobsen S.; Olsbye U.; Guillou N.; Lamberti C.; Bordiga S.; Lillerud K. P. A. New Zirconium Inorganic Building Brick Forming Metal Organic Frameworks with Exceptional Stability. J. Am. Chem. Soc. 2008, 130, 13850–13851. 10.1021/ja8057953. 18817383

[ref41] Carné-Sánchez A.; Albalad J.; Grancha T.; Imaz I.; Juanhuix J.; Larpent P.; Furukawa S.; Maspoch D. Postsynthetic Covalent and Coordination Functionalization of Rhodium(II)-Based Metal-Organic Polyhedra. J. Am. Chem. Soc. 2019, 141, 4094–4102. 10.1021/jacs.8b13593. 30721045

