# Supplementary material for: Porous and Meltable Metal–Organic Polyhedra for the Generation and Shaping of Porous Mixed-Matrix Composites
Source: J Am Chem Soc. 2024 Mar 11;146(11):7159–64. doi: 10.1021/jacs.4c00407 (PMC10958503; doi:10.1021/jacs.4c00407)
Supplement: Supplementary file 1 — ja4c00407_si_001.pdf [file ja4c00407_si_001.pdf]

Supporting information for:

Porous and Meltable Metal-Organic Polyhedra for the Generation and Shaping of Porous Mixed-Matrix Composites

Cornelia von Baeckmann,[a,b] Jordi Martínez-Esaín,[a] José A. Suárez del Pino,[a,b] Lingxin Meng, [a,b] Joan Garcia-Masferrer,[c] Jordi Faraudo,[c] Jordi Sort,[d,e] Arnau Carné-Sánchez[b,a]* and Daniel Maspoch[a,b,e]*

[a.] Catalan Institute of Nanoscience and Nanotechnology (ICN2), CSIC, and The Barcelona Institute of Science and Technology, Campus UAB, 08193 Bellaterra, Spain

[b.] Departament de Química, Facultat de Ciències, Universitat Autònoma de Barcelona, 08193 Bellaterra, Spain

[c.] Institut de Ciència de Materials de Barcelona (ICMAB-CSIC), 08193 Bellaterra, Spain

[d.] Departament de Física, Universitat Autònoma de Barcelona, 08193 Bellaterra, Spain

[e.] ICREA, Pg. Lluís Companys 23, 08010 Barcelona, Spain

Table of contents:

S1. Materials and methods:

S1.1 Materials and characterization………………………………………………… S3

S1.2. Synthetic procedures………………………………………………………….. S6

S1.3. Molecular dynamics simulations methods……………………………...…… S9

S2. Characterization of BCN-23…………………………………………….………. S11

S3. Characterization amorphous composite films………………………………… S34

S4. References……………………………………………………………………..……… S54

S1. Materials and methods

S1.1 Materials and characterization

1,3,5-Benzenetricarbonyl trichloride, tetrabutylammonium fluoride solution (1 M in THF), linear polyethylene imine (MW 2000), $ZrCl_4$, 1,4-benzendicarboxylic acid, $Zn(NO_3)_2 \cdot 6H_2O$, 2-methylimidazole, acetic acid, hydroxybenzotriazole (HOBt), N,N-diisopropylethylamine (DIPEA), mPEG-OCH_3 (MW 2000) and pyridine were obtained from Sigma-Aldrich in high purity grade. 2-(Trimethylsilyl)ethanol (TMS-OH) and hexafluorophosphate benzotriazole tetramethyl uronium (HBTU) were purchased from TCI. DMSO-d_6, MeOD, D_2O and $CDCl_3$ were obtained from euroisotop, rhodium(II) acetate dimer and deuterium chloride (DCl, 20 wt% in D_2O) from Acros Organics. Dichloromethane (DCM), diethyl ether (Et_2O), tetrahydrofuran (THF), N,N-diemethylacetamide (DMA), dimethylformamide (DMF), chloroform ($CHCl_3$) and methanol (MeOH) were used without any further purification from Fisher Scientific. NH_2-PEG-OCH_3 (MW 2000) was purchased from Rapp Polymers. All reagents were used as obtained by the suppliers, unless it is stated otherwise.

Nuclear magnetic resonance (NMR). All ^1H NMR spectra were recorded using a Bruker Avance NEO 300 NMR spectrometer at 25 °C. Chemical shifts (δ) are reported in ppm. The resonance of residual MeOH for MeOD (4.87 ppm (OH) and 3.31 ppm (quintuplet)) and DMSO for DMSO-d_6 (2.5 ppm) is used as internal reference. ^1H NMR splitting patterns were indicated as singlet (s), doublet (d) and triplet (t).

Matrix-assisted laser desorption/ionization-time of flight (MALDI-TOF) mass spectrometry (MS) measurements were performed using a 4800 Plus MALDI TOF/TOF (ABSCIEX – 2010) operating in positive-ionization mode using trans-2-[3-(4-tert-butylphenyl)-2-methyl-2-propenylidene]malononitrile (DCTB) as ionization matrix.

Scanning Electron Microscopy (SEM) and variable temperature SEM (VT-SEM) was performed in a SEM Quanta 650 FEM.

Ultraviolet-visible (UV−Vis) spectra were measured using an Thermo Scientific NanoDrop 2000 at room temperature (*ca*. 25 °C).

Powder X-ray diffraction (powder-XRD) diagrams were collected on a Panalytical X'pert diffractometer with monochromatic Cu-K_α radiation (λ_{Cu} = 1.5406 Å).

The **variable temperature (VT-XRD)** measurements were performed in a TTK600 Low-Temperature Chamber from Anton Paar which was mounted inside an X'pert Pro MPD Malvern-Panalytical Diffractometer. This temperature chamber allows to make studies from -20 ºC to 600 ºC using different atmospheres (vacuum, inert gases or air). In this experiment, the samples were mounted in a Zero-Background Si Holder for reflection geometry. The sample was heated up until 110 ºC under air. Different temperature steps were performed:
1st Process → From RT to 40 ºC,
increment of temperature =10 ºC/min, and a D-well= 5min to stabilize temperature in each step.
2nd Process → From 40 ºC to 60 ºC,
increment of temperature =5 ºC/min, and a D-well= 5min to stabilize temperature in each step.
3rd Process → From 60 ºC to 110 ºC,
increment of temperature =10 ºC/min, and a D-well= 5min to stabilize temperature in each step.
4th Process → From 110 ºC to 40 ºC,
increment of temperature =10 ºC/min, and a D-well= 5min to stabilize temperature in each step.
5th Process → From 40 ºC to -10 ºC,
increment of temperature =5 ºC/min, and a D-well= 5min to stabilize temperature in each step.
6th Process → From -10 ºC to RT, to compare the measurement with the first scan.
The scans were measured from 10º - 40º, with a step size of 0.01º and a counting time of 50 s.

CO_2 adsorption measurements were performed at 298 K using an S4 ASAP 2020 (Micromeritics). Prior to the measurements, the samples were activated either at RT or at 85 ºC under vacuum. The standard deviation was calculated from 3 independent samples.

Z-potential measurements were carried out using a Malvern Zetasizer Nano ZS. Prior to zeta-potential measurements, a standard solution with a zeta-potential of −42 ± 6 mV was measured to ensure correct calibration.

Thermogravimetric analysis (TGA) was performed on a Netzsch STA 449F1 instrument from 23 to 600 ºC using a heating rate of 10 K / min.

Differential scanning calorimetry (DSC) measurements were carried out using a Perkin Elmer DSC 8000 under Nitrogen atmosphere (20 mL / min) and a heating rate of 10 ºC / min. Two full heating and one full cooling cycle were performed for each sample from -60 ºC to 200 ºC.

Nanoindentation was carried out on a NHT2 (Anton Paar) Berkovich nano-Hardness tester at room temperature, applying a maximum load of 1 mN and a loading and unloading rate of 2 mN min^{-1}. The thermal drift during nanoindentation was kept below 0.05 nm / s. The acquisition rate was 10 Hz and the hardness, elastic and plastic deformation energies and reduced Young's Modulus were calculated as an average from a total of 100 measurements.

Topography was performed using a Leica Map Premium 6.2.6409 in order to determine the sample thickness.

S1.2 Synthetic procedures

COOH-RhMOP was synthesized according to previously described methodology. [1]

BCN-93 was synthesized from COOH-RhMOP by functionalizing its surface with 24 NH_2-mPEG (2 kDa) chains through amide coupling. For the activation of the carboxylic acid of the COOH-RhMOP and subsequent amide coupling, HOBt, HBTU and DIPEA were used. The reaction proceeded as follows. First, 60 mg of COOH-RhMOP (0.008 mmol) were dissolved in 0.5 mL DMF followed by the addition of 1 ml DMF solution containing HOBt (24 eq., 0.192 mmol) and HBTU (29 eq., 0.232 mmol). After stirring for at least 10 minutes, DIPEA (29 eq., 0.232 mmol) was added, which induced the instantaneous precipitation of the active ester-MOP. After leaving the reaction stirring for 30 minutes at room temperature, the 2 kDa NH_2-mPEG (29 eq., 0.232 mmol in 1mL DMF) was added. The reaction was left stirring at room temperature overnight. After this reaction time, the product got solubilized in the reaction medium. The product was isolated by precipitating it with Et_2O. The product was recovered through centrifugation and subsequently washed with Et_2O. The red crude solid was dissolved in 10 mL water and extracted with a DCM/Methanol (1:1) mixture. The DCM phase was washed several times with water/methanol (1:1) mixtures until it changed colour from red to green. This change in colour is indicative of the complete removal of the coupling agents. Then, the organic phase was dried to obtain a green solid. In order to remove uncoupled polymer, the dried sample was re-dissolved in water and filtered by centrifugal filters with a cut-off of 50.000 g/mol. The BCN-93 was retained whereas the free PEG went through the filter. This filtering process was repeated several times, with fresh water (approx. 100 mL). The concentrated green solution was finally lyophilized in order to remove remaining water. The obtained product was BCN-93 in its semicrystalline, powder form.

Synthesis of amorphous and shaped films made of BCN-93

To melt the as-made semicrystalline BCN-93 powder, the sample was placed in a pre-heated oven at 85 °C oven for 10 minutes. Then, the melted sample was shaped using a spatula and non-adhesive tape and left at 25 °C overnight before it was removed from the support-tape to obtain an amorphous self-standing film.

Synthesis of BCN-93 semicrystalline shaped films made of BCN-93

To obtain semicrystalline films, the amorphous shaped films were frozen at -85 ºC and lyophilized overnight. The shape of the film was retained through this process.

Acid Digestions

Approx. 5 mg of the BCN-93 were dissolved in a mixture of 400 µL d6-DMSO and 20 µL DCl (20 wt% in D_2O). The mixture was heated at 100 °C for 1 hour to achieve complete digestion of the sample. To break the formed amide, the sample was digested using 400 µL DCl (20 wt% in D_2O) at 100 °C overnight.

Composite films

UiO-66 was synthesized according to a previously described methodology. [2] In a typical synthesis, $ZrCl_4$ (70 mg) and 1,4-benzendicarboxylic acid (BDC; 50 mg) were dissolved in 10 ml of 1.5 M acetic acid in DMF and transferred to a scintillation vial, which was placed in a preheated oven at 120 °C for 12 hours. The colloidal crystals of average size of 203 nm were then recovered by centrifugation (at 9000 rpm for 10 min in a 50 ml Falcon tube) and washed three times with DMF and three times with methanol. The collected particles were finally redispersed in methanol for storage. To calculate the concentration of the UiO-66 dispersion in methanol, an aliquot of the stock solution was dried at 120 °C. Then, a suspension containing 3 or 6 mg of UiO-66 particles were added to a vial containing 27 or 24 mg of BCN-93, respectively, to create composites with 10 or 20 wt% of UiO-66 in BCN-93. Each mixture was subjected to vortex and ultrasonic bath treatment until BCN-93 was fully solubilized. The green methanol suspensions were dried at room temperature and finally lyophilized. The resulting powder was processed into an amorphous shaped film as indicated above for pure BCN-93 amorphous films.

ZIF-8 was synthesized according to a previously described methodology.[3] Briefly, $Zn(NO_3)_2 \cdot 6H_2O$ (2.9 g, 0.0097 mol) and 2-methylimidazole (6.5 g, 0.079 mol) were each dissolved in methanol (100 ml). The two solutions were then mixed rapidly and stirred at 850 rpm for 1 hour at room temperature. The colloidal crystals with an average size of 112 nm were then recovered by centrifugation (at 8000 rpm for 25 min in 50 ml Falcon tubes) and washed four times with methanol. The collected particles were finally redispersed in methanol for storage. To calculate the concentration of the ZIF-8 solution in methanol, an aliquot of the stock solution was dried at 120 °C. Then, a suspension containing 3 or 6 mg of ZIF-8 nanoparticles were added to a vial containing 27 or 24 mg of BCN-93, respectively, to create composites with 10 or 20 wt% of ZIF-8 in BCN-93. Each mixture was subjected to vortex and ultrasonic bath treatment until BCN-93 was fully solubilized. The methanol suspensions were dried at room temperature and finally lyophilized. The resulting powder was processed into an amorphous shaped film as indicated above for pure BCN-93 amorphous films.

OH-RhMOP was synthesized according to a previously described methodology.[4] OH-RhMOP and BCN-93 were mixed at an OH-RhMOP/BCN-93 weight ratio of 0.1 (10 wt%) and 0.2 (20 wt%) in methanol. Each resulting solution was dried at room temperature and lyophilized. Each resulting powder was processed into an amorphous shaped film as indicated above for pure BCN-93 amorphous films.

PEI and BCN-93 were mixed at a PEI/BCN-93 weight ratio of 0.1 (10 wt%) and 0.2 (20 wt%) in methanol. Each resulting solution was dried at room temperature and lyophilized. Each resulting powder was processed into an amorphous shaped film as indicated above for pure BCN-93 amorphous films.

Physical Mixture

The physical mixture of mPEG-OCH_3 and COOH-RhMOP was prepared by considering the same mass ratio of PEG:MOP than in BCN-93. Briefly, 87 mg of mPEG-OCH_3 and 13 mg of COOH-MOP were solubilized in water. This solution was lyophilized to obtain a semicrystalline powder containing free PEG and COOH-RhMOP.

S1.3. Molecular dynamics simulations methods

The details of all simulations employed in this study are summarized in Table S1. All simulations were made using the NAMD program (version 2.14).[5] All chemical species were described with full atomistic detail. The force field employed in the simulations was the CHARMM General Force Field (CgenFF).[6,7] For PEG and CO_2, we employed standard CHARMM parameters. Water was modelled using the TIP3P model as usual in CHARMM. The values of the parameters employed for the linker of the MOPs were the same as in our previous works[8,9] generated by CHARMM General Force Field (CGenFF) program. For the Rh atoms, we used values fitted from DFT calculations (Rmin= 1.3575 Å and ε= -1.973 kcal/mol) as in our previous works.[8,9] In all our simulations, we fixed the atomic positions of the Rh atoms and of the O atoms directly coordinated to Rh.

In the MD simulations, the Newton equations of motion were solved using a time step of 2 fs. Electrostatic interactions were computed using the PME method with the standard settings in NAMD (1 Å resolution, updated each 2 time steps). Lennard-Jones interactions were truncated at 1.2 nm employing a switching function starting at 1.0 nm. Periodic boundary conditions were employed in all directions. In all simulations, we employed the Langevin thermostat at 298K as implemented in NAMD with the standard relaxation time of 1 ps. In all the simulations that contain water as solvent, the pressure was kept constant at 1 atm using the Nosé-Hoover-Langevin piston barostat (oscillation period of 100 fs and decay time of 50 fs). In all cases, before running the actual MD simulations, we performed an energy minimization of the initial configurations using NAMD to solve possible bad contacts between atoms. The energy minimization was followed by an equilibration run to thermalize the system and adjust the size of the simulation box in the case of NpT simulations. After the equilibration run, production runs were performed with different durations depending on the dynamics of each particular system (see results). The results of the simulations were analyzed using custom python scripts with MDanalysis.[11] All snapshots were made with VMD.[10]

The initial structures for the Rh-MOP used in simulation 1 and 2 were the same as in our previous works [8,9] and were generated using the Molefacture plugin version 2.0 included in VMD [10] version 1.9.3. The initial coordinates of the BCN-93 MOP (simulations 3-5) were generated by functionalizing a Rh-MOP structure with appropriate PEG fragments (45 monomers) using the Molefacture plugin. The fragment files in a format suitable for Molefacture, the resulting coordinates and structures and the forcefield parameters are freely available at our GitHub repository.[11] Simulation 4, corresponding to the evacuated BCN-93, was achieved by removing all water molecules from simulation 3 replicating the experimental procedure by which hydrated BCN-93 is dried through lyophilization. The so obtained simulation was thermalized and equilibrated during additional 160 ns without water. The minimized structure obtained in simulation 4 was used as starting point for simulation 5. Thus, in simulation 5 we have considered the addition of CO_2 to the structure obtained in simulation 4. To this end, we have added CO_2 molecules from a pre-equilibrated gas box to the last configuration of simulation 4 using the "nonstandard" solvent option of the "solvate" plugin of the VMD program. All CO_2 molecules

initially placed by VMD inside the cavity of the MOP were removed. Therefore, at t=0, CO_2 was found only outside the MOP. As shown in Figure S19, CO_2 diffuses across the structure and fills the cavity of the MOP, with an average of 30.8 CO_2 molecules inside the cavity.

System	Composition	Num atoms	Ensemble	Simulation box size
1	H-RhMOP / 3,457 water molecules	10,779	NpT	106.56 nm^3
2	H-RhMOP / 10 PEG chains / 30,160 water molecules	94,198	NpT	929.71 nm^3
3	BCN-93 /184,887 water molecules	562,965	NpT	5,548.05 nm^3
4	BCN-93	8,304	NVT	5,548.05 nm^3
5	BCN-93 / 20,237 CO_2 molecules	69,015	NVT	3,375 nm^3

S2. Characterization of BCN-93

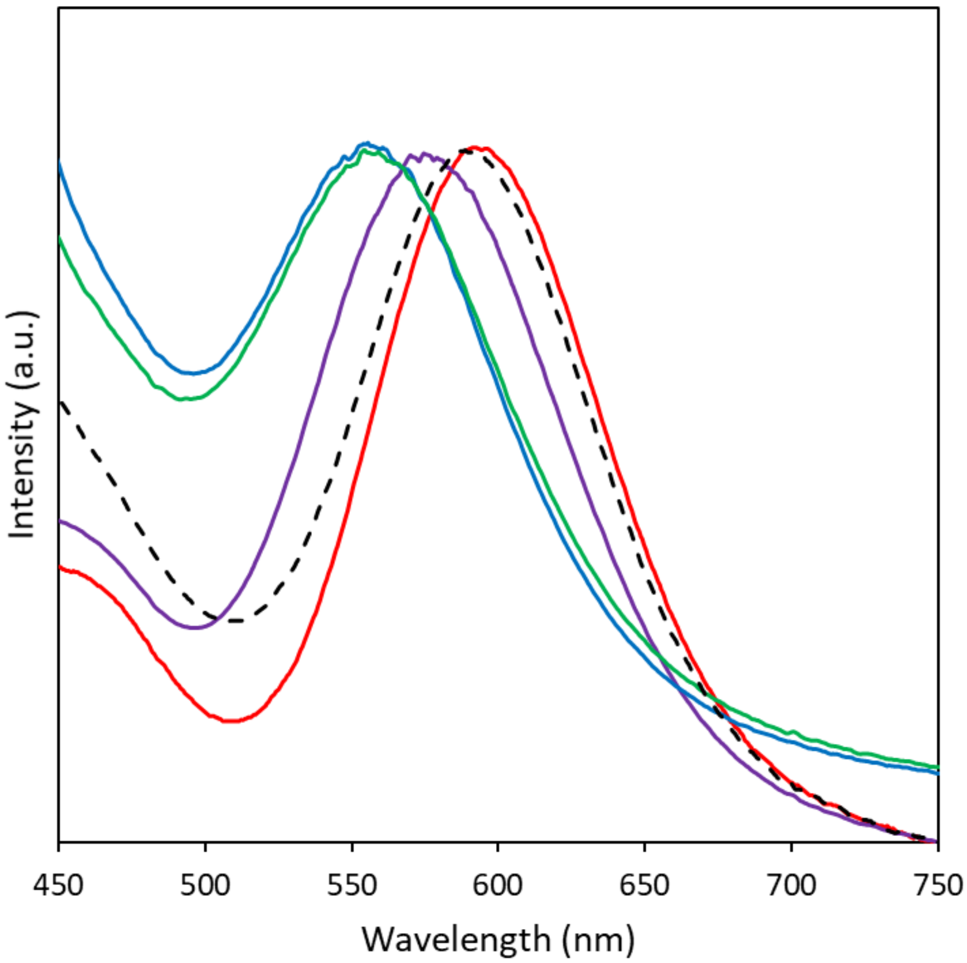

Figure S1. UV/Vis monitoring of the amide bond formation on the surface of COOH-RhMOP (red). Upon addition of the coupling agents HOBt/HBTU (purple) and DIPEA (blue), the λ_{max} ascribed to the Rh-Rh paddlewheel shifts from 599 to 557 nm due to the coordination of these coupling agents to the axial site of the Rh(II) paddlewheel. After the work-up, the initial value of λ_{max} is recovered showing the integrity of the Rh(II) paddlewheel and the complete removal of the coupling agents.

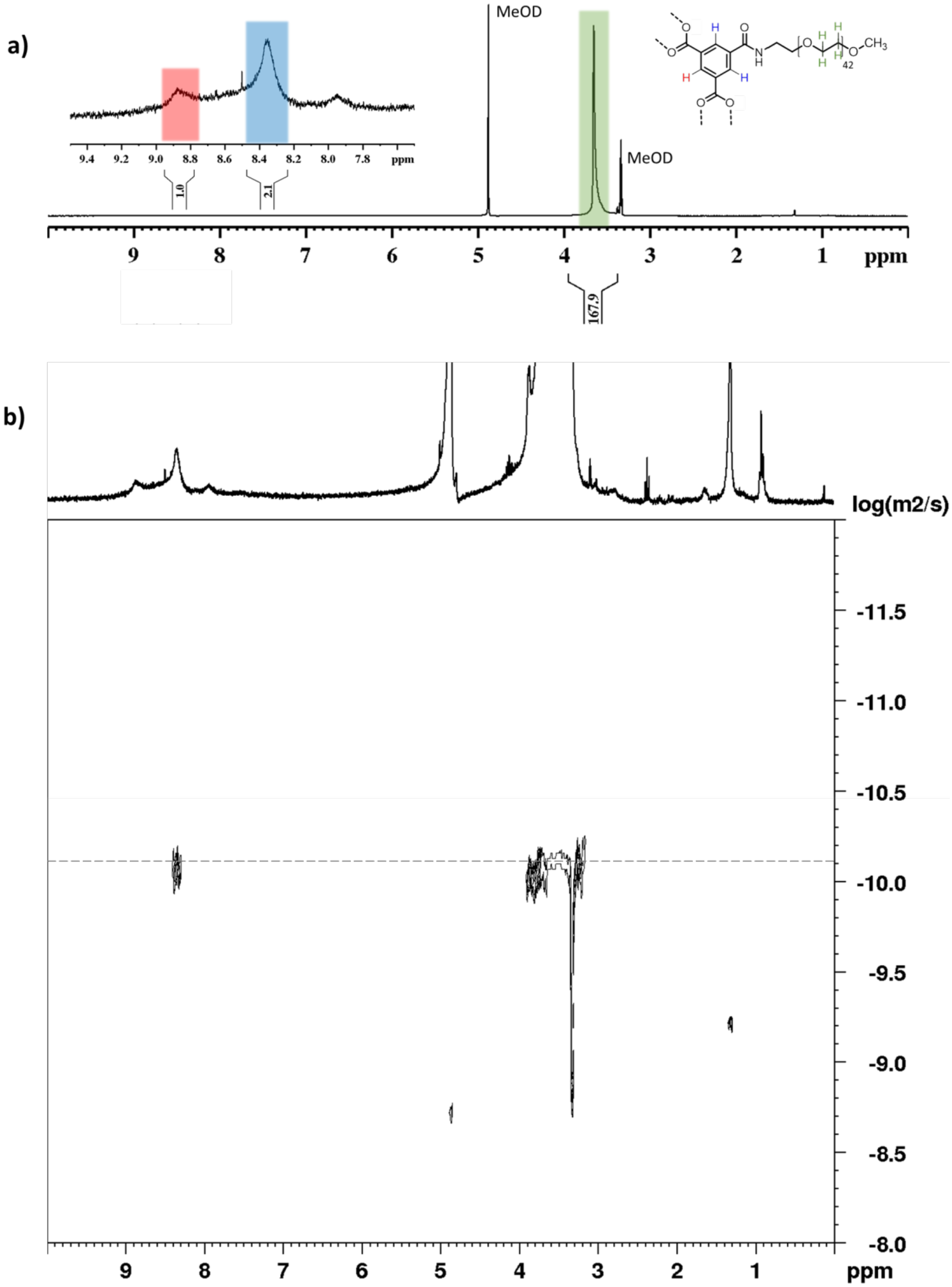

Figure S2. a) ^{1}H NMR (300 MHz, 25 ºC) spectrum of BCN-93 in MeOD, showing the expected integration of the aromatic backbone (highlighted in red and blue) and the polymer chain (highlighted in green, with 168 H corresponding to 42 repeating units and a conversion of 100 %). b) DOSY NMR spectrum of BCN-93 in MeOD, showing a diffusion coefficient of D = 7.59 10-11 m^2 s^{-1}. The stokes radius determined for BCN-93 in MeOD was 5.25 nm.

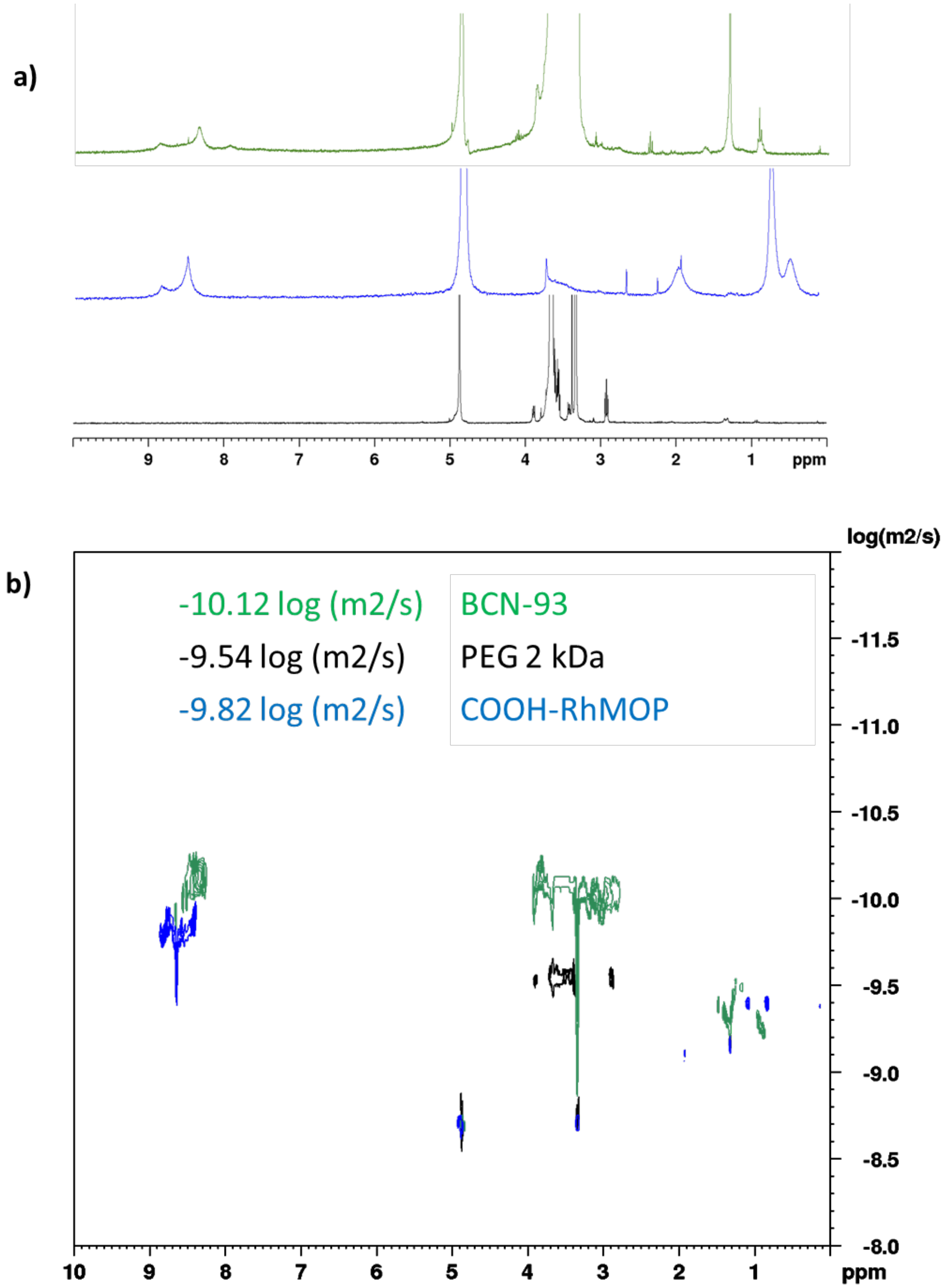

Figure S3. a) ^{1}H NMR (300 MHz, 25 ºC) spectra of BCN-93 (green), COOH-RhMOP (blue) and PEG (black) in MeOD. b) DOSY NMR spectrum (300 MHz, 25 ºC) of BCN-93 (green) in MeOD, showing an increase in the diffusion coefficient (D = 7.59 10^{-11} m^2 s^{-1}) with respect to free PEG (black, D = 2.88 10^{-10} m^2 s^{-1}) and COOH-RhMOP (blue, D= 1.51 10^{-10} m^2 s^{-1}) precursors.

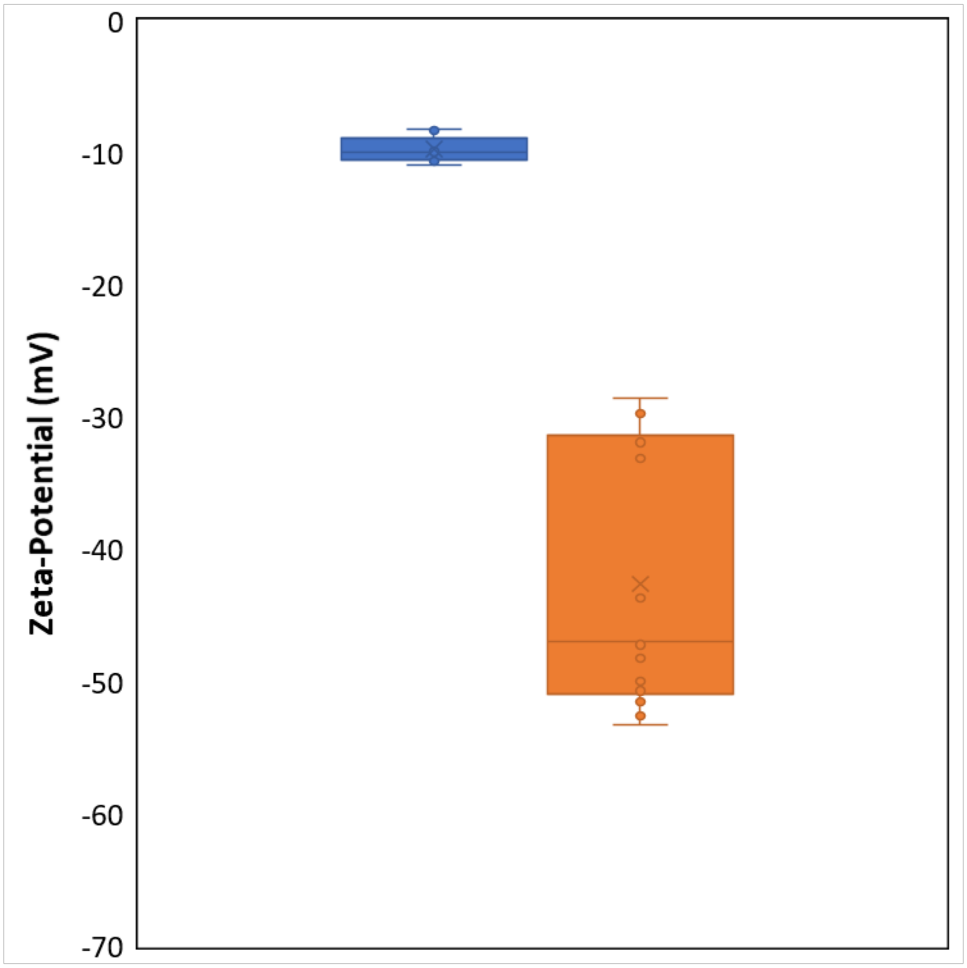

Figure S4. Zeta-Potential of BCN-93 (blue) and the starting COOH-RhMOP (orange), indicating a clear increase in zeta-potential due to the coupling of the free carboxylic groups with neutral PEG chains. Standard deviation was obtained from at least nine different measurements. All measurements were done in basic water (pH = 8).

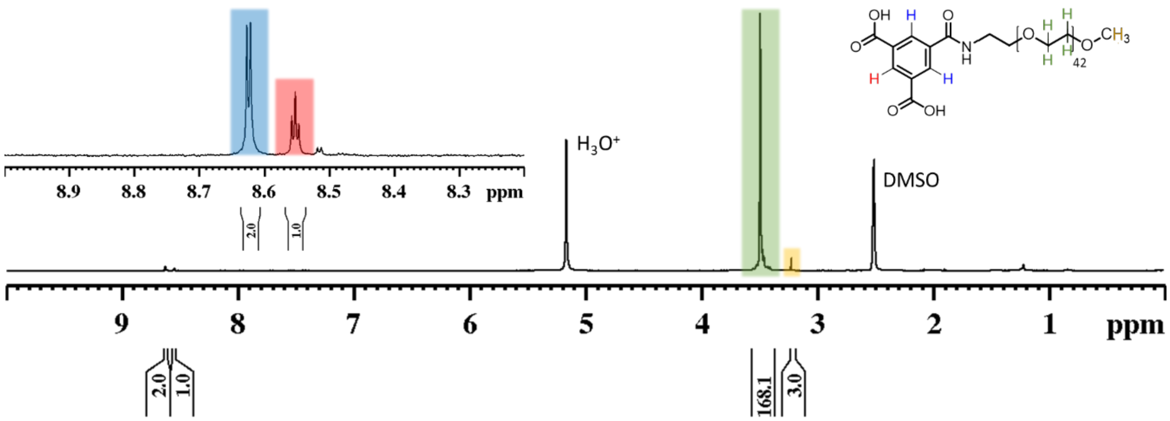

Figure S5. a) Acid digestion (^{1}H NMR spectrum in DMSO-d6) of BCN-93, showing the integration corresponding to 100% conversion. Signals highlighted in blue and red correspond to the aromatic backbone (1 H (d) and 2H (t)). Signals highlighted in green correspond to the PEG repeating unit (168 H (s) corresponding to 42 units). And signals highlighted in yellow depict the terminal methoxy group (3H (s)).

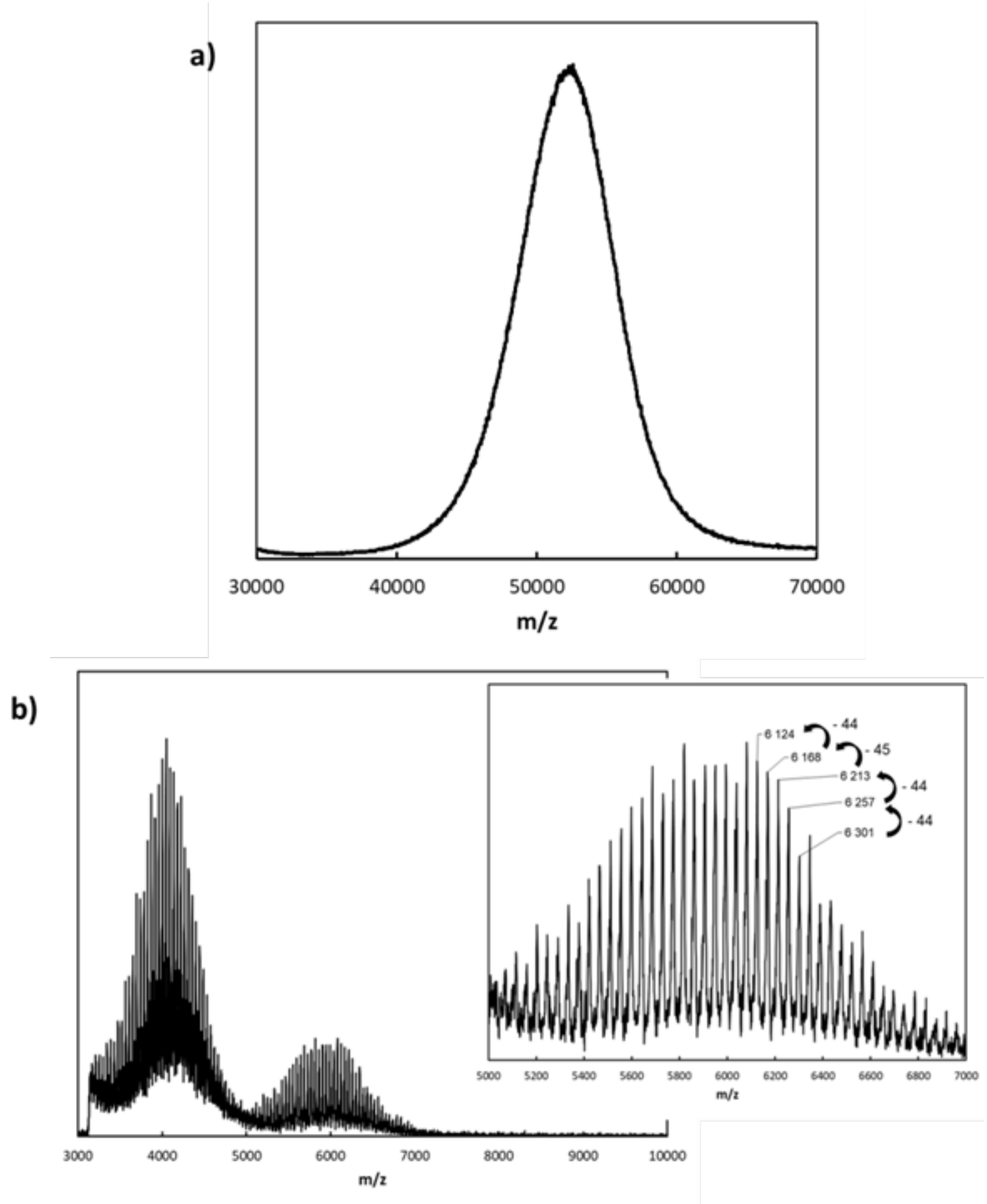

Figure S6. a) MALDI-TOF spectrum of BCN-93 with a center of the mass centered at 53272 m/z. (Expected for $Rh_{24}(PEG_{2000}\text{-}BDC)_{24}]$ = 55513 ± 4800 m/z). Note that the starting NH_2-mPEG (2 kDa) has a molecular weight distribution of 2000 ± 200 g mol^{-1}, which explains the broadness of the MALDI-TOF peak. b) MALDI-TOF spectrum in the m/z range from 3000 to 10000, showing ionization fragments from BCN-93. Note that these fragments correspond to PEGylated molecules as the separation between peaks correspond to a PEG repeating unit (44 m/z).

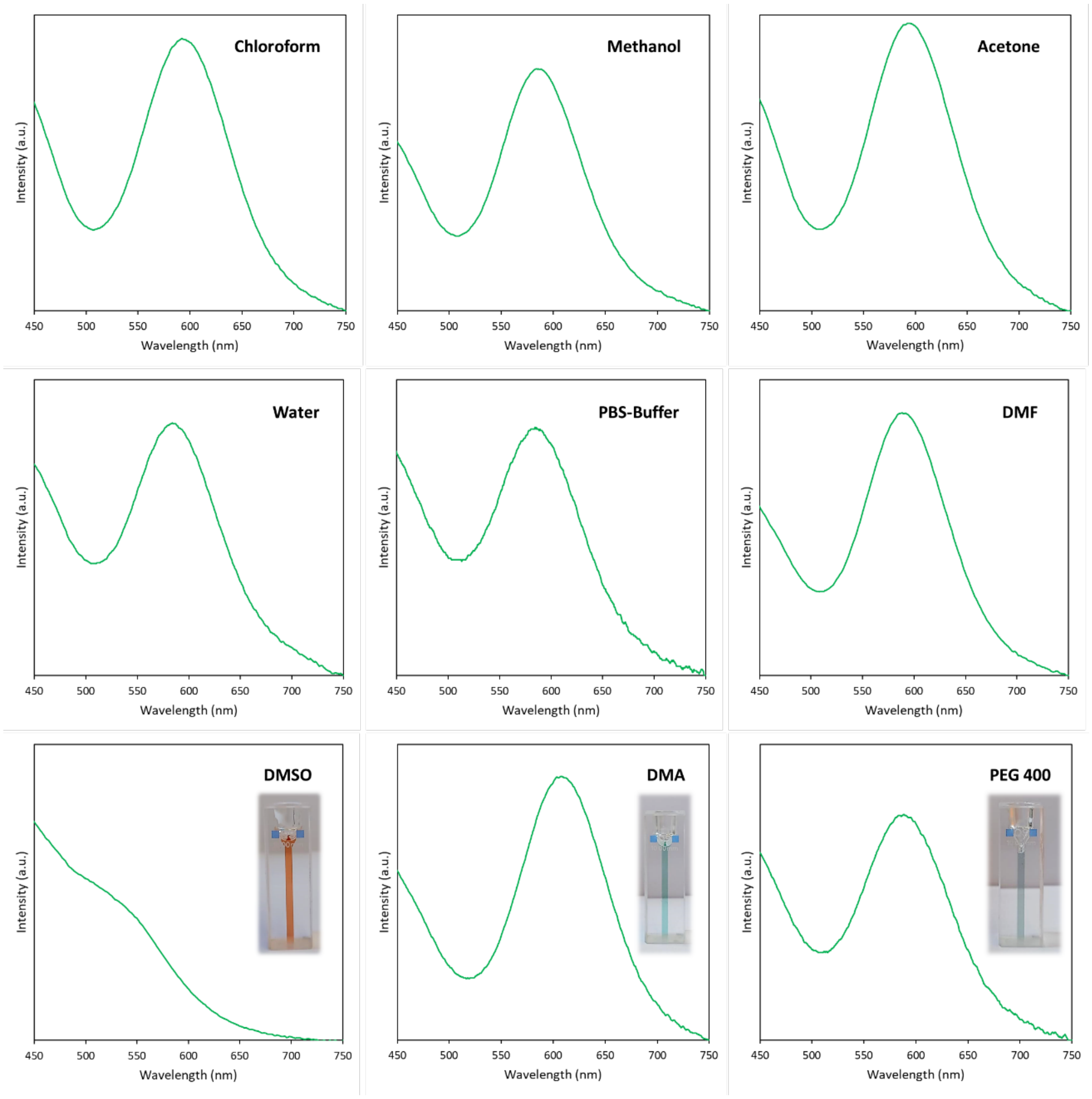

Figure S7. UV/Vis spectra of the synthesized BCN-93 (green) in a variety of organic and aqueous solvents, as indicated. All UV/Vis spectra show the typical λ_{max} centered at *ca.* 599 nm of the Rh(II) paddlewheel; except for DMSO, where the coordination of DMSO is causing a shift in λ_{max} (as also visible by the change of the color from green to red) .

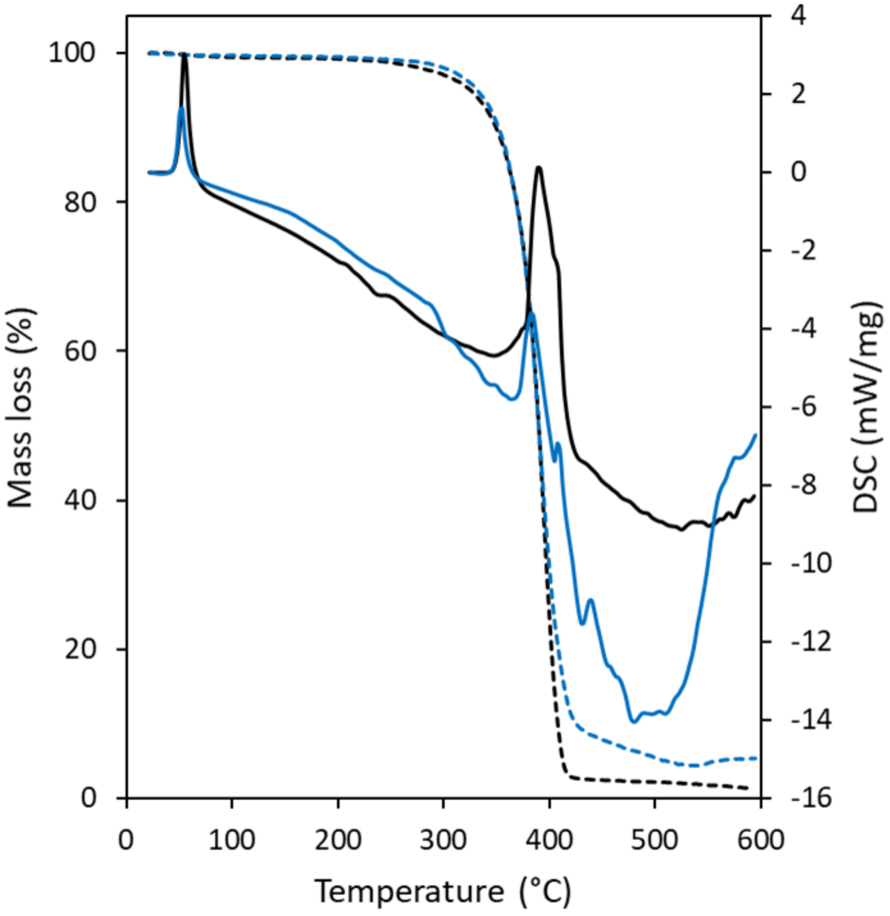

Figure S8. TGA (dotted line) and DSC (solid line) curves of the pure PEG polymer (black) and BCN-93 (blue), showing a similar decomposition temperature of around 400 ºC. Note that there is no mass loss upon melting (signal in the DSC curve at around 50 °C) of PEG and BCN-93.

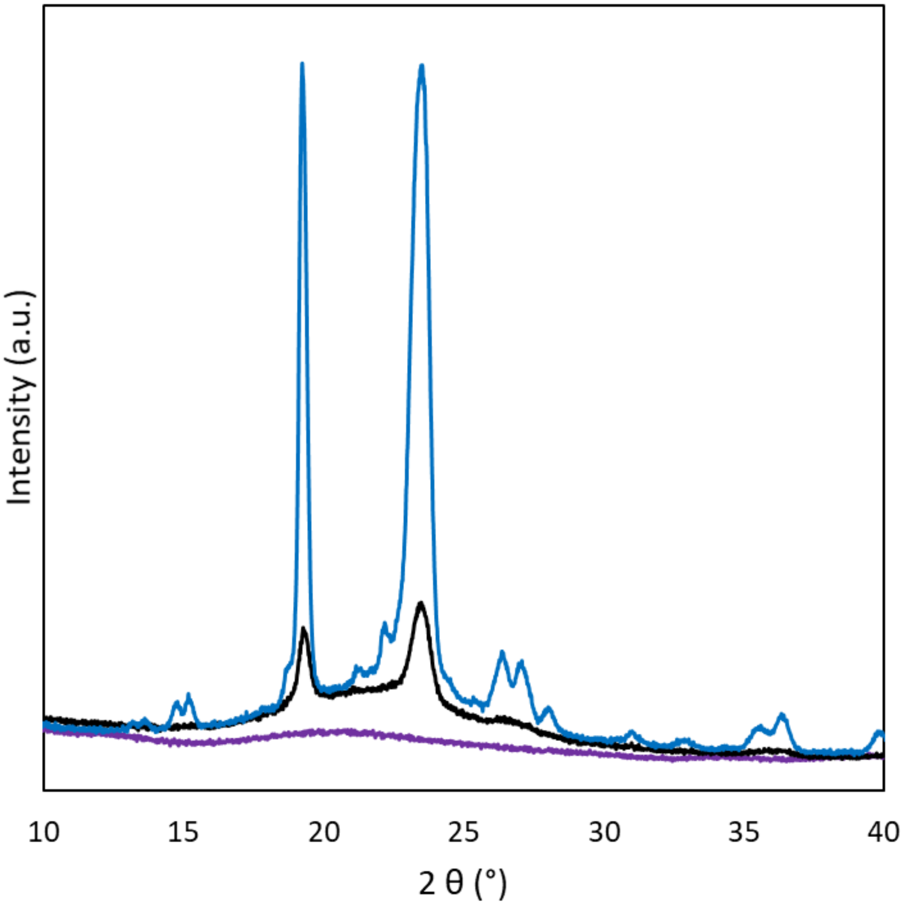

Figure S9. XRPD patterns of COOH-RhMOP (purple), PEG (2 kDa, blue) and as-made semicrystalline BCN-93 (black).

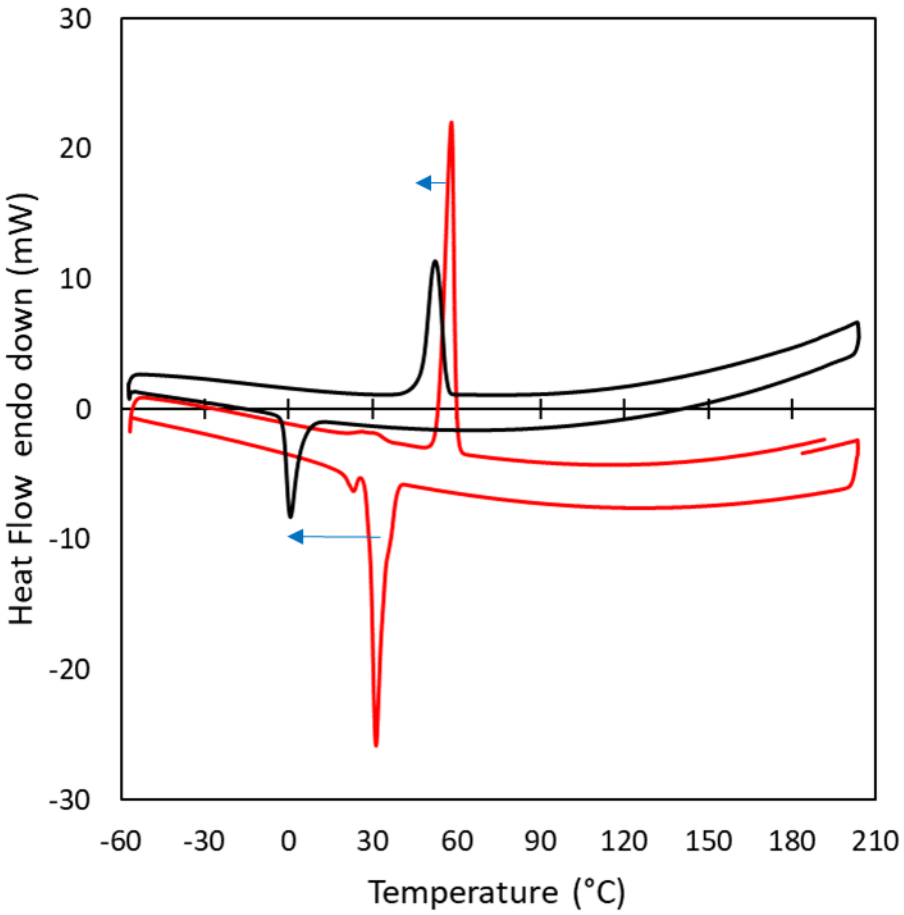

Figure S10. DSC curve of the pure PEG (2 kDa) polymer (red) and as-made BCN-93 (black). Blue arrows indicate the shift in crystallization (from 31 to 1 °C) and melting temperatures (from 60 to 47 °C). Furthermore, the lower intensity and area of the melting and crystallization peaks of BCN-93 compared to pure PEG (2 kDa) indicate that BCN-93 has a lower degree of crystallinity than the initial PEG (2 kDa) precursor. This decrease in crystallinity is ascribed to the radial organization of PEG chains on the surface of BCN-93.

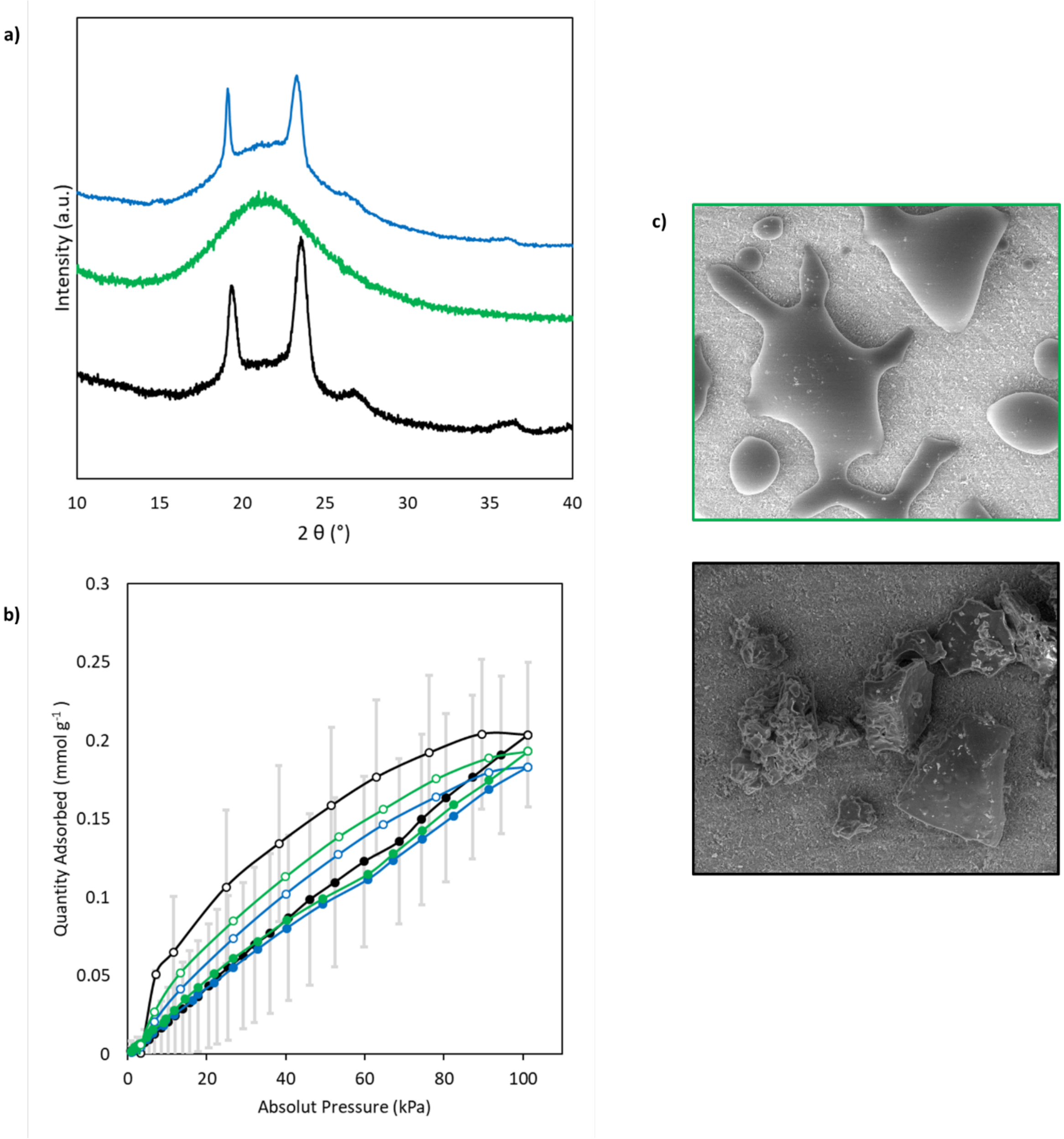

Figure S11. a) XRPD of the as-made semicrystalline BCN-93 powder (black), post-melted BCN-93 (amorphous supercooled liquid) (green) and the material obtained after freezing (at -85 ºC) and lyophilizing the amorphous supercooled liquid to recover the crystalline phase (blue). b) CO_2 adsorption measured at 298 K of BCN-93 powder (black), post-melted BCN-93 (amorphous supercooled liquid) (green) and the re-lyophilized, semicrystalline BCN-93 (blue). Error bars indicate the standard deviation observed from three independent samples. c) VT-SEM images of the initial as-made semicrystalline BCN-93 powder (bottom) and the same sample after being melted *in situ* (top).

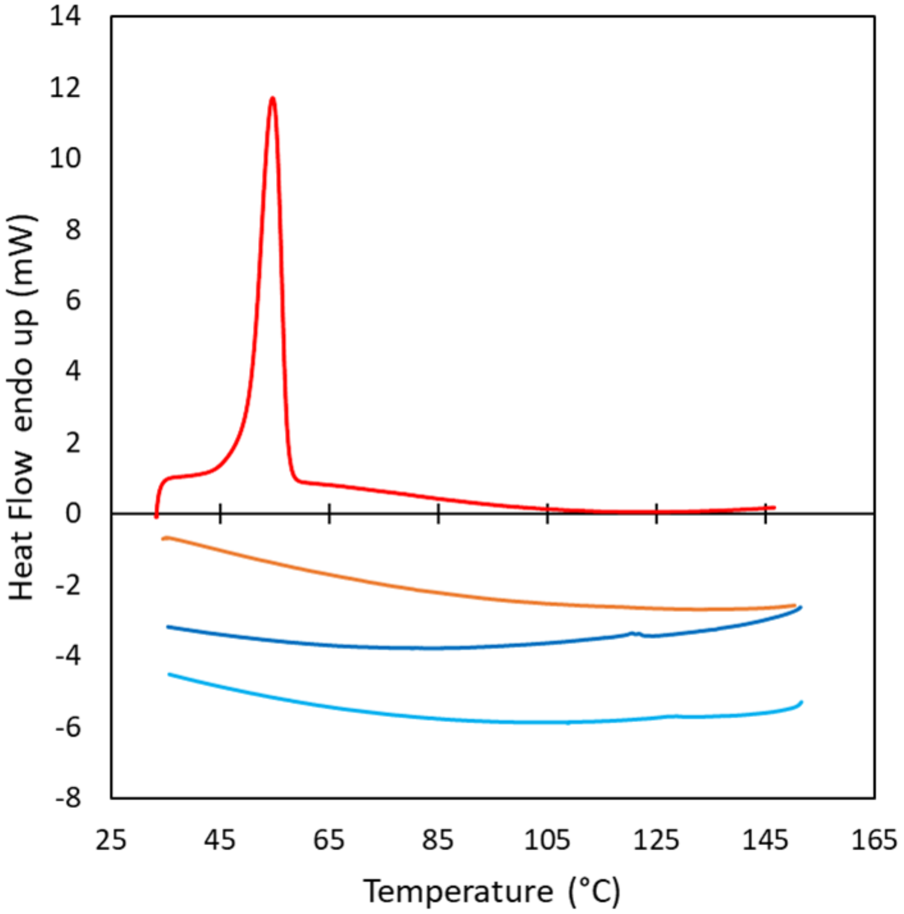

Figure S12. DSC curve of BCN-93 showing the absence of melting (orange) and crystallization (blue) when the sample is only cooled down to RT; 1st heating (red), 2nd heating (orange), 1st cooling (dark blue) and 2nd cooling (light blue).

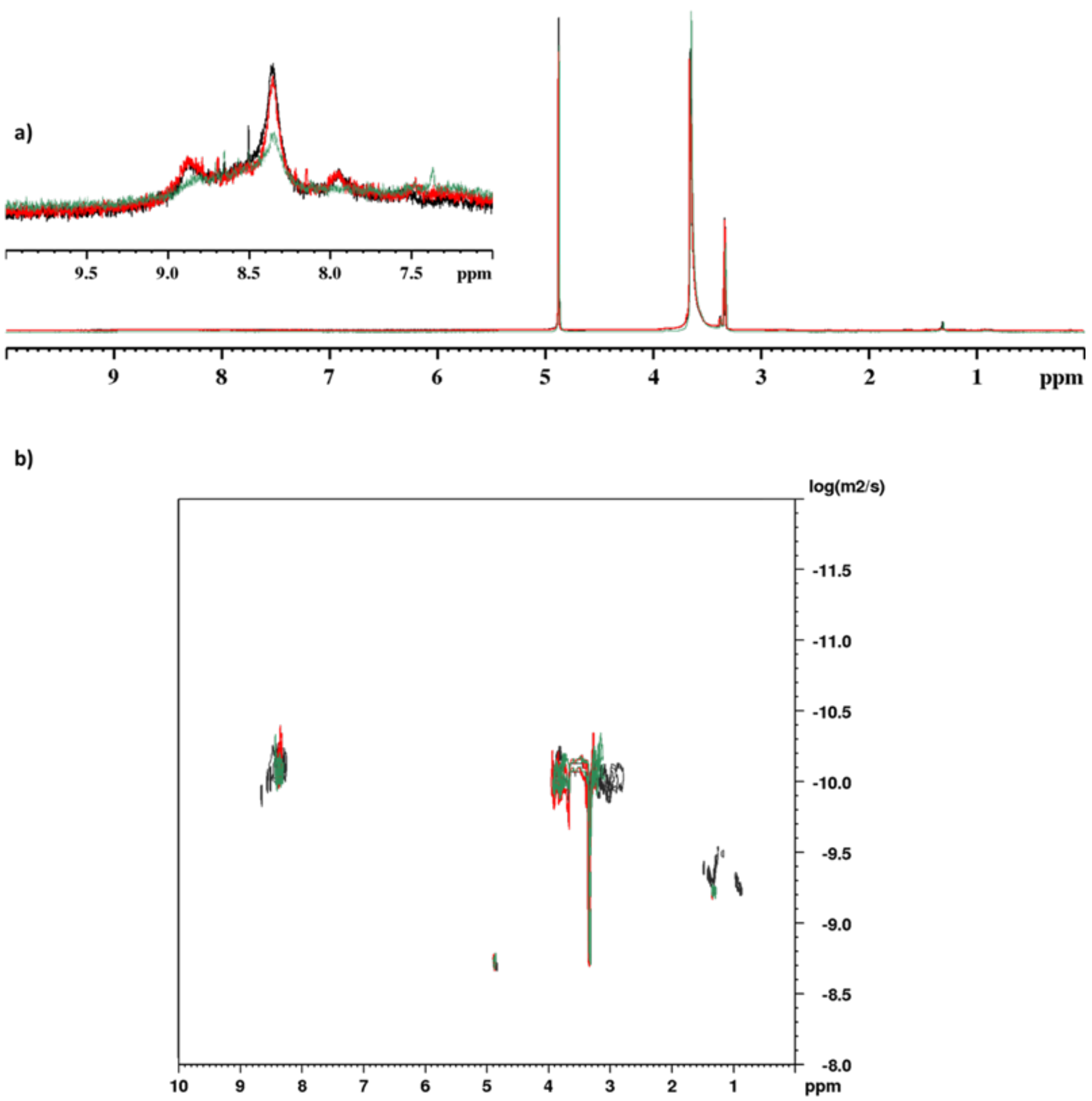

Figure S13. a) ^{1}H NMR spectra (300 MHz, 25 ºC) of BCN-93 before (black), after the melting/cooling process (red) and after grinding (green), measured in MeOD, and b) the corresponding DOSY spectra, showing the preservation of the structure throughout all the processes.

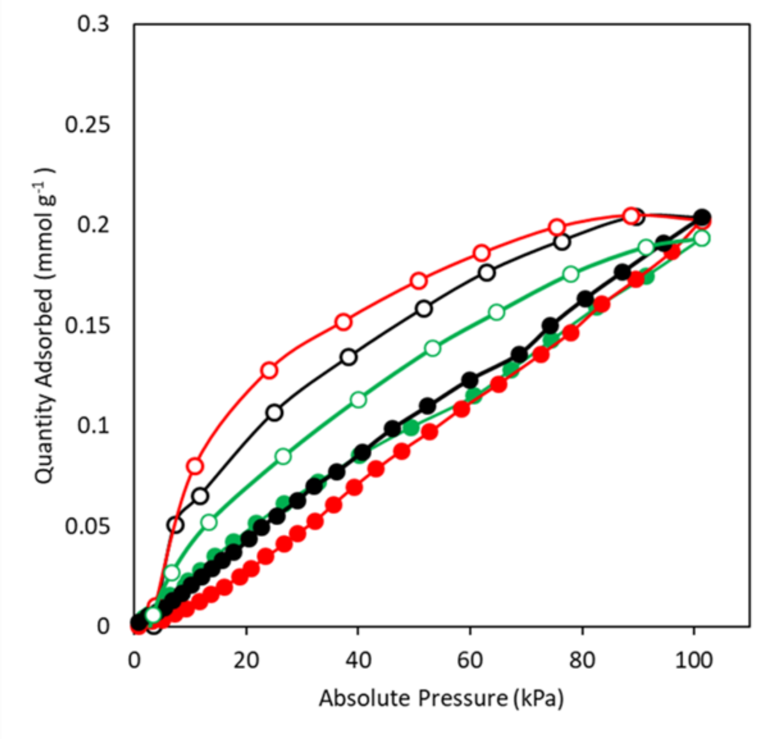

Figure S14. CO_2-sorption isotherms at 298 K of BCN-93 before (black) and after the melting/cooling process (red), and the grinded BCN-93 (green).

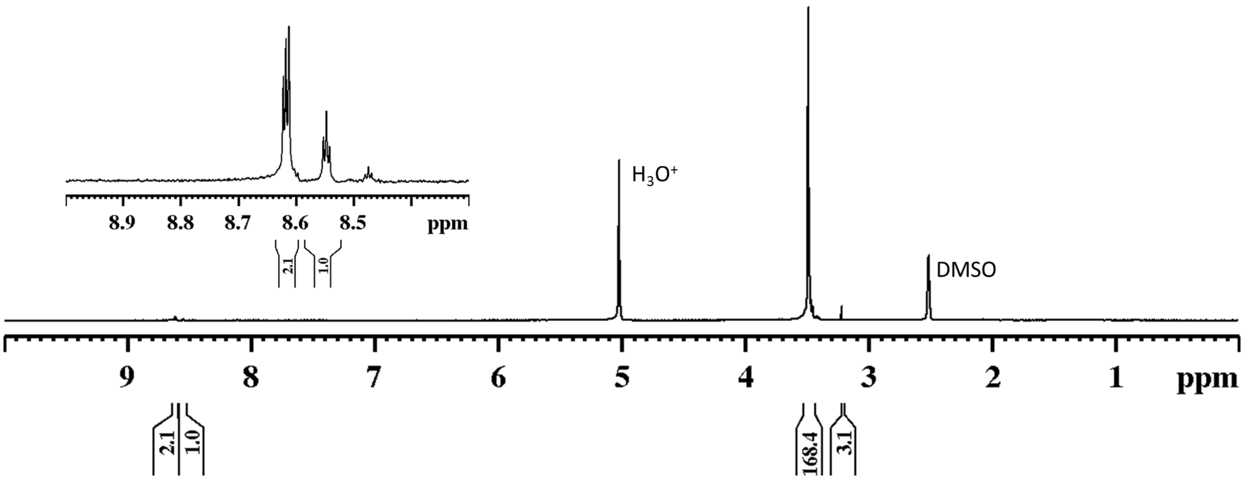

Figure S15. ^{1}H NMR spectrum in DMSO-d6 of acid digested BCN-93 after melting. Note that the integration of the aromatic backbone (1 H (d) and 2 H (t)), the terminal methoxy group (3 H (s)) and the polymeric repeating unit (168 H (s)) corresponding to 100% conversion and 24 PEG chains per MOP unit is maintained.

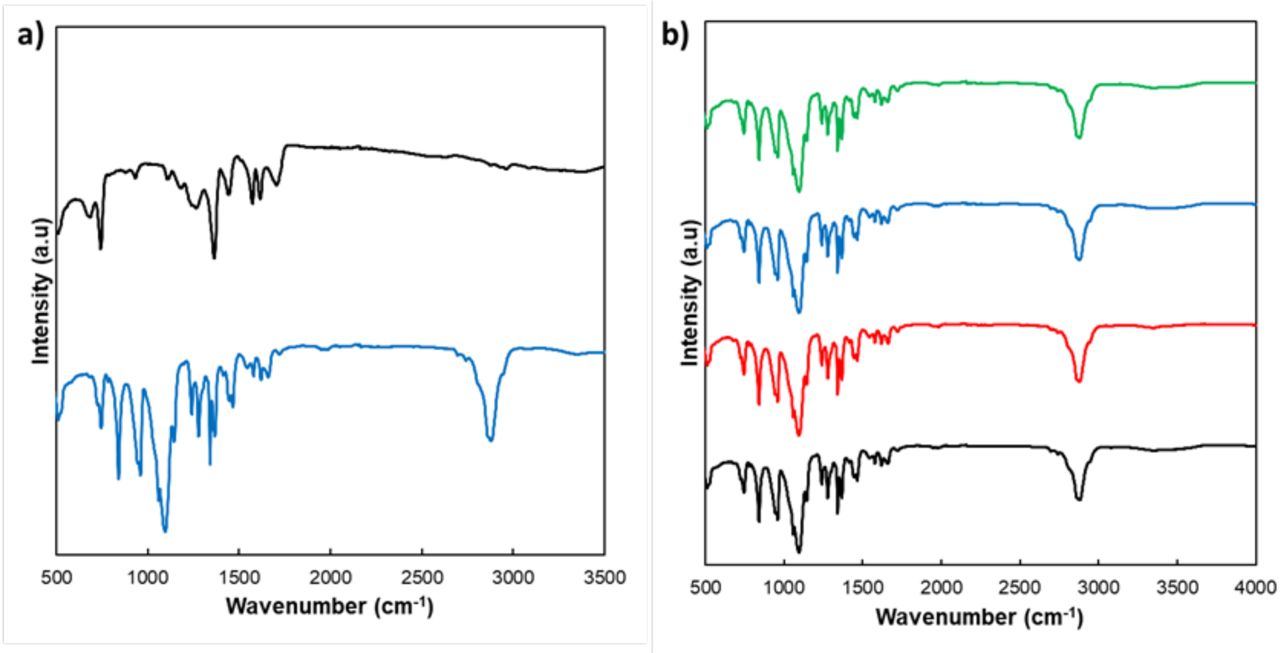

Figure S16. a) FT-IR spectra of the starting COOH-RhMOP (black) and BCN-93 (blue). Note that the peak ascribed to free carboxylic acid stretching at 1750 cm^{-1} in the COOH-RhMOP disappears in BCN-93 due to the formation of the amide bond, which has a characteristic stretching vibration at 1566 cm^{-1}. In addition, a new peak at 2887 cm^{-1} ascribed to the C-H stretching appears in BCN-93 due to the presence of PEG aliphatic chains. b) FT-IR spectra of BCN-93 in all its distinct forms; as semicrystalline powder (black), melted (red), lyophilized (blue) and grinded (green).

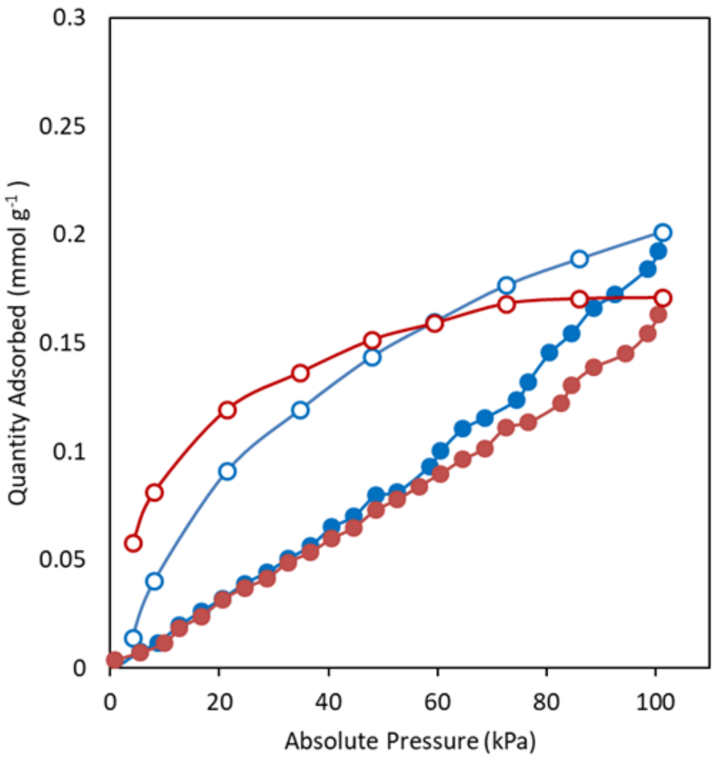

Figure S17. CO_2-sorption isotherms at 298 K of as-made semi-crystalline BCN-93 (blue) and the same sample heated and cooled down for four times (red), confirming its integrity upon four consecutive melting/cooling cycles.

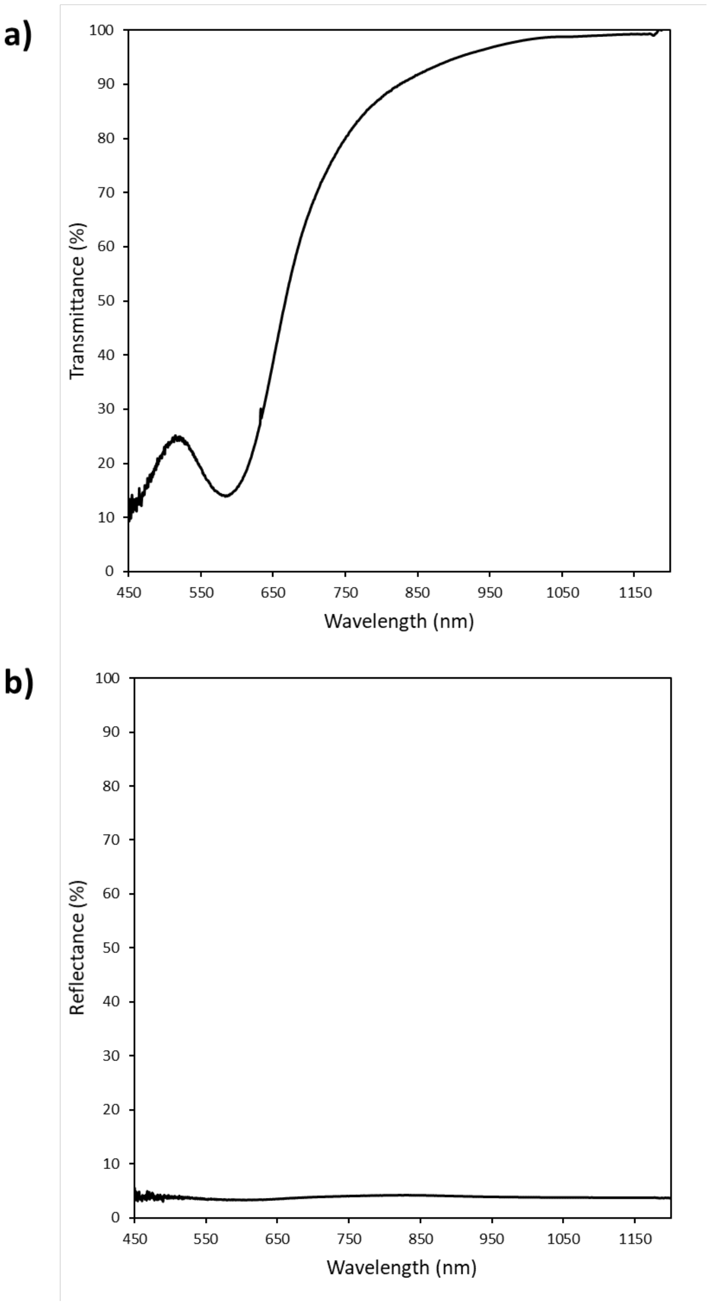

Figure S18. a) Transmittance spectrum of an amorphous BCN-93 film prepared after melting, revealing a transmittance of 100% above 900 nm. Note that the peak in transmittance at 584 nm corresponds to the adsorption band of the Rh-Rh paddlewheel. b) Reflectance spectrum of an amorphous BCN-93 film prepared after melting.

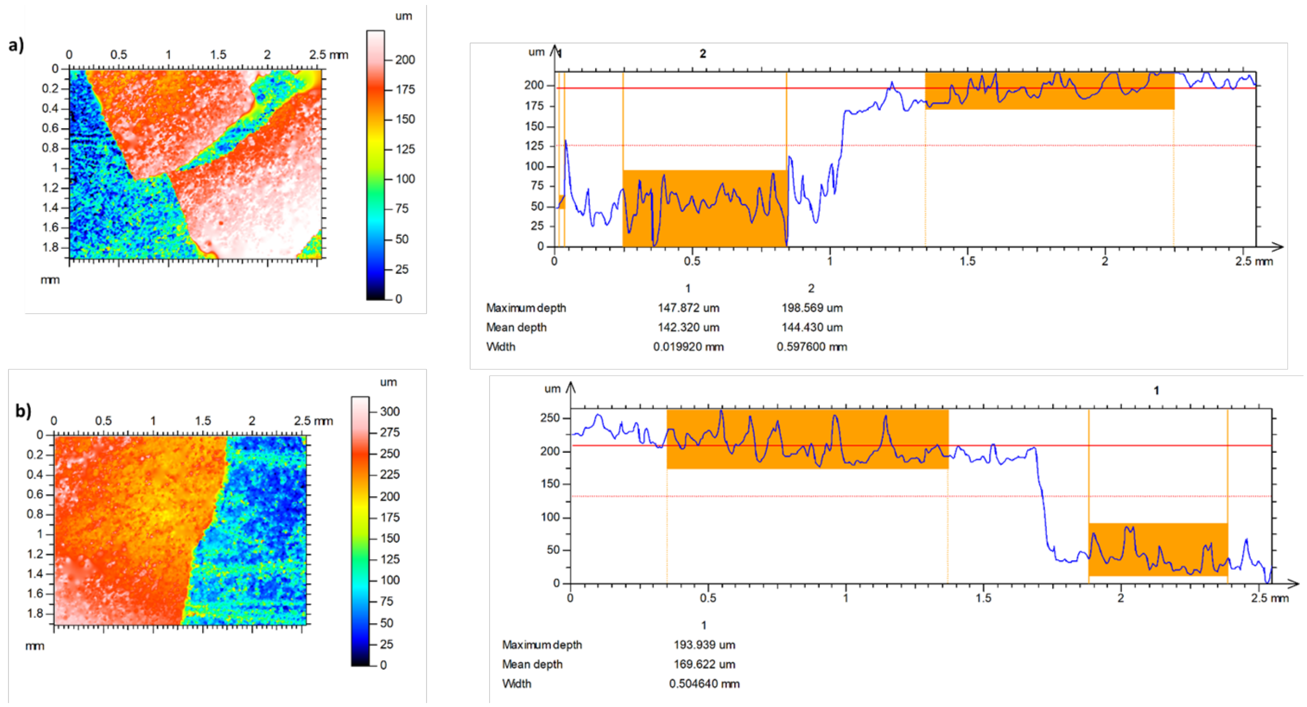

Figure S19. a) and b) correspond to two height mappings of two independent BCN-93 amorphous films (left) and their corresponding height profile (right).

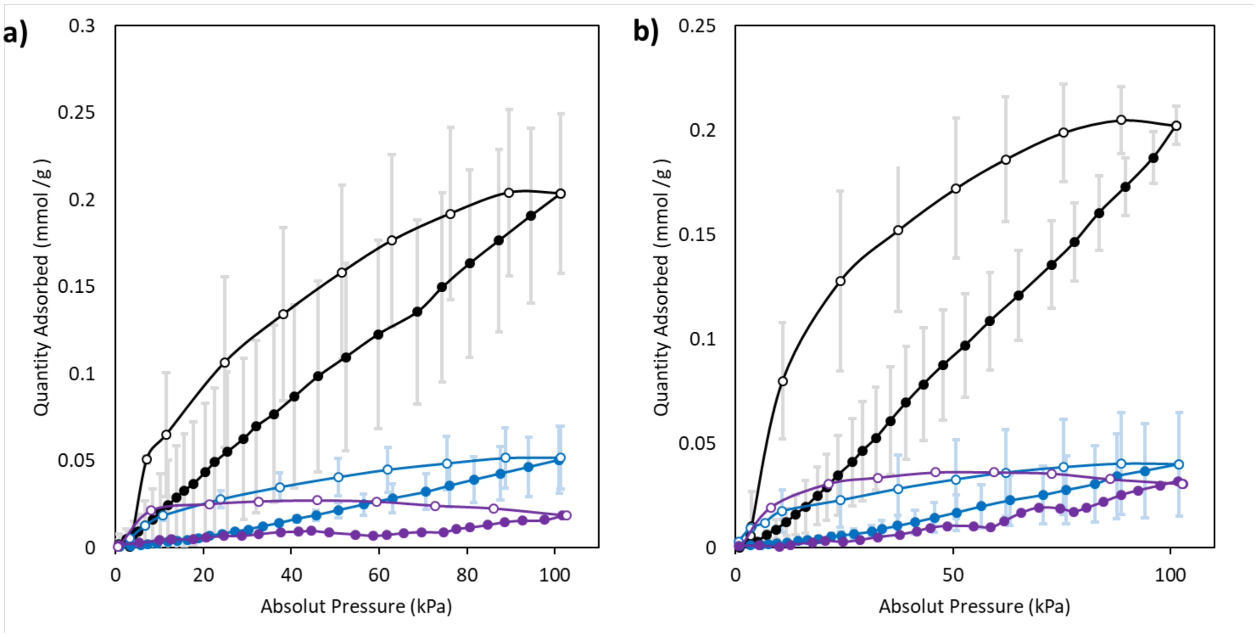

Figure S20. a) CO_2 adsorption isotherm of the physical mixture containing COOH-RhMOP (CO_2 uptake at 1 bar = 0.85 mmol g^{-1}) and PEG (2 kDa) (purple); pure PEG (2 kDa) (blue); and semicrystalline BCN-93 (black). All these samples were activated under vacuum at room temperature prior measurement to preserve the semicrystalline state of BCN-93. b) CO_2 adsorption isotherm of the physical mixture containing COOH-RhMOP (CO_2 uptake at 1 bar = 0.58 mmol g^{-1}) and PEG (2 kDa) (purple); pure PEG (2 kDa) (blue); and amorphous supercooled BCN-93 (black). All these samples were activated at 85ºC under vacuum prior measurement.

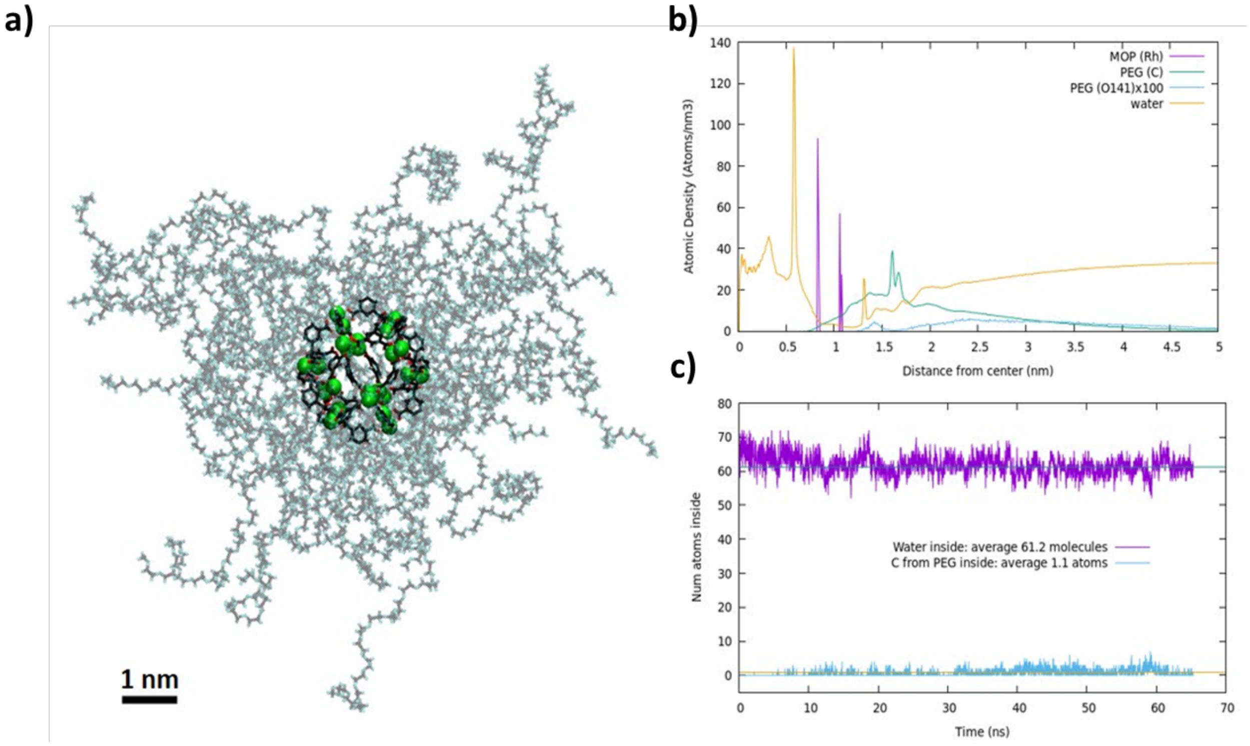

Figure S21. a) Snapshot of the equilibrium configuration of BCN-93 at 298 K in water. Water is not shown for simplicity. Color code: Rhodium (green); MOP carbon (black); PEG carbon (cyan), hydrogen (white); oxygen (red). Rhodium atoms have been emphasized as Van der Waals spheres. PEG atoms are translucent for clarity. b) Radial density profile of Rhodium atoms, carbon atoms of PEG chains, the terminal oxygen of the PEG chains and oxygen atoms from water. c) Number of C atoms from PEG and water molecules inside the MOP cavity as a function of time during the production run. These simulations show that the inner cavity is mainly filled with water when BCN-93 is solubilized in water.

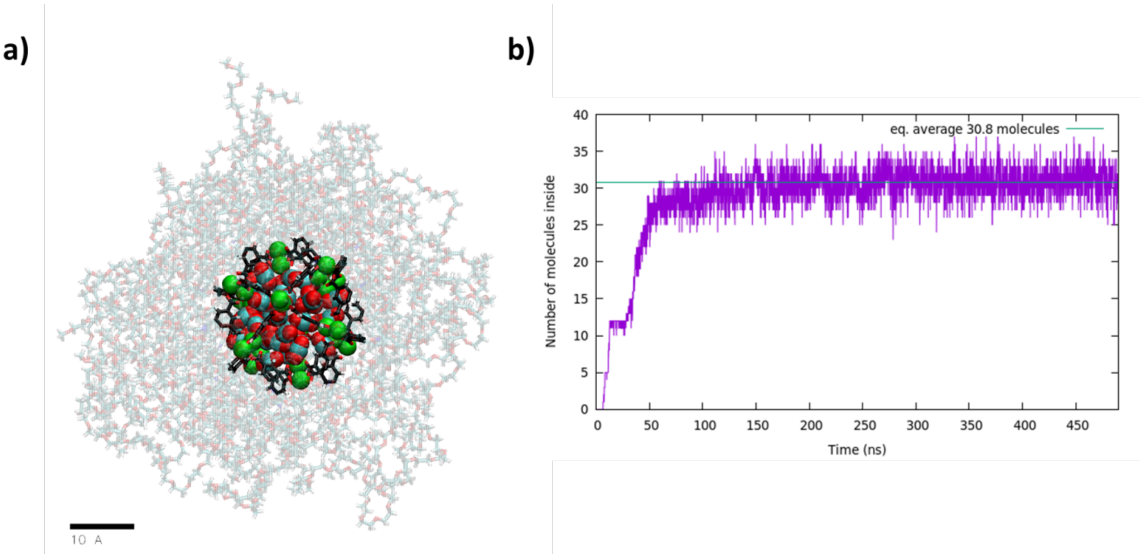

Figure S22. a) Snapshot of the equilibrium configuration obtained after exposing the evacuated BCN-93 to CO_2. The CO_2 molecules inside the cavity are emphasized in Van der Waals representation (molecules outside the cavity are not shown). Color code: Rhodium (green); MOP carbon (black); PEG and CO_2 carbon (cyan), hydrogen (white); and oxygen (red). Rhodium atoms have been emphasized as Van der Waals spheres. PEG atoms are translucent for clarity. b) Number of CO_2 molecules inside the MOP cavity as a function of time (the equilibrium average is calculated over the last 500 ns). This simulation show that CO_2 is able to diffuse through the PEG shell into the MOP cavity.

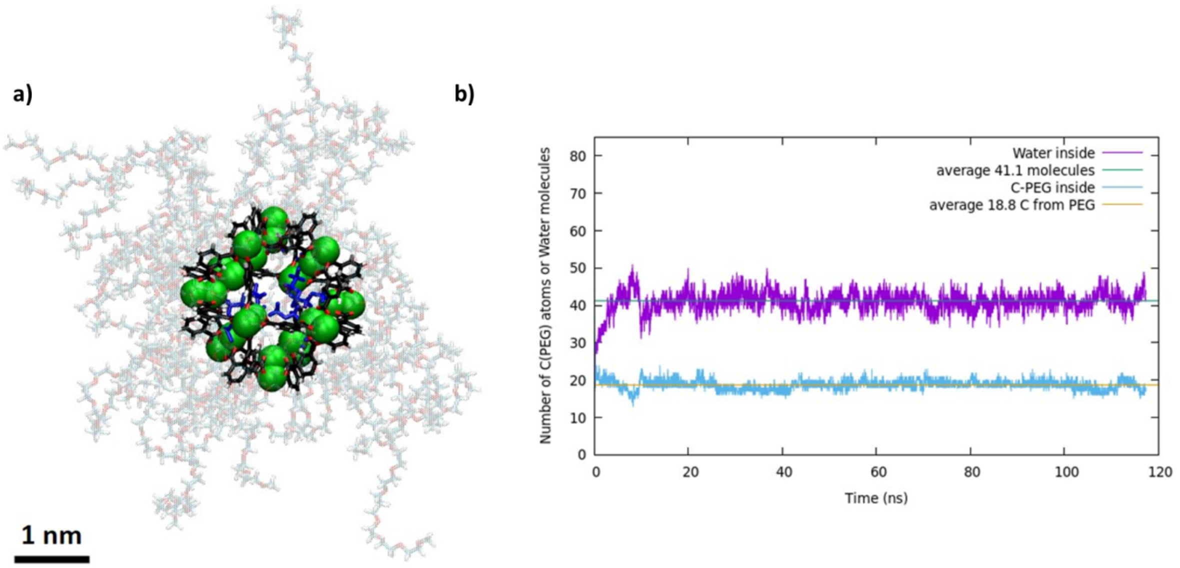

Figure S23. a) Snapshot of the equilibrium configuration of H-RhMOP in water at 298 K exposed to 10 free PEG chains. Color code: Rhodium (green); MOP carbon atoms (black); PEG carbon atoms (cyan); hydrogen (white); and oxygen (red). Rhodium atoms have been emphasized as Van der Waals spheres. PEG atoms inside the MOP structure are highlighted in blue. PEG atoms outside the MOP are translucent. c) Number of C atoms from PEG and water molecules inside the MOP cavity as a function of time during the production run.

S3. Characterization of the amorphous composite films

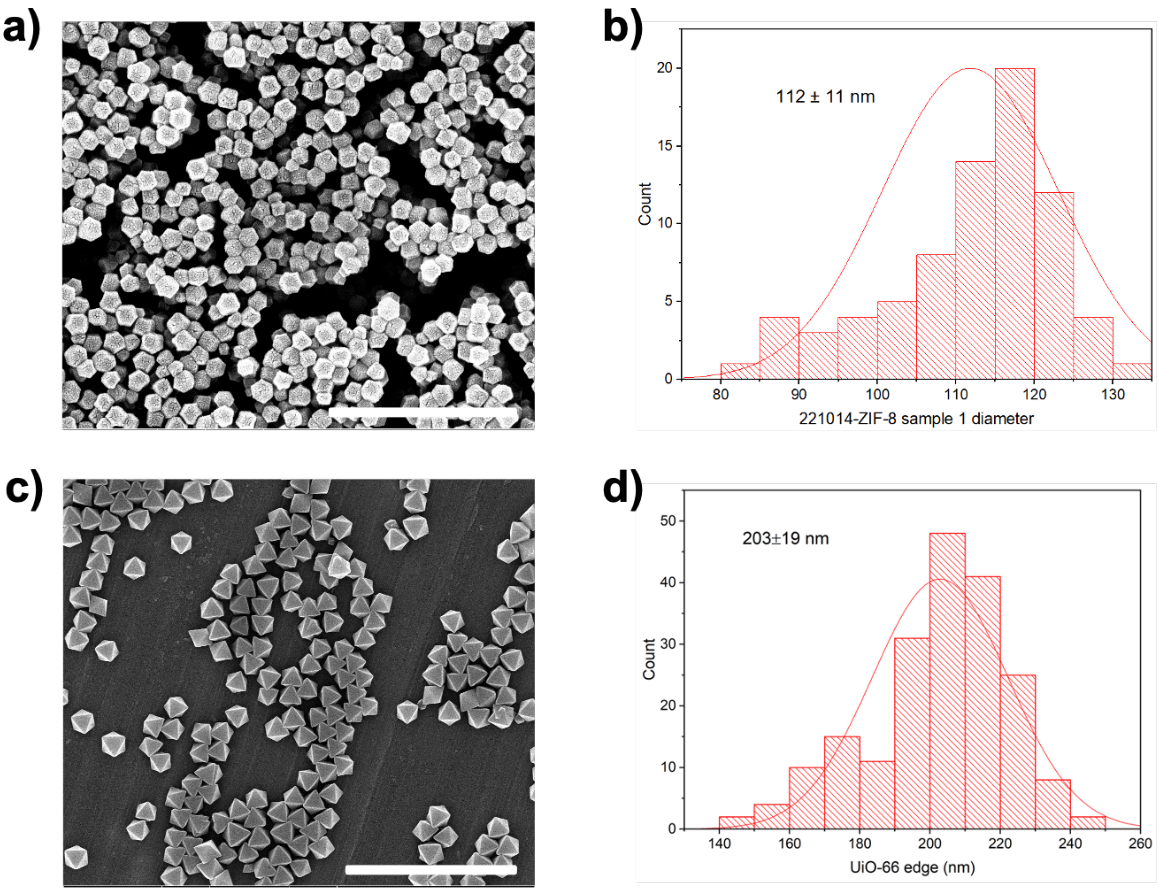

Figure S24. a) SEM image of the synthesized ZIF-8 particles. Scale bar: 1 μm b) Corresponding histogram revealing an average size of 112 nm. c) SEM image of the synthesized UiO-66 particles. Scale bar: 2 μm. d) Corresponding histogram revealing an average size of 203 nm.

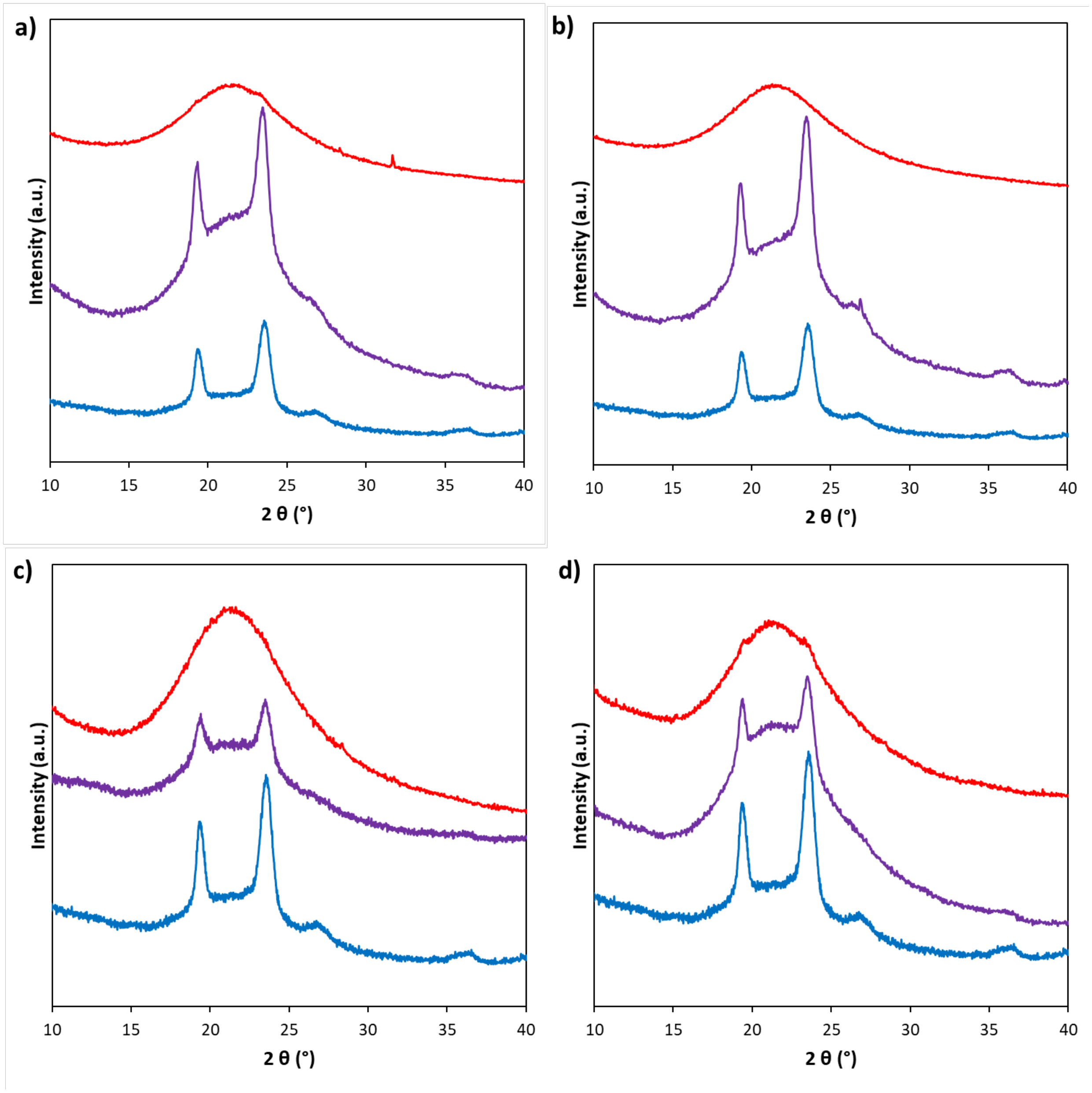

Figure S25. a) XRPD patterns of as-made semicrystalline BCN-93 (blue), the semicrystalline BCN-93 mixed with additive (purple), and the composite amorphous film obtained after the melting-cooling process (red) for 10 wt% PEI (a), 20 wt% PEI (b), 10 wt% OH-RhMOP (c) and 20 wt% OH-RhMOP (d).

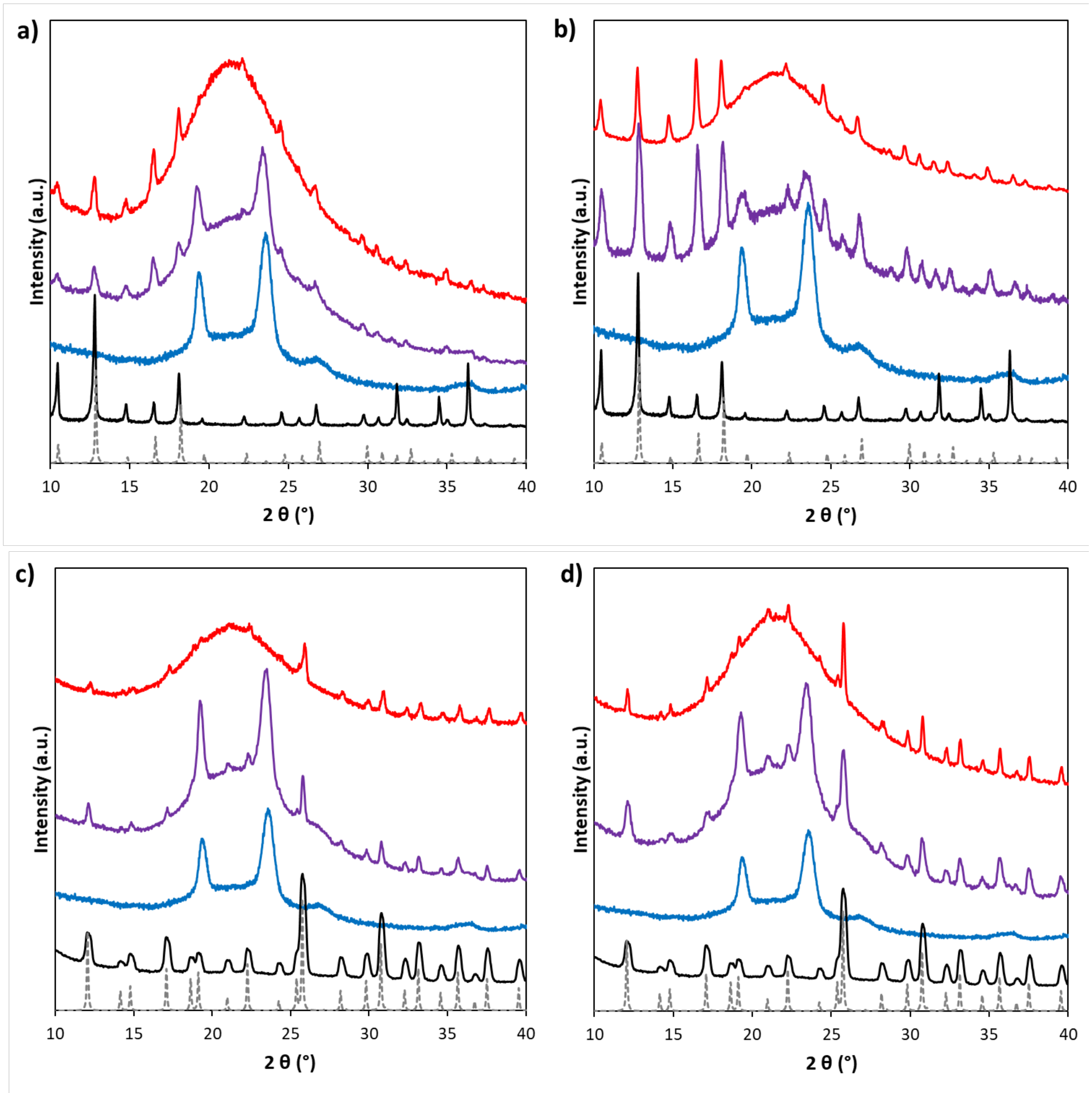

Figure S26. Simulated XRPD of MOF additives (grey dotted line) and experimental XRPD patterns of MOF additives (black); as-made semicrystalline BCN-93 (blue); the semicrystalline BCN-93 mixed with additive (purple); and the amorphous composite film obtained after the melting-cooling process (red) for 10 wt% ZIF-8 (a), 20 wt% ZIF-8 (b), 10 wt% UiO-66 (c) and 20 wt% UiO-66 (d).

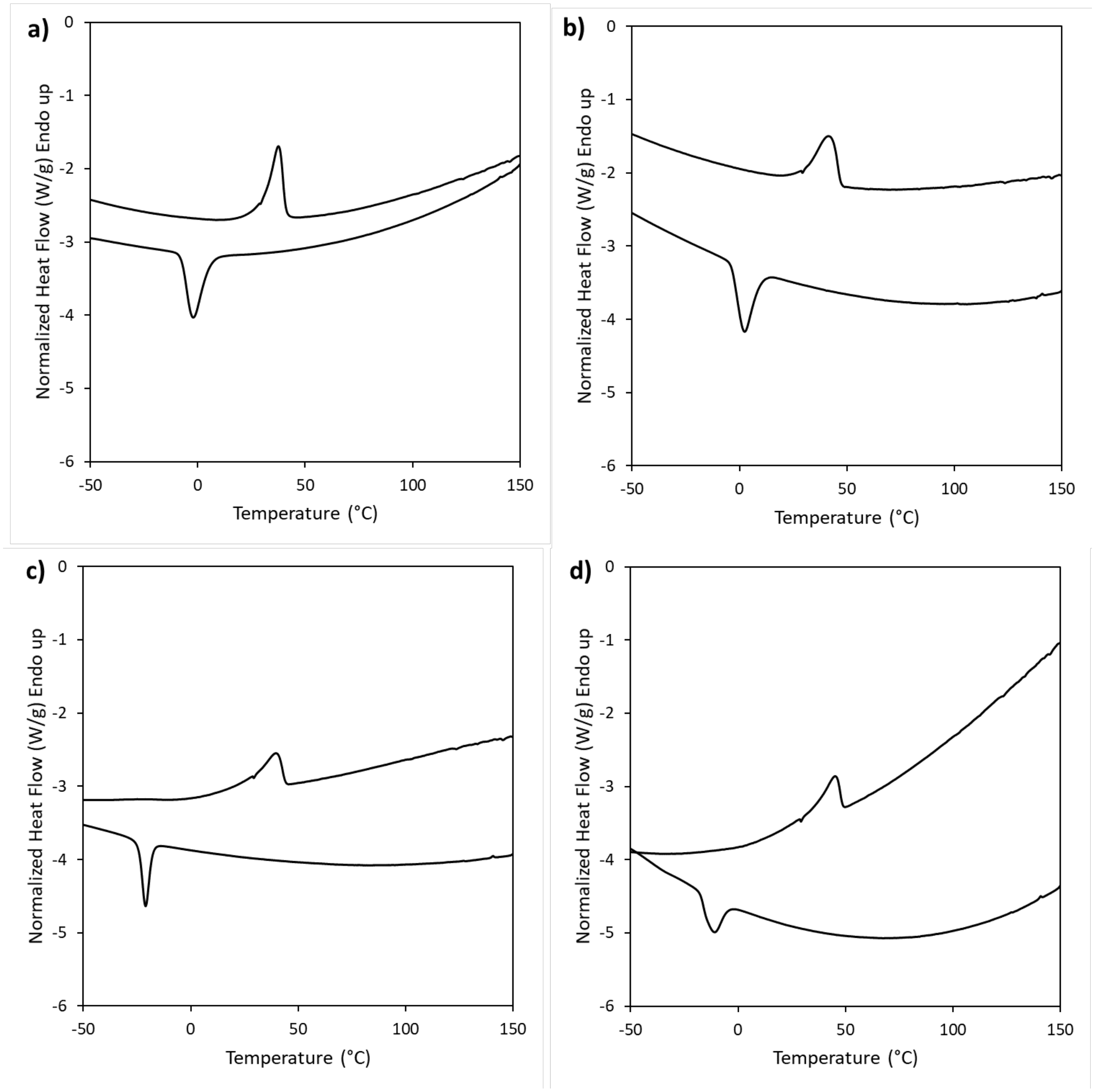

Figure S27. DSC curves of semicrystalline BCN-93 mixed with 10 wt% PEI (a), 20 wt% PEI (b), 10 wt% OH-RhMOP (c) and 20 wt% OH-RhMOP (d). In all cases, the melting and crystallization peaks are observed.

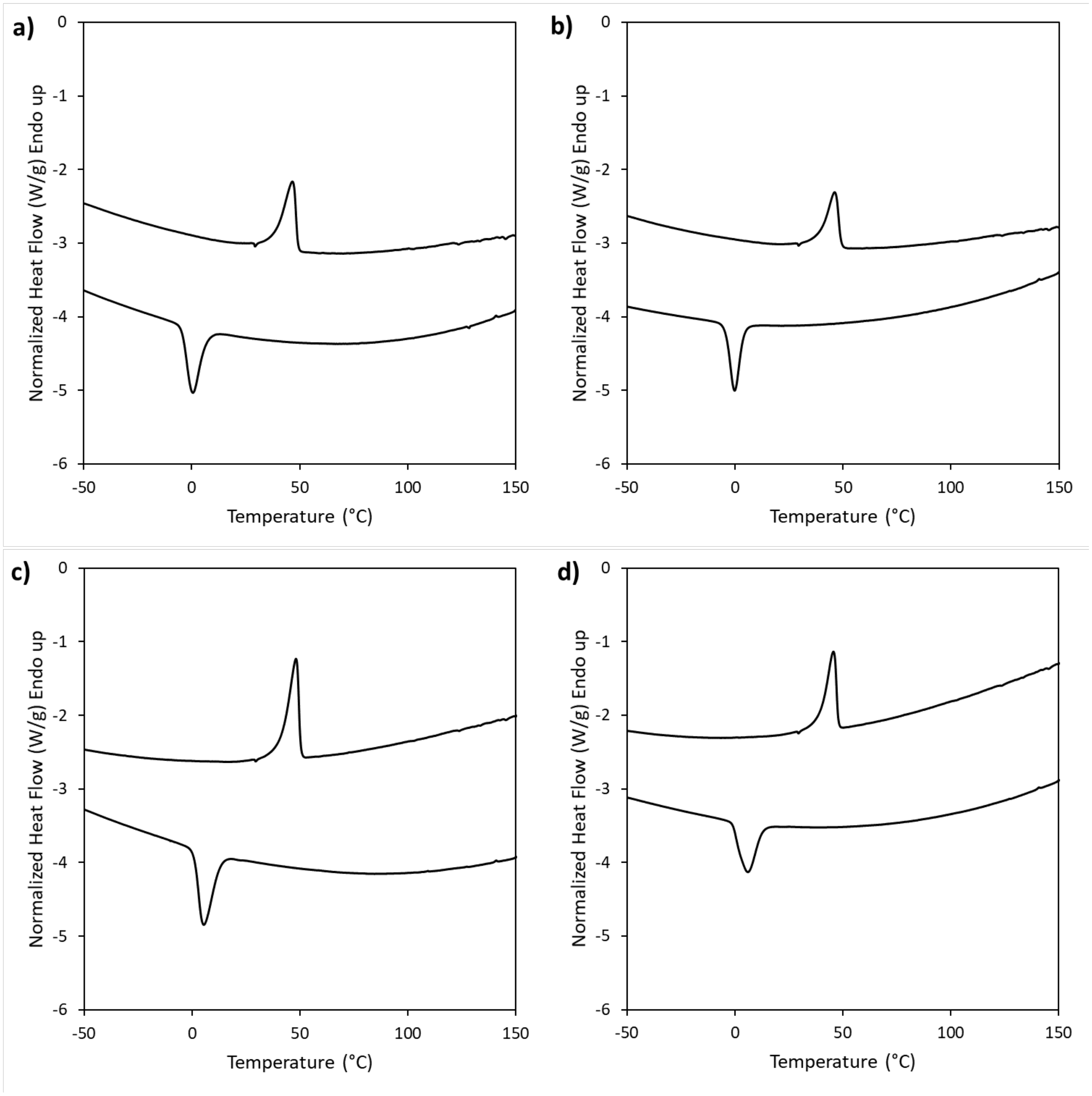

Figure S28. DSC curves of semicrystalline BCN-93 mixed with 10 wt% ZIF-8 (a), 20 wt% ZIF-8 (b), 10 wt% UiO-66 (c) and 20 wt% UiO-66 (d). In all cases, the melting and crystallization peaks are observed.

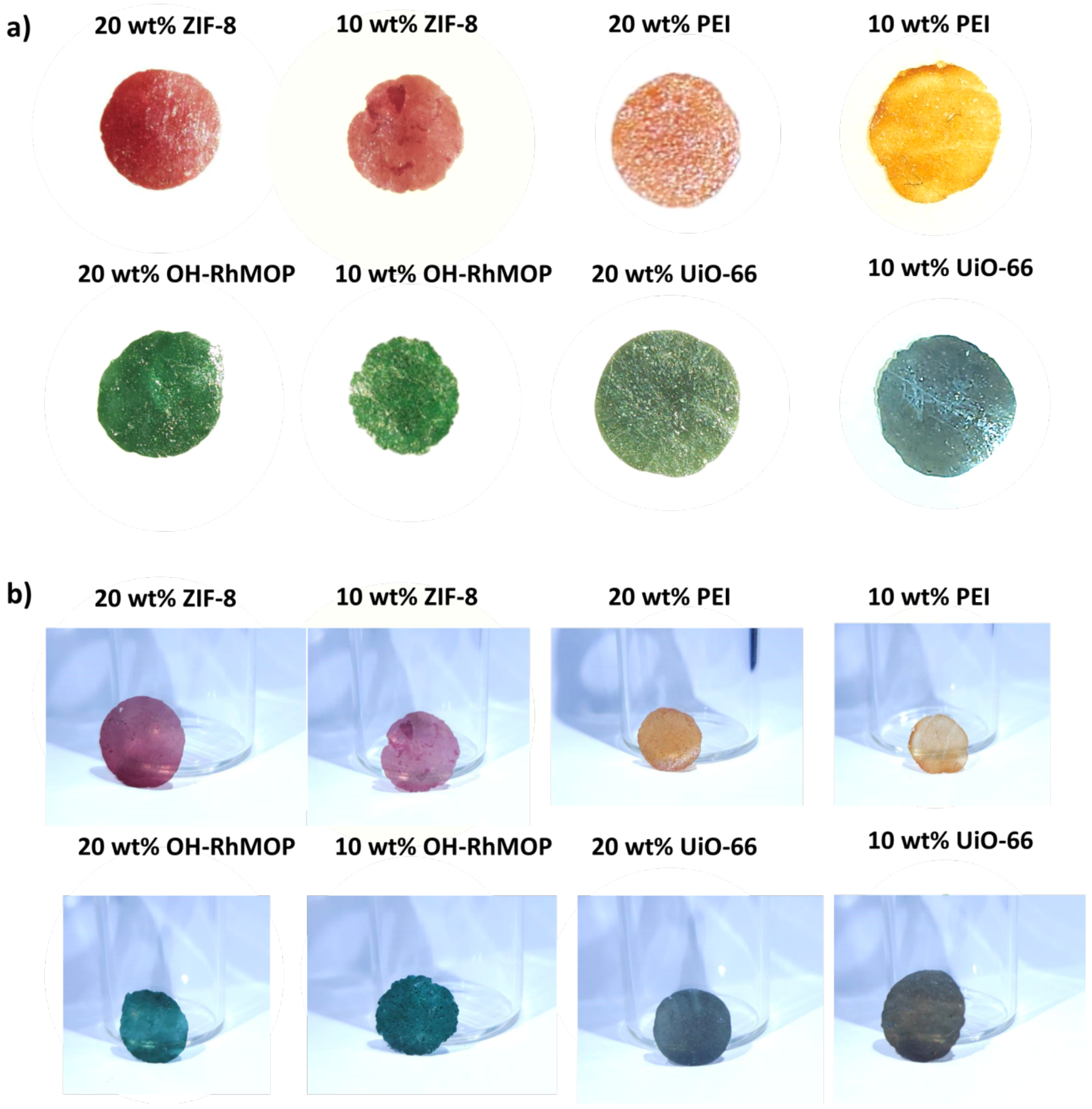

Figure S29. a) Pictures of the top view of the self-standing BCN-93 composite films made with different additives as indicated. b) Pictures of the self-standing BCN-93 composite films made with different additives (as indicated) leaning against a vial with a diameter of 1.5 cm.

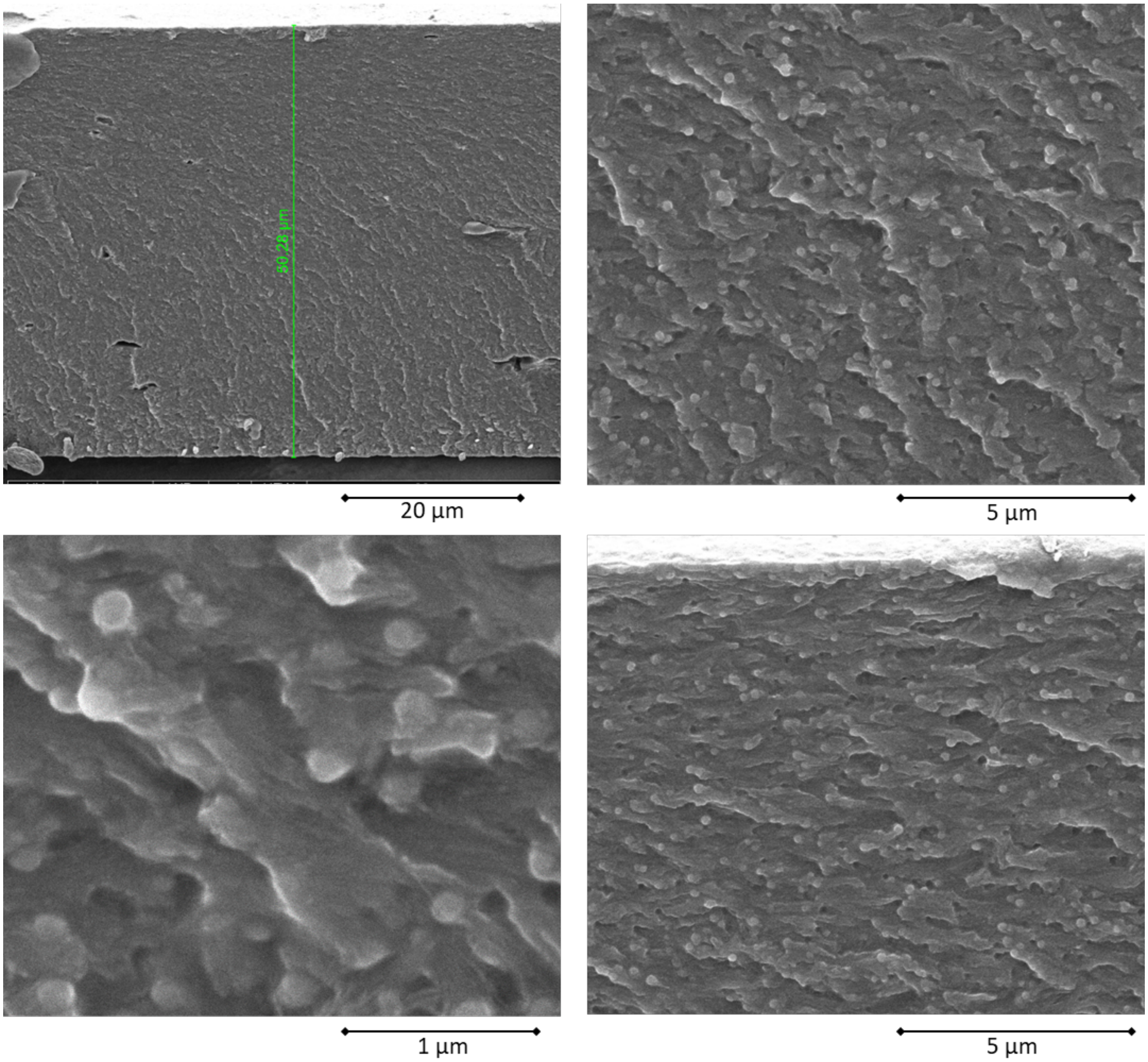

Figure S30. FE-SEM cross-section images of the amorphous BCN-93 composite film doped with 10 wt% of ZIF-8.

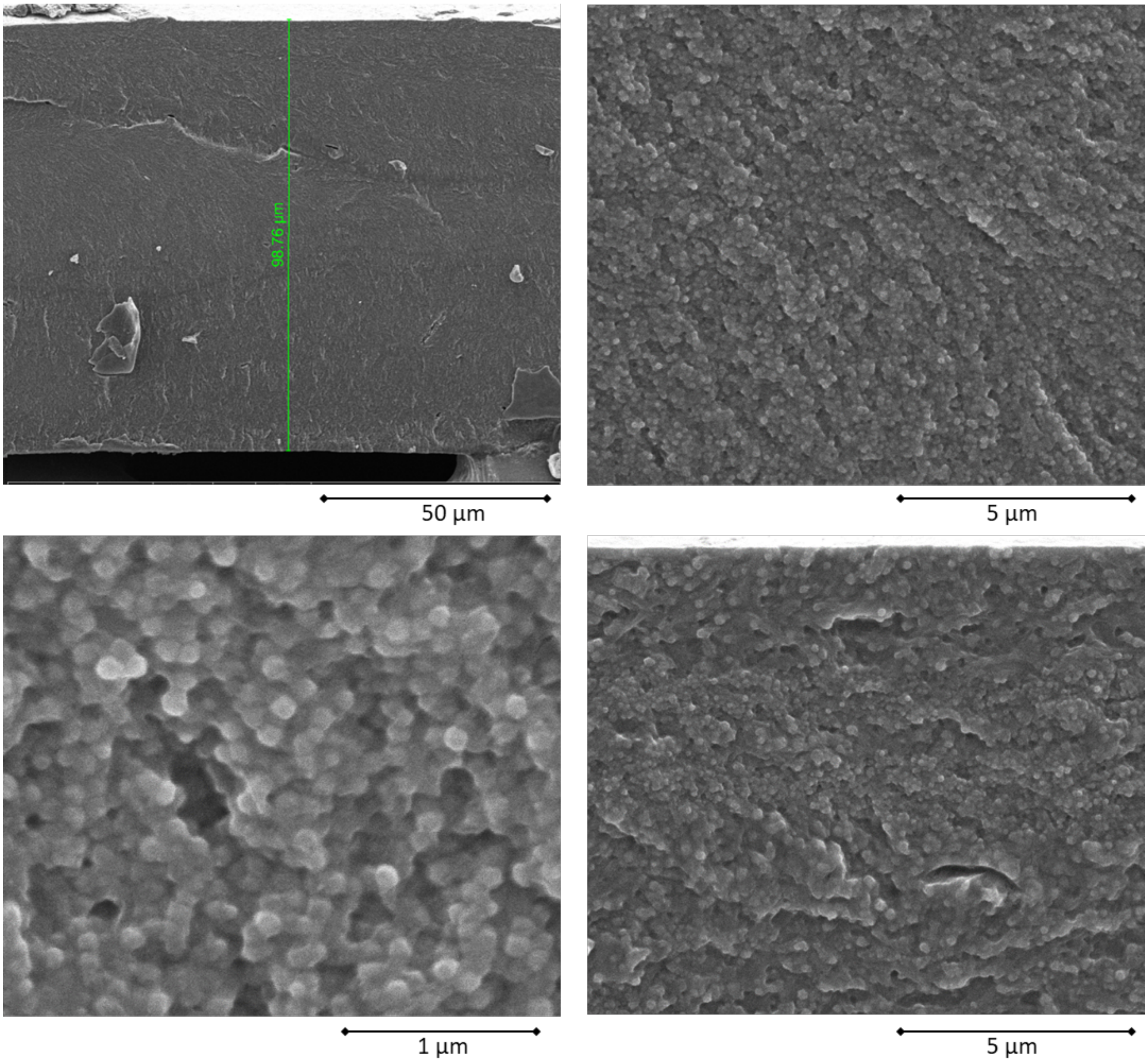

Figure S31. FE-SEM cross-section images of the amorphous BCN-93 composite film doped with 20 wt% of ZIF-8.

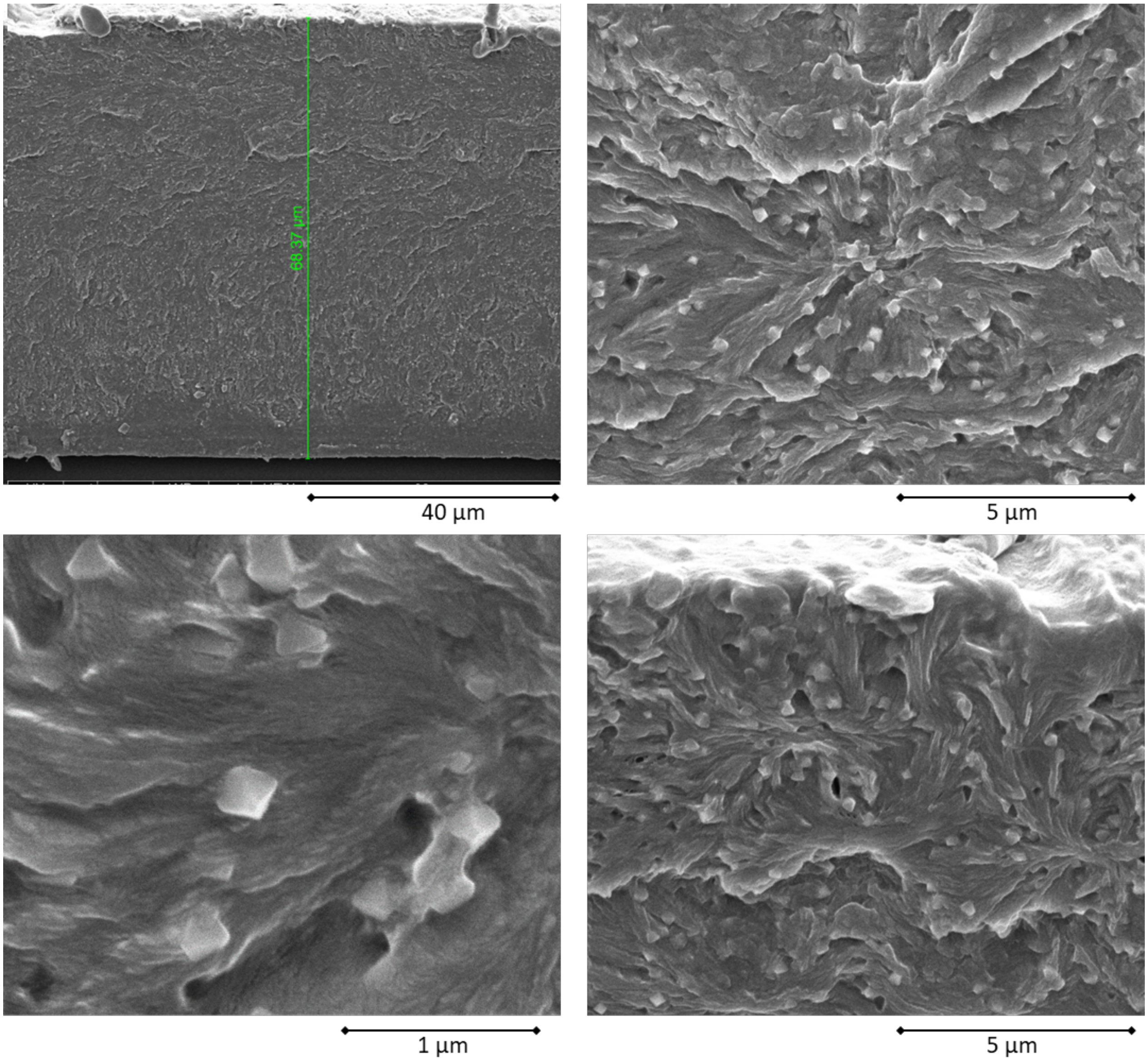

Figure S32. FE-SEM cross-section images of the amorphous BCN-93 composite film doped with 10 wt% of UiO-66.

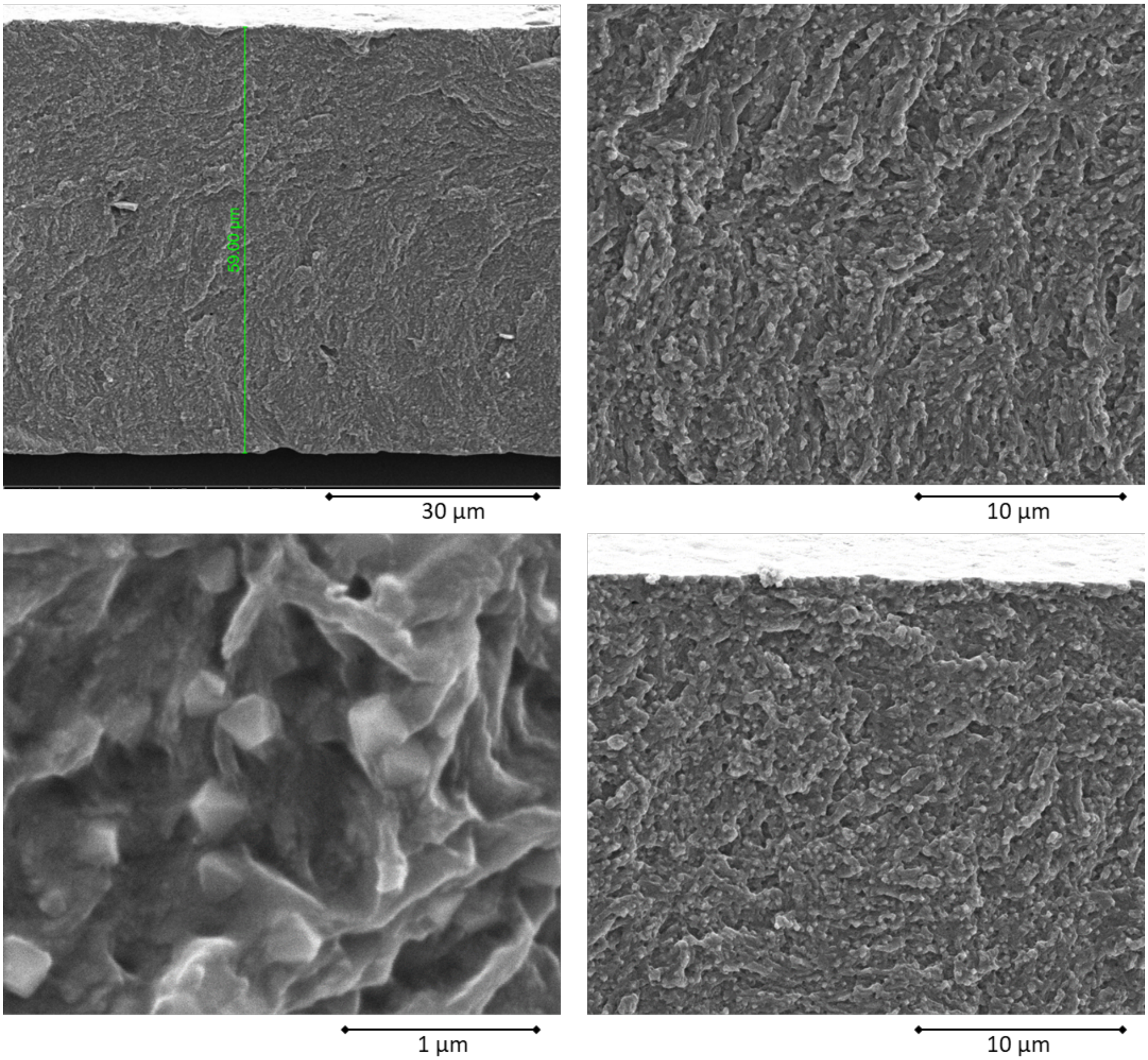

Figure S33. FE-SEM cross-section images of the amorphous BCN-93 composite film doped with 20 wt% of UiO-66.

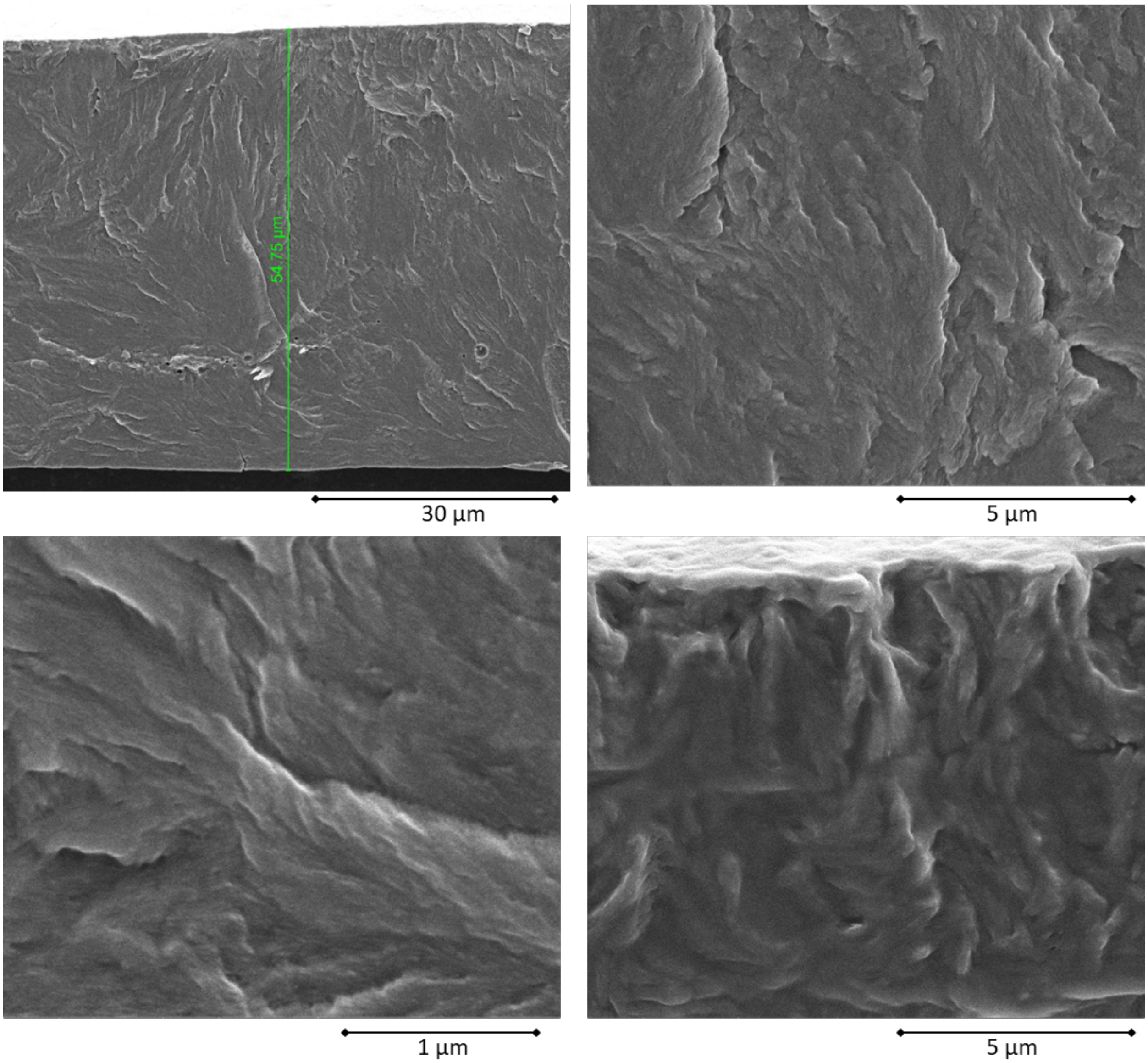

Figure S34. FE-SEM cross-section images of the amorphous BCN-93 composite film doped with 10 wt% of OH-RhMOP.

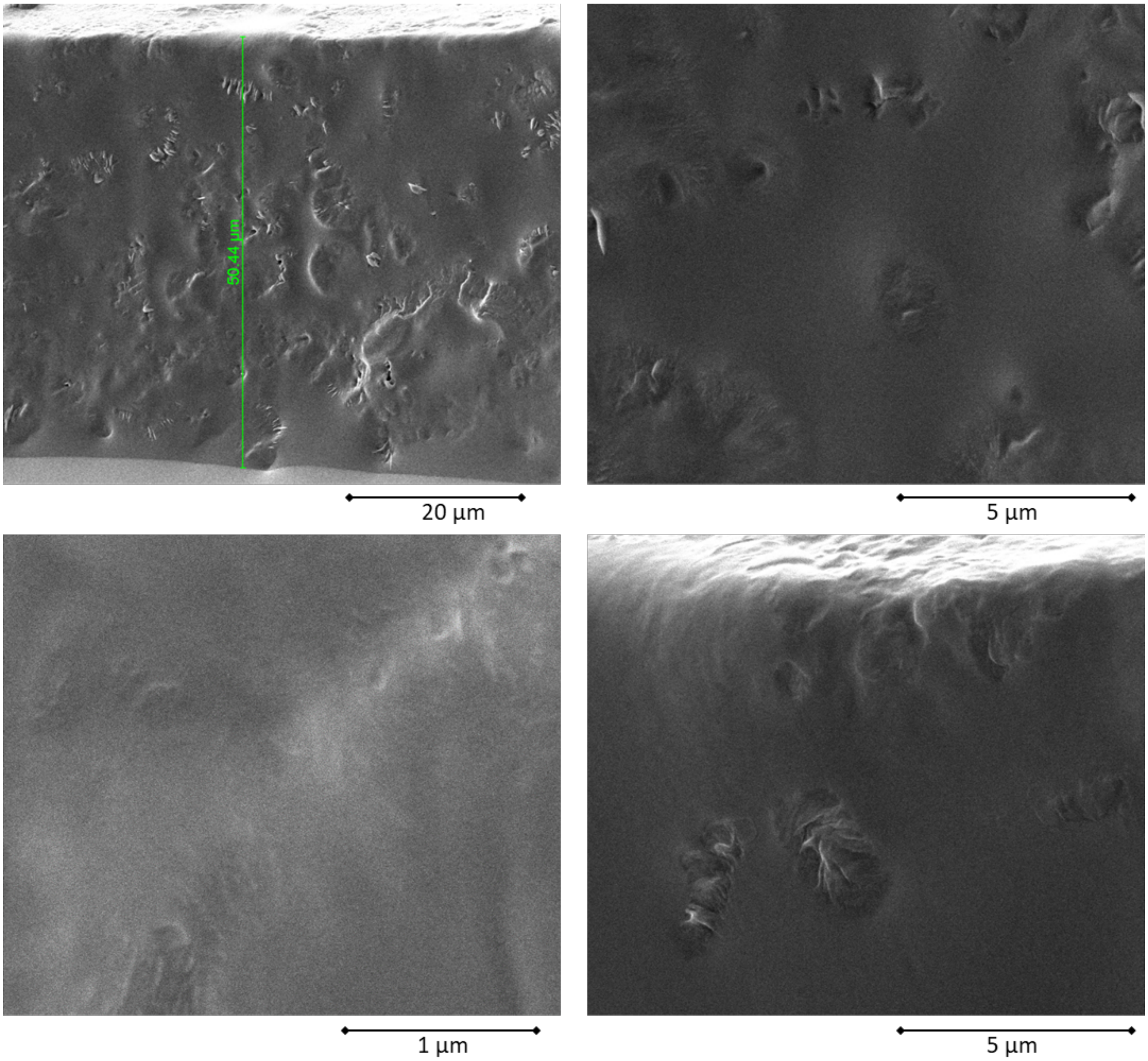

Figure S35. FE-SEM cross-section images of the amorphous BCN-93 composite film doped with 20 wt% of OH-RhMOP.

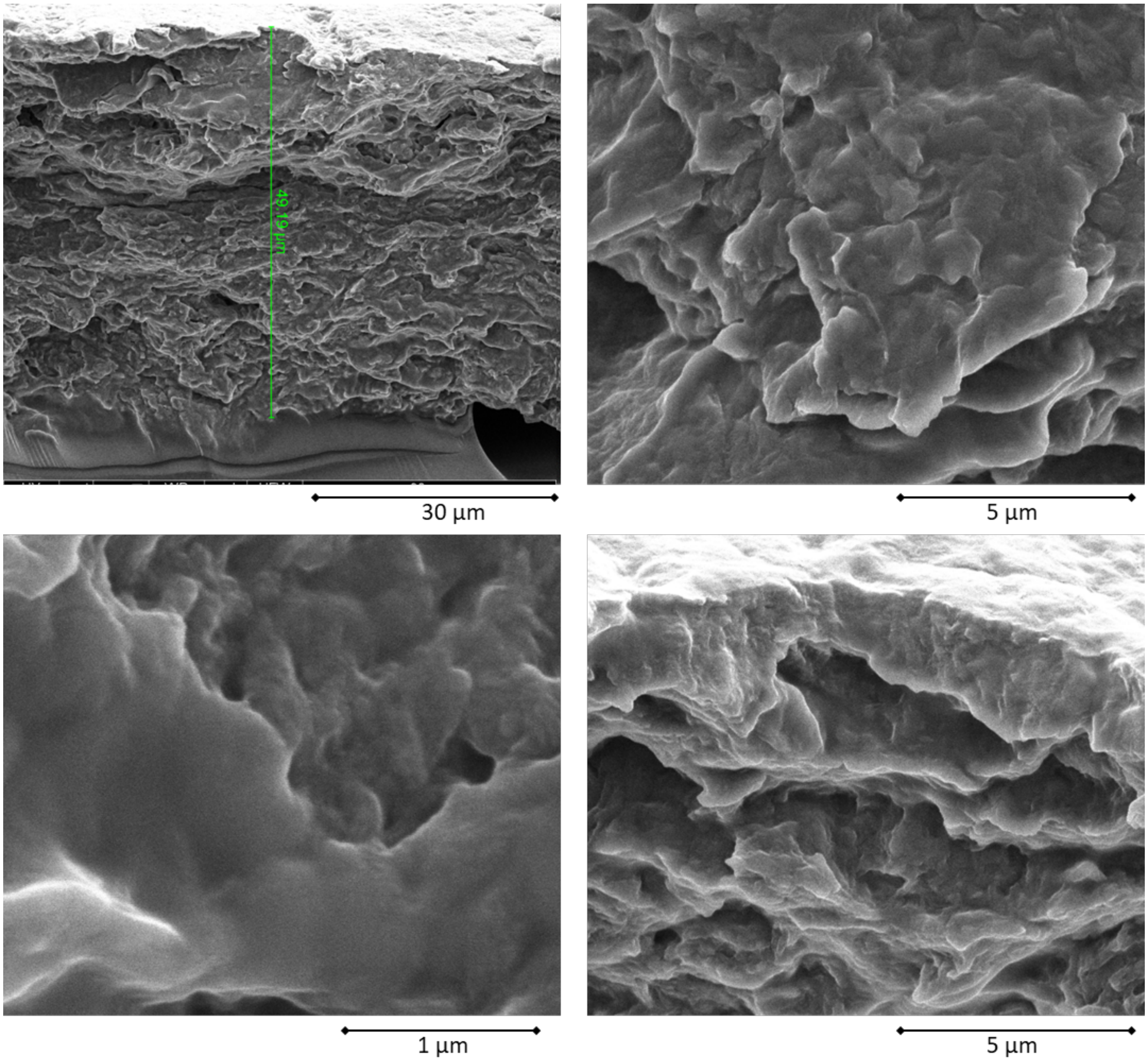

Figure S36. FE-SEM cross-section images of the amorphous BCN-93 composite film doped with 10 wt% of PEI.

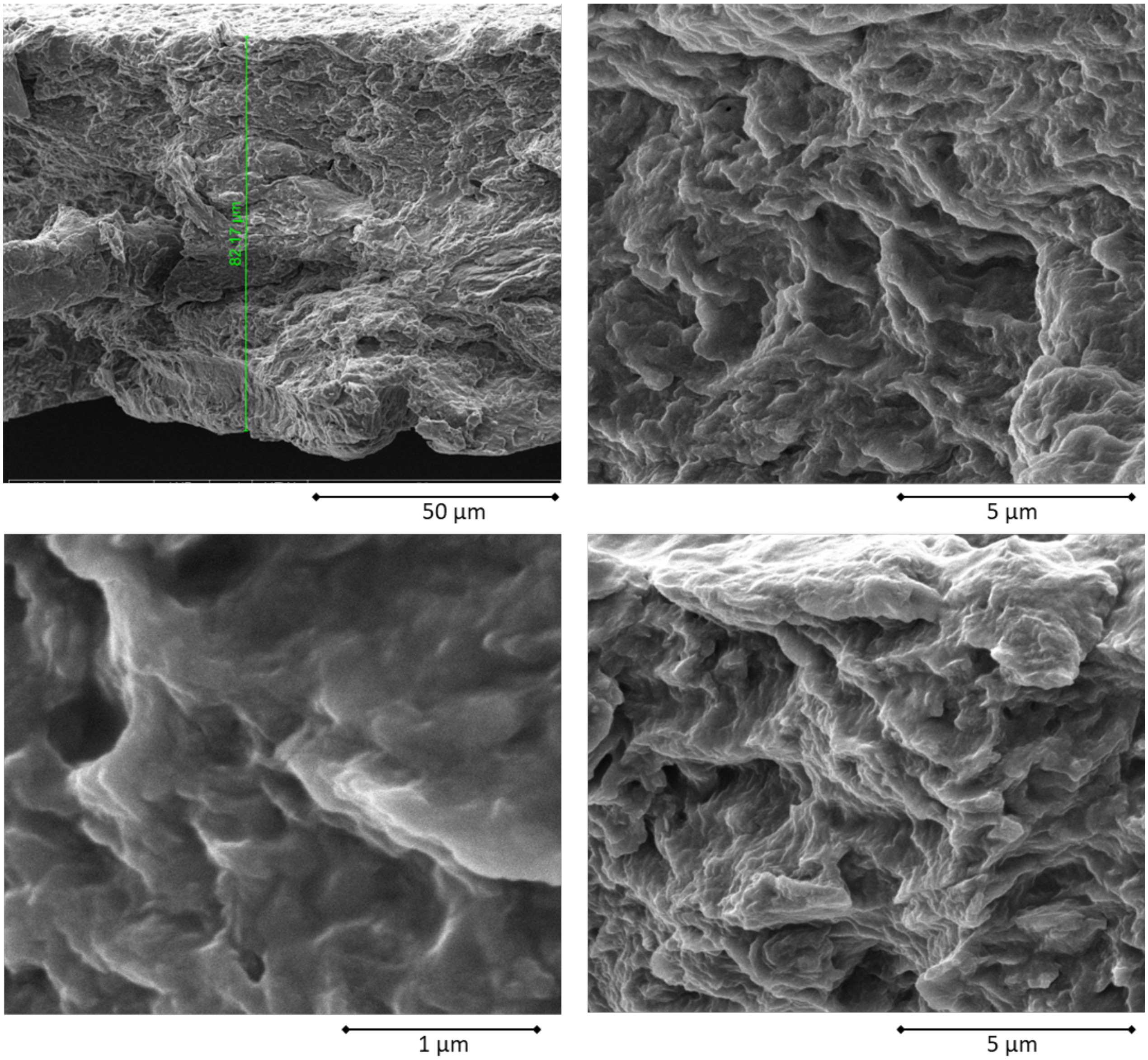

Figure S37. FE-SEM cross-section images of the amorphous BCN-93 composite film doped with 20 wt% of PEI.

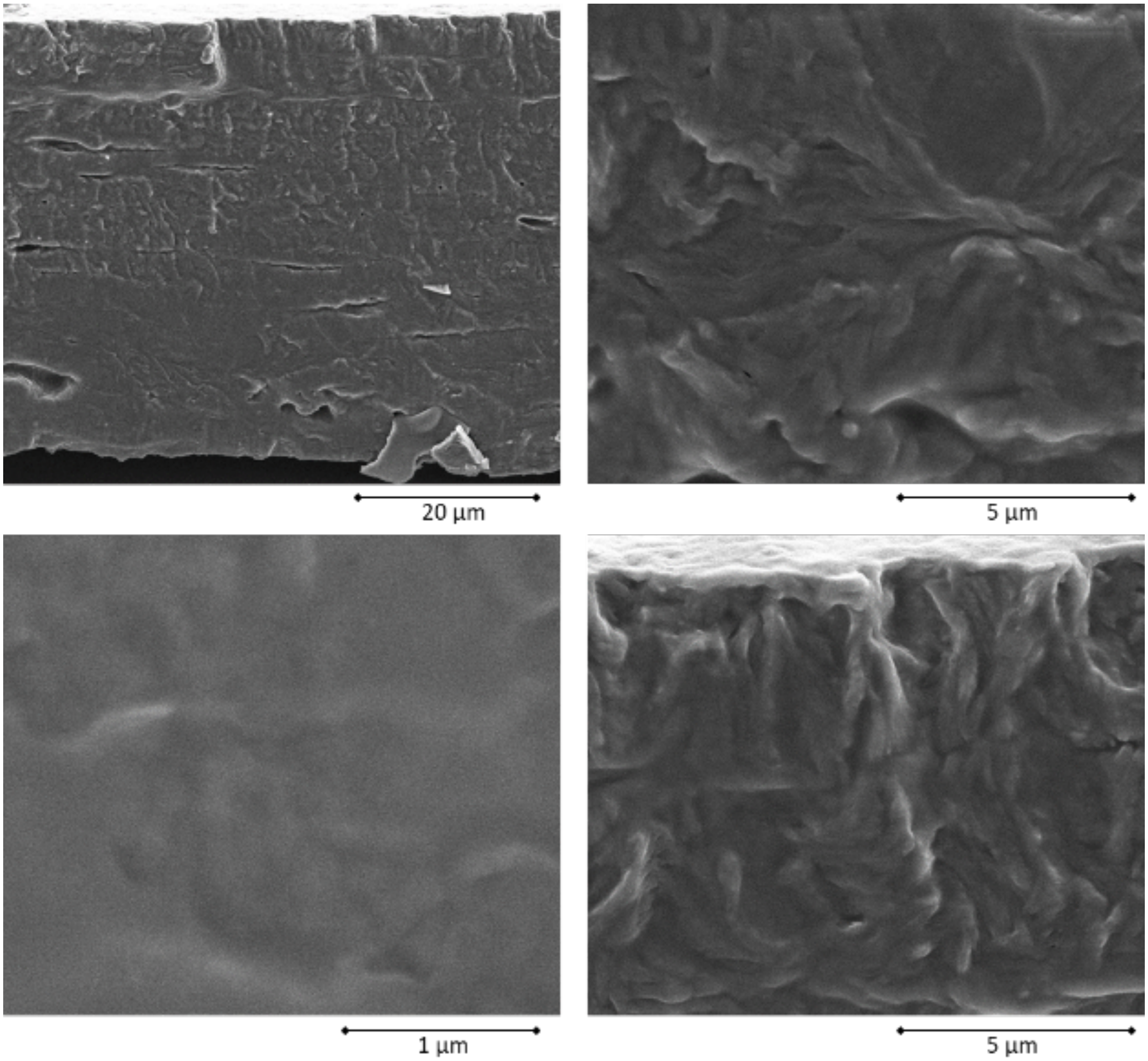

Figure S38. FE-SEM cross-section images of an amorphous BCN-93 film.

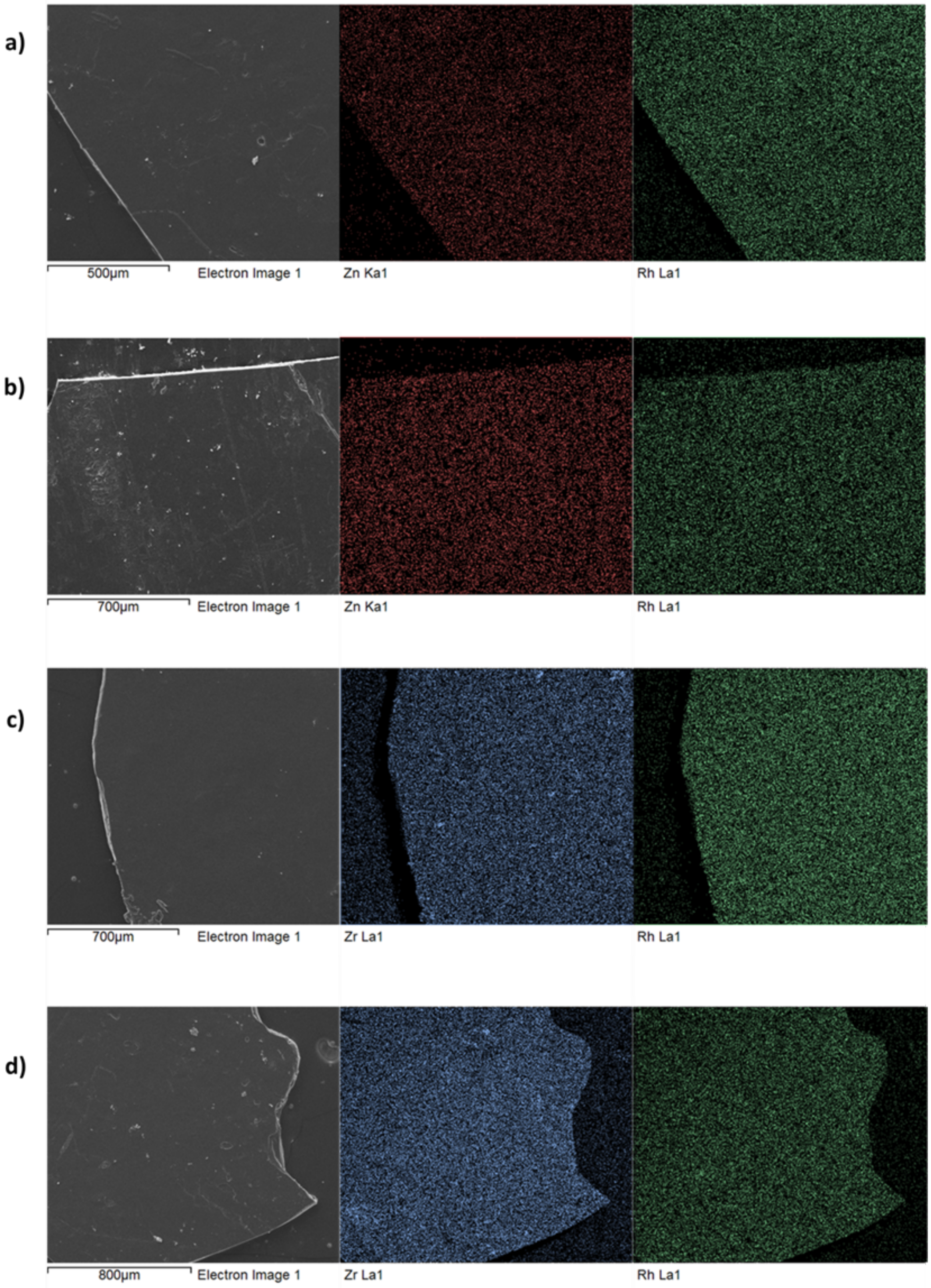

Figure S39. SEM-EDX analysis of mixed-matrix composite films containing (a) 10 wt% ZIF-8, (b) 20 wt% ZIF-8, (c) 10 wt% UiO-66 or (d) 20 wt% UiO-66. Left to right: electron image; Rh (green); Zr (blue); and Zn (red).

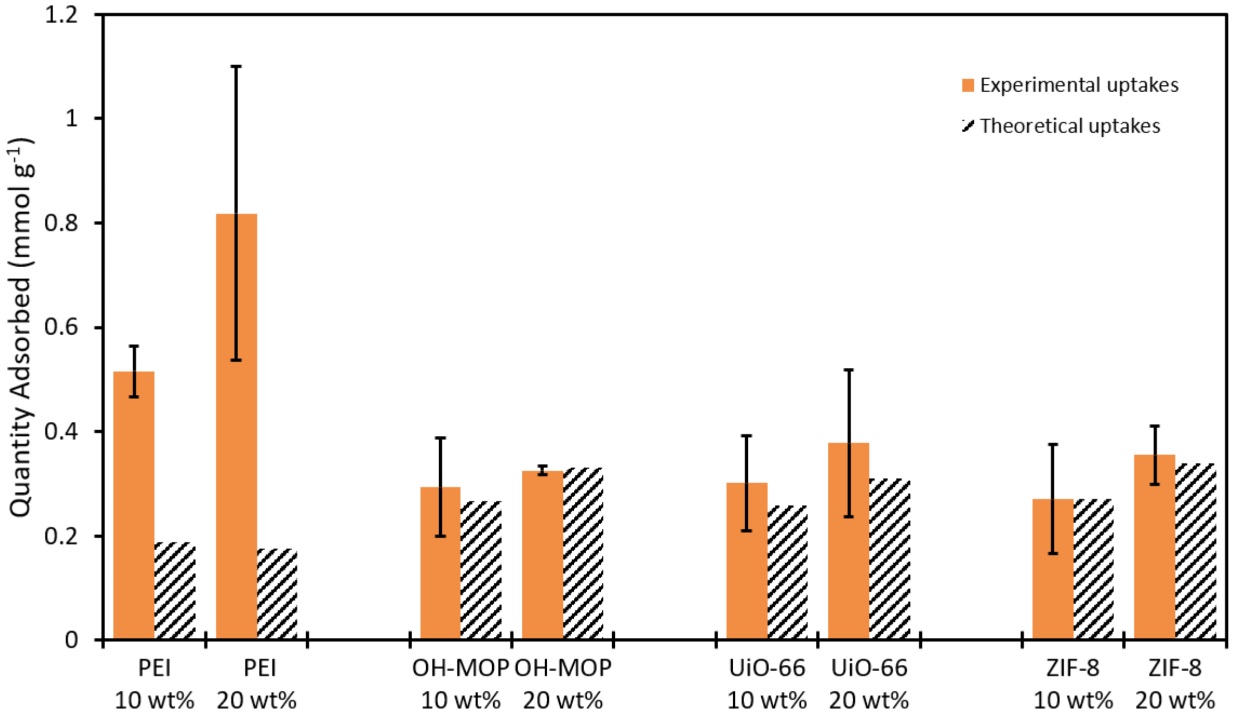

Figure S40. CO_2 uptake at 1 bar and 298 K of the BCN-93 amorphous composite films containing different additives, as indicated. The error bars illustrate the standard deviation obtained from three independent samples. Theoretical uptakes were determined by considering the individual uptake of each component, averaged based on its weight percentage within the composite film. The CO_2 uptake at 1 bar for pure PEI, OH-RhMOP, ZIF-8 and UiO-66 were 0.06 mmol g^{-1}, 0.84 mmol g^{-1}, 0.77 mmol g^{-1} and 0.89 mmol g^{-1}, respectively.

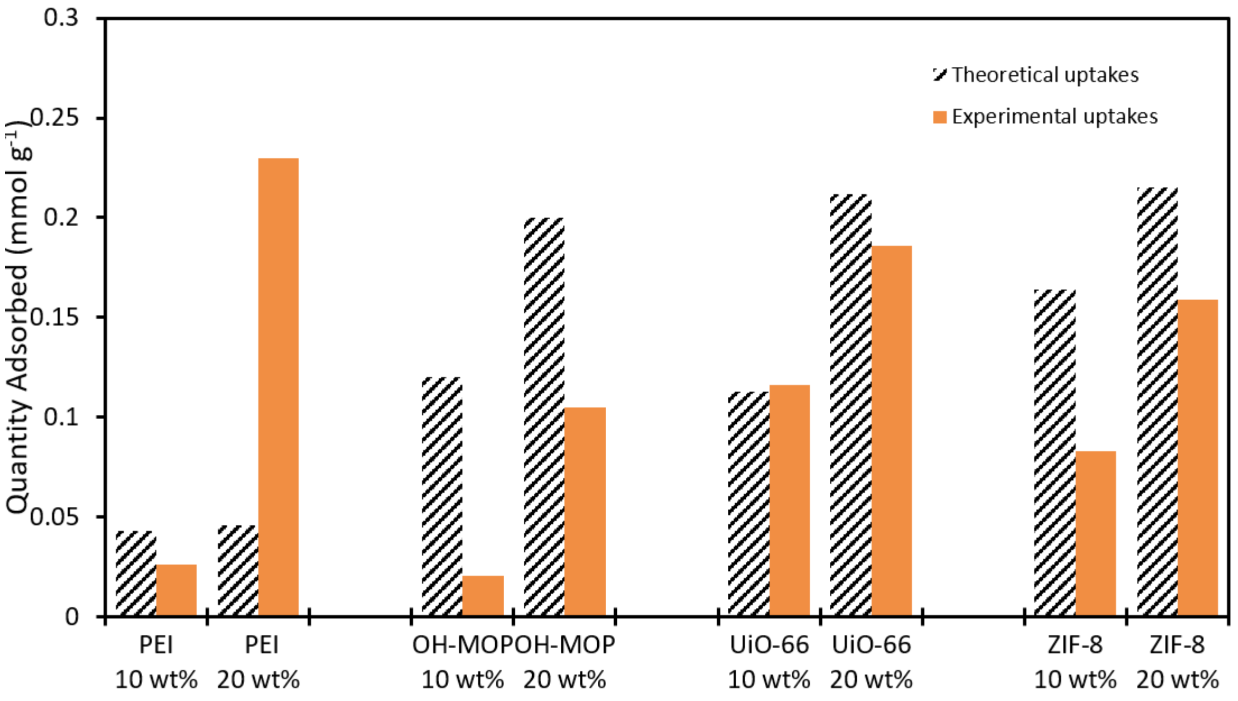

Figure S41. CO_2 uptake at 1 bar and 298 K of composite mixtures containing mPEG-OCH_3 (2 kDa) and different additives. Theoretical uptakes were determined by considering the individual uptake of each component, averaged based on its weight percentage within the composite film. Note that, the CO_2 uptakes observed are below the theoretical values for all porous materials and molecules indicating that linear PEG infiltrates the pore structure of these dopants. In the case of PEI, a positive effect of adding PEG is observed. This behavior is ascribed to the reduced organic viscosity of the PEI-PEG polymer blend as well as a higher accessibility of amine groups in the composite.[12,13] However, the impact of PEG in increasing the efficiency of PEI is much less pronounced than BCN-93.

Table S1. Comparison of the CO_2 uptakes of the most representative meltable materials.

Meltable porous material	Physical state	CO_2 uptake	Measurement Temperature	Reference
ZIF-62 glass<br>**(MOF)**	glass	$10\ cm^3\ g^{-1}$	25 °C	[14]
1-MQG<br>**(coordination polymer)**	melt quenched glass	$18\ cm^3\ g^{-1}$	-75 °C	[15]
a_gZIF-62(Zn)-bim$_x$ (x= 0.35-0.05)<br>**(mixed linker MOF)**	glass	$25\ cm^3\ g^{-1}$ ¬	0 °C	[16]
MOP-1A<br>**(MOP)**	liquid	$1\ cm^3\ g^{-1}$	30 °C	[17]
MOP-1A<br>**(MOP)**	semicrystalline	$0.1\ cm^3\ g^{-1}$	0 °C	[17]
MOP-1A<br>**(MOP)**	supercooled liquid	$0.3\ cm^3\ g^{-1}$	0 °C	[17]
[F-omim][$CF_3(CF_2)_3CO_2$]<br>(**Ionic liquid**)	liquid	$1.6\ cm^3\ g^{-1}$ *	25 °C	[18]
a_gZIF-62(Co)<br>**(MOF)**	glass	$19\ cm^3\ g^{-1}$ ~	0 °C	[19]
a_gZIF-76-mbIm<br>**(MOF)**	glass	$22\ cm^3\ g^{-1}$	20 °C	[20]
18-C-6-PL<br>**(crown ether-ACC)**<br>ACC = Anionic covalent cage	liquid	$11\ cm^3\ g^{-1}$ ֍<br>(at 10 bar)	25 °C	[21]
a_gZIF-UC-7<br>**(MOF)**	glass	$19\ cm^3\ g^{-1}$	0 °C	[22]
a_gZIF-UC-6<br>**(MOF)**	glass	$23\ cm^3\ g^{-1}$	0 °C	[23]
a_g[Zn(Im)$_{1.65}$(bIm)$_{0.35}$]<br>**(MOF)**	glass	$24\ cm^3\ g^{-1}$	0 °C	[24]
a_gZIF-UC-4<br>**(Fluorinated MOF)**	glass	$42\ cm^3\ g^{-1}$	0 °C	[25]
BCN-93<br>**(MOP)**	supercooled liquid	$5\ cm^2\ g^{-1}$	25 °C	This work
BCN-93<br>**(MOP)**	semicrystalline	$5\ cm^2\ g^{-1}$	25 °C	This work

¬ Calculated from 1 mmol g^{-1}

* Calculated from 110 mmol L^{-1} using the density reported at 25 °C (1.7 g ml^{-1})

~ Calculated from 0.75 mmol g^{-1}

֍ Calculated from 0.43 mmol g^{-1}

Table S2. Comparison of the CO_2 uptakes of the most representative mixed matrix composites.

**Mixed matrix composites**	**Physical state**	**CO_2 uptake**	**Measurement Temperature**	**Reference**
(ZIF-8)(ZIF-62)(20/80)<br>**(MOFs)**	glass	31 $cm^3\ g^{-1}$	0 °C	[26]
$(MIL\text{-}53)_{0.6}(a_gZIF\text{-}62)_{0.4}$<br>**(MOFs)**	glass	64 $cm^3\ g^{-1}$ *	0 °C	[27]
PPIL-0.1%(Vis)<br>**(MOP + Ionic Liquid)**	liquid	3.5 $cm^3\ g^{-1}$ #	25 °C	[28]
PL7-10<br>**(MOF + Silicon Oil)**	liquid	5 $cm^3\ g^{-1}$	25 °C	[29]
BCN-93 (20 wt% PEI)<br>**(MOP + Polymer)**	supercooled liquid	25 $cm^2\ g^{-1}$	25 °C	This work
BCN-93 (20 wt% OH-MOP)<br>**(MOP + MOP)**	supercooled liquid	9 $cm^2\ g^{-1}$	25 °C	This work
BCN-93 (20 wt% ZIF-8)<br>**(MOP + MOF)**	supercooled liquid	9 $cm^2\ g^{-1}$	25 °C	This work
BCN-93 (20 wt% UiO-66)<br>**(MOP + MOF)**	supercooled liquid	9 $cm^2\ g^{-1}$	25 °C	This work

* Calculated from 2.55 mmol g^{-1}

\# Calculated from 0.14 mmol g^{-1}

S4. References

[1] Albalad, J.; Carné-Sánchez, A.; Grancha, T.; Hernández-López, L.; Maspoch, D. Protection Strategies for Directionally-Controlled Synthesis of Previously Inaccessible Metal–Organic Polyhedra (MOPs): The Cases of Carboxylate- and Amino-Functionalised Rh(ii)-MOPs. *Chem. Commun.* **2019**, *55*, 12785–12788.

[2] Avci, C.; Imaz, I.; Carné-Sánchez, A.; Pariente, J. A.; Tasios, N.; Pérez-Carvajal, J.; Alonso, M. I.; Blanco, A.; Dijkstra, M.; López, C.; Maspoch, D. Self-assembly of Polyhedral Metal–Organic Framework Particles into Three-Dimensional Ordered Superstructures. *Nat. Chem.* **2018**, *10*, 78–84.

[3] Erdosy, D. P.; Wenny, M. B.; Cho, J.; DelRe, C.; Walter, M. V.; Jiménez-Ángeles, F.; Qiao, B.; Sanchez, R.; Peng, Y.; Polizzotti, B. D.; Olvera de la Cruz, M.; Mason, J. Microporous Water with High Gas Solubilities. *Nature*, **2022**, *608*, 712–718.

[4] Carné-Sánchez, A.; Albalad, J.; Grancha, T.; Imaz, I.; Juanhuix, J.; Larpent, P.; Furukawa, S.; Maspoch, D. Post-Synthetic Covalent and Coordination Functionalization of Rhodium(II)-based Metal-Organic Polyhedra. *J. Am. Chem. Soc.*, **2019**, *141*, 4094–4102.

[5] Phillips, J. C.; Braun, R.; Wang, W.; Gumbart, J.; Tajkhorshid, E.; Villa, E.; Chipot, C.; Skeel, R. D.; Kalé, L.; Schulten, K. Scalable Molecular Dynamics with NAMD. *J. Comput. Chem*. **2005**, *26*, 1781–1802.

[6] Vanommeslaeghe, K.; Prabhu Raman, E.; MacKerell, A. D. Automation of the CHARMM General Force Field (CGenFF) II: Assignment of Bonded Parameters and Partial Atomic Charges. *J. Chem. Inf. Model*. **2012**, *52*, 3155–3168.

[7] Vanommeslaeghe K.; MacKerell, A. D. Automation of the CHARMM General Force Field (CGenFF) I: Bond Perception and Atom Typing. *J. Chem. Inf. Model*. **2012**, *12*, 3144–3154.

[8] Hernández-López, L.; Martínez-Esaín, J.; Carné-Sánchez, A.; Grancha, T.; Faraudo, J.; Maspoch, D. Steric Hindrance in Metal Coordination Drives the Separation of Pyridine Regioisomers Using Rhodium(II)-Based Metal–Organic Polyhedra. *Angew. Chem. Int. Ed*. **2021**, *60*, 11406–11413.

[9] Hernández-López, L.; von Baeckmann, C.; Martínez-Esaín, J.; Cortés-Martínez, A.; Faraudo, J.; Caules, C.; Parella, T.; Maspoch, D.; Carné-Sánchez, A. (Bio)Functionalisation of Metal–Organic Polyhedra by Using Click Chemistry. *Chem. Eur. J*. **2023**, *29*, e202301945.

[10] Humphrey, W.; Dalke, A.; Schulten, K. VMD – Visual Molecular Dynamics. *J. Molec. Graphics* **1996**, *14*, 33-38.

[11] Michaud-Agrawal, N.; Denning, E. J.; Woolf, T. B.; Beckstein, O. MDAnalysis: A Toolkit for the Analysis of Molecular Dynamics Simulations. *J. Comput. Chem.* **2011**, *32*, 2319-2327.

[12] Wang, J.; Long, D.; Zhou, H.; Chen, Q.; Liua, X.; Ling, L.; Surfactant Promoted Solid Amine Sorbents for CO_2 Capture. *Energy Environ. Sci*., **2012**,*5*, 5742-5749.

[13] Heydari-Gorji, A.; Belmabkhout, Y.; Sayari. A. Polyethylenimine-Impregnated Mesoporous Silica: Effect of Amine Loading and Surface Alkyl Chains on CO_2 Adsorption. *Langmuir* **2011***, 27,* 12411–12416.

[14] Wang, Y.; Jin, H.; Ma, Q.; Mao, H.; Feldhoff, A.; Cao, X.; Li, Y.; Pan, F.; Jiang, Z. A MOF Glass Membrane for Gas Separation. *Angew. Chem. Int. Ed*. **2020**, *59*, 4365-4369.

[15] Das, C.; Ogawa, T.; Horike, S. Stable Melt Formation of 2D Nitrile-Based Coordination Polymer and Hierarchical Crystal–Glass Structuring. *Chem. Commun*. **2020**, *56*, 8980-8983.

[16] Frentyel-Beyme, L.; Kloß, M.; Kolodzeiski, P.; Pallach, R.; Henke, S. Meltable Mixed-Linker Zeolitic Imidazolate Frameworks and Their Microporous Glasses: From Melting Point Engineering to Selective Hydrocarbon Sorption. *J. Am. Chem. Soc*. **2019**, *141*, 12362–12371.

[17] Han, P-C.; Chuang, C-H.; Lin, S-W.; Wang, Z.; Kuzumoto, M.; Xiang, X.; Tokuda, S.; Tateishi, T.; Legrand, A.; Tsang, M. Y.; Yang, H.-C.; Wu, K. C-W.; Urayama, K.; Kang, D-Y.; Furukawa, S. Porous Soft Materials with Liquid-Glass-Crystal Interconvertibility based on Metal-Organic Polyhedra. *ChemRxiv*. **2024**; doi:10.26434/chemrxiv-2024-sns8n.

[18] Wenny, M. B.; Walter, M. V.; Slavney, A. H.; Mason, J. A. Generalizable Synthesis of Highly Fluorinated Ionic Liquids. *J. Phys. Chem. B* **2023**, *127*, 2028−2033.

[19] Frentyel-Beyme, L.; Kloß, M.; Pallach, R.; Salamon, S.; Moldenhauer, H.; Landers, J.; Wende, H.; Debus, J.; Henke, S. Porous Purple Glass – a Cobalt Imidazolate Glass with Accessible Porosity from a Meltable Imidazolate Framework. *J. Mater. Chem. A*, **2019**, *7*, 985-990.

[20] Zhou, C.; Longley, L.; Krajnc, A.; Smales, G. J.; Qiao, A.; Erucar, I.; Doherty, C. M.; Thornton, A. W.; Hill, A. J.; Ashling, C. W.; Qazvini, O. T.; Lee, S. J.; Chater, P. A.; Terrill, N. J.; Smith, A. J.; Yue, Y.; Mali, G.; Keen, D. A.; Telfer, S. G.; Bennett. T. D. Metal-Organic Framework Glasses with Permanent Accessible Porosity. *Nat. Commun*. **2018**, *9*, 5042.

[21] Jie, K.; Onishi, N.; Schott, J. A.; Popovs, I.; Jiang, D.; Mahurin, S.; Dai, S. Transforming Porous Organic Cages into Porous Ionic Liquids via a Supramolecular Complexation Strategy. *Angew. Chem. Int. Ed*. **2019**, *59*, 2268-2272.
[22] Bumstead, A. M.; Castillo-Blas, C.; Pakamore, I.; Thorne, M. F.; Sapnik, A. F.; Chester, A. M.; Robertson, G.; Irving, D. J. M.; Chater, P. A.; Keen, D. A.; Forgan, R. S.; Bennett, T. D. Formation of a Meltable Purinate Metal–Organic Framework and its Glass Analogue. *Chem. Commun*. **2023**, *59*, 732-735.
[23] Bumstead, A. M.; Pakamorė, I.; Richards, K. D.; Thorne, M. F.; Boyadjieva, S. S.; Castillo-Blas, C.; McHugh, L. N.; Sapnik, A. F.; Keeble, D. S.; Keen, D. A.; Evans, R.C.; Forgan, R.S.; Bennett, T. D. Post-Synthetic Modification of a Metal–Organic Framework Glass. *Chem. Mater*. **2022**, *34*, 2187–2196.
[24] Thorne, M. F.; Sapnik, A. F.; McHugh, L. N.; Bumstead, A. M.; Castillo-Blas, C.; Keeble, D. S.; Diaz Lopez, M.; Chater, P. A.; Keen, D. A.; Bennett, T. D. Glassy Behaviour of Mechanically Amorphised ZIF-62 Isomorphs. *Chem. Commun.*, **2021**, *57*, 9272–9275.
[25] Hou, J.; Ríos Gomez, M. L.; ̌Krajnc, A.; McCaul, A.; Li, S.; Bumstead, A. M.; Sapnik, A. F.; Deng, Z.; Lin, R.; Chater, P. A.; Keeble, D. S.; Keen, D. A.; Appadoo, D.; Chan, B.; Chen, V.; Mali, G.; Bennett, T. D. Halogenated Metal−Organic Framework Glasses and Liquids. *J. Am. Chem. Soc*. **2020**, *142*, 3880−3890.
[26] Longley, L.; Collins, S. M.; Li, S.; Smales, G. J.; Ercuar, I.; Qiao, A.; Hou, J.; Doherty, C. M.; Thornton, A. W.; Hill, A. K.; Yu, X.; Terrill, N.J.; Smith, A. J.; Cohen, S. M.; Midgley, P. A.; Keen, D. A.; Telfer, S. G.; Bennett, T. D. Flux Melting of Metal–Organic Frameworks. *Chem. Sci*., **2019**,*10*, 3592-3601.
[27] Ashling, C. A.; Johnstone, D. N.; Widmer, R. N.; Hou, J.; Collings, S. M.; Sapnik, A. F.; Bumstead, A. M.; Midgley, P. A.; Chater, P. A.; Keen, D. A.; Bennett, T. D. Synthesis and Properties of a Compositional Series of MIL-53(Al) Metal–Organic Framework Crystal-Glass Composites. *J. Am. Chem. Soc*. **2019**, *141*, 15641−15648.
[28] Dinker, M. K.; Zhao, K.; Dai, Z.; Ding, L.; Liu, X.-Q.; Sun, L.-B. Porous Liquids Responsive to Light. *Angew. Chem. Int. Ed.* **2022**, *61*, e202212326.
[29] Koutsianos, A.; Pallach, R.; Frentzel-Beyme, L.; Das, C.; Paulus, M.; Ternemann, C. S.; Henke, S. Breathing Porous Liquids based on Responsive Metal-Organic Framework Particles. *Nat. Comm*. **2023**, *14*, 4200.
